# Evolutionary dynamics of canine kobuvirus in Vietnam and Thailand reveal the evidence of viral ability to evade host immunity

**DOI:** 10.1038/s41598-024-62833-2

**Published:** 2024-05-27

**Authors:** Tin Van Nguyen, Tanit Kasantikul, Chutchai Piewbang, Somporn Techangamsuwan

**Affiliations:** 1 The International Graduate Program of Veterinary Science and Technology (VST), Faculty of Veterinary Science, Chulalongkorn University, Bangkok, Thailand; 2 Faculty of Animal Science and Veterinary Medicine, Nong Lam University, Ho Chi Minh City, Vietnam; 3 Animal Virome and Diagnostic Development Research Unit, Faculty of Veterinary Science, Chulalongkorn University, Bangkok, Thailand; 4 Veterinary Diagnostic Laboratory, Department of Pathobiology and Diagnostic Investigation, College of Veterinary Medicine, Michigan State University, East Lansing, MI USA; 5 Department of Pathology, Faculty of Veterinary Science, Chulalongkorn University, Bangkok, 10330 Thailand

**Keywords:** B-cell epitope predictions, Canine kobuvirus, Phylogenetic tree, Selective pressure analysis, Thailand, Vietnam, Infectious diseases, Viral infection, Viral genetics

## Abstract

Canine kobuvirus (CaKoV) is a pathogen associated with canine gastrointestinal disease (GID). This study examined 327 rectal swabs (RS), including 113 from Vietnam (46 healthy, 67 with GID) and 214 from Thailand (107 healthy and 107 with GID). CaKoV was detected in both countries, with prevalences of 28.3% (33/113) in Vietnam and 7.9% (17/214) in Thailand. Additionally, CaKoV was found in both dogs with diarrhea and healthy dogs. CaKoV was mainly found in puppies under six months of age (30.8%). Co-detection with other canine viruses were also observed. The complete coding sequence (CDS) of nine Vietnamese and four Thai CaKoV strains were characterized. Phylogenetic analysis revealed a close genetic relationship between Vietnamese and Thai CaKoV strains, which were related to the Chinese strains. CDS analysis indicated a distinct lineage for two Vietnamese CaKoV strains. Selective pressure analysis on the viral capsid (VP1) region showed negative selection, with potential positive selection sites on B-cell epitopes. This study, the first of its kind in Vietnam, provides insights into CaKoV prevalence in dogs of different ages and healthy statuses, updates CaKoV occurrence in Thailand, and sheds light on its molecular characteristics and immune evasion strategies.

## Introduction

Canine kobuvirus (CaKoV) was initially discovered in diarrheal fecal samples from dogs in the United States in 2011, using next-generation sequencing (NGS) methods^1,2^. CaKoV is characterized as a small, nonenveloped, icosahedral virus with a single-stranded, positive-sense RNA genome, approximately 30 nm in diameter. Taxonomically, it belongs to the *Kobuvirus* genus within the *Picornaviridae* family^3–5^. Currently, the *Kobuvirus* genus comprises six species (*Aichivirus* A–F): *Aichivirus* A (human kobuvirus), *Aichivirus* B (bovine kobuvirus), *Aichivirus* C (porcine kobuvirus), *Aichivirus* D (kagovirus 1), *Aichivirus* E (rabbit picornavirus) and *Aichivirus* F (bat kobuvirus)^6–9^. CaKoV belongs to the *Aichivirus* A species, along with human kobuvirus^10^, murine kobuvirus^11^, kathmandu wastewater kobuvirus^12^, rolling kobuvirus^13^, and feline kobuvirus^14^.

Similar to other Aichi viruses, the genome of CaKoV ranges from 8.1 to 8.2 kb and follows a specific organization: (1) a 5′ untranslated region (UTR) with an internal ribosome entry site (IRES) facilitating direct translation of the polyprotein, (2) the viral genome protein (VPg—a small covalently linked polypeptide with the 5′UTR), (3) a single open reading frame (ORF) consisting of 7332–7341 nucleotides (nt), followed by (4) the 3′ UTR region, and (5) the poly (A) tail. This single ORF encodes a polyprotein of 2442–2475 amino acids (aa), subject to post-translational cleavage^1,2^. The polyprotein comprises a nonstructural leader protein (L); the P1 region (encoding three structural proteins VP0, VP3 and VP1); the P2 and the P3 regions (encoding seven nonstructural proteins 2A–2C and 3A–3D, respectively)^3^. CaKoV’s structural proteins construct the virion capsid, facilitating virus particles adhesion and entry into host cells. In contrast, the nonstructural proteins and mediators control RNA replication polyprotein cleavage, and virion assembly within infected cells^3^. In the genetic structure of CaKoV, the 3D segment acts as the viral RNA-dependent RNA polymerase, representing a conserved region essential for kobuvirus replication. On the other hand, the viral VP1 protein, the most variable structural region, plays a crucial role and is the most exposed part of the virus in terms of immunodominance^7,15^. The 3C protein, characterized by conserved motifs, functions as a cysteine protease, mediating polyprotein cleavages^15^. The nonstructural L protein, lacking self-catalytic activity and protease function, likely participates in viral RNA replication and encapsidation^16^. Additionally, the nonstructural 2A protein, featuring conserved motifs like H-box/NC and a transmembrane domain, associates with cellular proteins of the H-rev107 family and regulates cell proliferation^17^, as well as viral RNA replication^18^.

Following its initial detection in the USA, CaKoV has been identified in various countries, including Italy^19^, the United Kingdom^20^, China^21–24^, South Korea^25,26^, Japan^27^, Thailand^28^, Brazil^29^ and Germany^30^. The pathological role of CaKoV in canine gastrointestinal diseases (GID) remains a topic of debate^2,21,27,31^. Furthermore, CaKoV has been detected in extraintestinal organs such as the liver, lungs, brain, and tonsils of puppies^32^. It was also found in various organs including stomach, brain, lungs, bladder, trachea, and intestinal lymph nodes of infected foxes^30^.

Genetic recombination, a well-documented phenomenon in many picornavirus genera, plays a significant role in their diversification^15,33^. However, recombination events among CaKoVs were reported in only one recent study^34^. Therefore, information regarding the genetic characterization, recombination, and bioinformatics analyses of CaKoV is still very limited, especially in countries that have not been studied.

Therefore, the purpose of this study was to investigate the presence of CaKoV in rectal swab (RS) samples collected from dogs in Vietnam and Thailand. The study also analyzed risk factors, and explored the relationship between clinical manifestations related to CaKoV-positive dogs. Furthermore, the genetic and bioinformatic analyses of the CaKoV strains obtained in Vietnam and Thailand provided additional crucial information for understanding CaKoV evolution, as well as assessing the impact of selective pressure on the host's immune response.

## Results

### Prevalence of CaKoV detection in domestic dogs in Vietnam and Thailand

CaKoV was identified in RS samples collected from dogs in both Vietnam and Thailand, exhibiting a prevalence of 28.3% (33/113) and 7.9% (17/214), respectively. Furthermore, CaKoV was presented in RS samples from two categories of surveyed dogs: those without diarrhea (8.7% and 6.5%) and those with diarrhea (41.8% and 9.3%), in Vietnam and Thailand, respectively. The amalgamated data from both countries indicated that the group of dogs with diarrhea (21.8%) exhibited a higher CaKoV prevalence than the group without diarrhea (7.2%). Additionally, CaKoV was detected across all age groups, except for senior dogs (Table 1). The highest prevalence of CaKoV detection was observed in puppies (30.8%), with a significantly higher prevalence compared to juveniles, mature adults, and geriatric dogs, showing differences of (p = 0.0039; OR = 5.02; 95% CI: 11.6801–14.9694); (p = 0.0004; OR = 9.21; 95% CI: 2.7152–31.2595), and (p = 0.0284; OR = 9.8; 95% CI: 1.2737–75.5110), respectively.

**Table 1:** Prevalence of canine kobuvirus (CaKoV) detection in Vietnam and Thailand by age of investigated dogs.

Age groups (month)	Number of CaKoV-positive dogs/Total collected dogs (%)								
	Vietnam			Thailand			Overall		
	Non-diarrhea	Diarrhea	Total	Non-diarrhea	Diarrhea	Total	Non-diarrhea	Diarrhea	Total
Puppies (≤ 6)	3/15 (20%)	22/46 (47.8%)	25/61 (41%)	5/38 (13.2%)	7/21 (33.3%)	12/59 (20.3%)	8/53 (15.1%)	29/67 (43.3%)	37/120 (30.8%)
Juveniles (> 6–12)	1/15 (6.7%)	2/9 (22.2%)	3/24 (12.5%)	0/8 (0%)	1/17 (5.9%)	1/25 (4%)	1/23 (4.3%)	3/26 (11.5%)	4/49 (8.2%)
Young adults (> 12–24)	0/2 (0%)	4/4 (100%)	4/6 (66.7%)	0/9 (0%)	1/11 (9.1%)	1/20 (5%)	0/12 (0%)	4/14 (28.6%)	5/26 (19.2%)
Mature adults (> 24–72)	1/12 (8.3%)	0/9 (0%)	1/21 (4.7%)	1/21 (4.8%)	1/23 (4.3%)	2/44 (4.5%)	2/33 (6.1%)	1/32 (3.1%)	3/65 (4.6%)
Senior (> 72–132)	0/1 (0%)	–	0/1 (0%)	0/18 (0%)	0/25 (0%)	0/43 (0%)	0/19 (0%)	0/25 (0%)	0/44 (0%)
Geriatric (≥ 132)	–	–	–	1/13 (7.7%)	0/10 (0%)	1/23 (4.3%)	1/13 (7.7%)	0/10 (0%)	1/23 (4.3%)
Total	4/46 (8.7%)	28/67 (41.8%)	33/113 (28.3%)	7/107 (6.5%)	10/107 (9.3%)	17/214 (7.9%)	11/153 (7.2%)	38/174 (21.8%)	49/327 (15%)

–, no data available.

In addition to CaKoV, positive samples were concurrently identified with other common enteric viruses, including canine parvovirus (CPV), canine distemper virus (CDV), canine coronavirus (CCoV), and canine astrovirus (CaAstV). Details of these co-detection instances are outlined in Supplementary Table 2. The prevalence of single CaKoV detection was 21.2% in Vietnam and 52.9% in Thailand. Notably, co-detection of CaKoV-positive samples with three or four viruses mentioned above were observed exclusively in Vietnam and not in Thailand.

### Genetic characterization of the CaKoV in Vietnam and Thailand

The CDS of CaKoV from Vietnam (9 sequences) and Thailand (4 sequences) were successfully characterized and deposited in GenBank database under accession numbers PP320358-PP320370. The CaKoV genome spanned a length of 7571 nt, featuring a single ORF that encoded 2444 aa. The ORF initiated with the methionine codon (Met) and ended with the alanine codon (Ala). Details outlining the structure of the CaKoV genome, as identified in this study, are presented in Table 2. Additionally, information about the RS samples from dogs that were used to assemble the complete CaKoV genome is provided (Supplementary Table 3).

**Table 2:** Structure and length of the complete coding sequences (CDS) of canine kobuvirus (CaKoV) in Vietnam and Thailand.

		Genome structure CaKoV	Length (nt)
		5′UTRs	113
		Leader protein (L)	513
Structural protein region	P1 region (2649 nt)	VP0	1146
		VP3	669
		VP1	834
Non-structural protein region	P2 region (1833 nt)	2A	333
		2B	495
		2C	1005
	P3 region (2340 nt)	3A	282
		3B	81
		3C	1170
		3D	807
		3′UTRs	123

**Figure 1:**
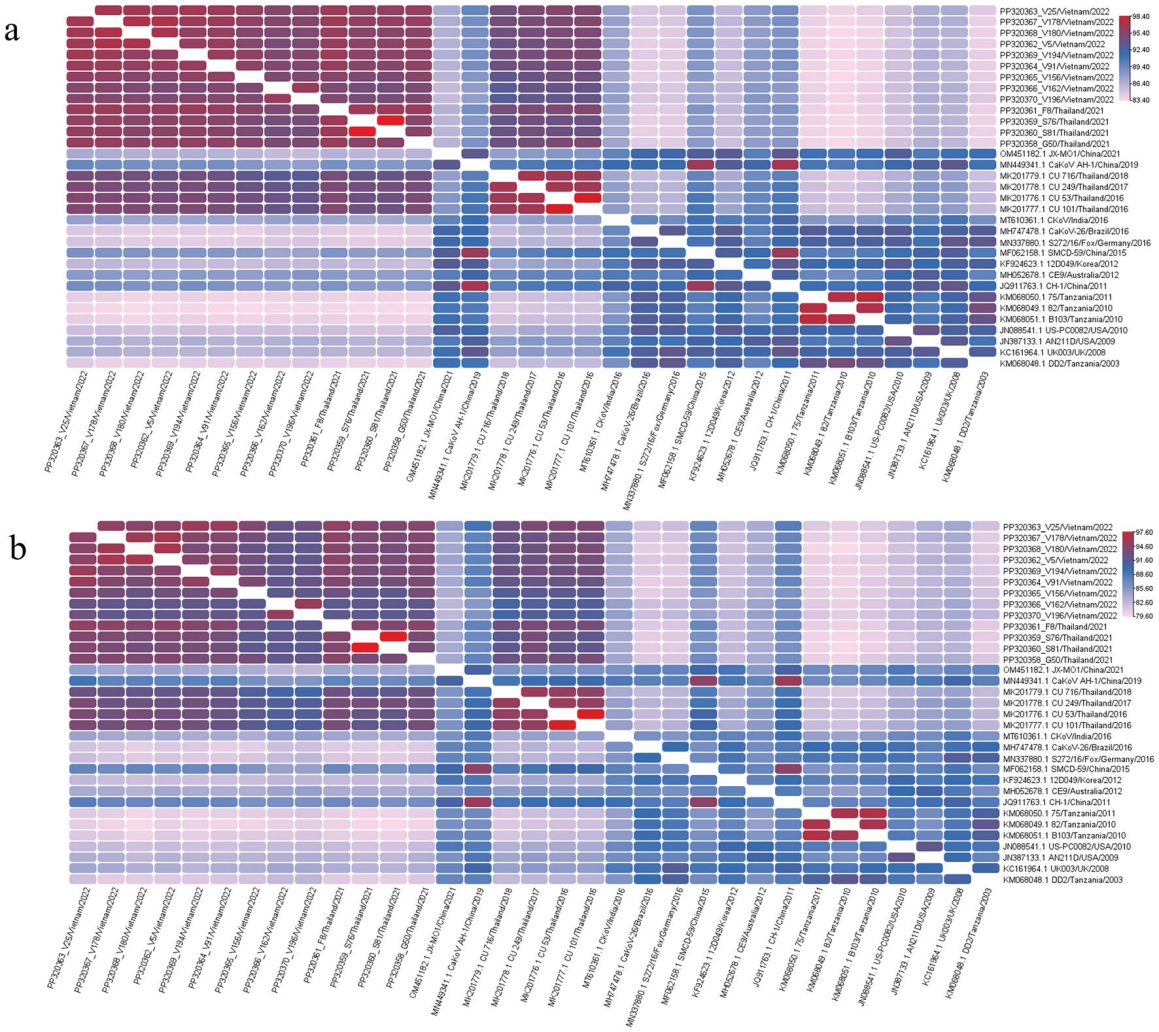
Heatmap of the nucleotide and amino acid similarity of complete coding sequences of canine kobuvirus (CaKoV) among isolates from Vietnam and Thailand, along with reference strains. (**a**) The similarity of nucleotide sequences; (**b**) the similarity of amino acid sequences.

The nucleotide and amino acid similarities between the CaKoV sequences from Vietnam and those from Thailand in this study were compared with strains previously available in the GenBank database, originating from diverse countries such as Thailand, China, Germany, India, South Korea, the UK, Australia, Tanzania, Brazil, and the United States (Fig. 1, Supplementary Table 4, Supplementary Table 9). Within the CDS of Vietnamese CaKoV sequences, a similarity of 95–97.3% in nt and 92–96.4% in aa was observed. The four CDS of Thai CaKoV sequences exhibited a similarity of 96.3–99.9% in nt and 93.6–99.9% in aa. When comparing CDS of CaKoV obtained from Vietnam and Thailand, they displayed similarities of 94.6%-96.5% in nt and 91.9%-95% in aa. Remarkably, they showed the highest nt similarity with the Thai strains from the previous study (MK201176-79) and the Chinese strain (MN449341), while exhibiting the lowest nt similarity to Tanzanian strains (KM068048-51) (Fig. 1, Supplementary Table 4). As expected, the VP1 gene was the most variable region for CaKoV, with nt similarities ranging from 81.6 to 96.5% and aa similarities ranging from 81.1 to 98.5% (Supplementary Table 4). Notably, no recombination events were identified in the CaKoV strains obtained in this study.

Ten putative cleavage sites within the CaKoV genomic structure were predicted (Supplementary Table 5). In particular, the putative cleavage sites of the polyprotein mostly contained amino acid residues Q/G, except Q/H, Y/V and Q/A for the cleavage sites between VP0/VP3, VP1/2A, and 3A/3B, respectively. The putative cleavage site between VP3/VP1 was Q/A, conserved in almost all strains, except for wild carnivore strains (the spotted hyena and the side-striped jackals) in Tanzania (KM068049.1 and KM068051.1), where it was replaced by Q/T. A putative proline-rich region was identified at aa position 228-240 of the VP1 gene, with the motif ^228^PRAPPPLPPLPTP^240^ conserved in almost all strains, except for strains JN387133.1/USA (^228^PRAPP-LPPLPTP^240^), KM068049.1/Tanzania, KM068050.1/Tanzania, JN088541.1/USA (^228^CPVPPPLPPLPTP^240^), MH747478.1/Brazil (^228^HGAPPPLPPLPTP^240^), (Supplementary Table 6). Amino acid positions 60, 65, 67, 69, 150, 151, 153, 201, 204, 205, 210, 213 were conserved in almost Vietnamese and Thailand CaKoV strains (in this study), Thailand CaKoV strains (in a previous study), and Chinese strains. However, for two strains PP320366_V162/Vietnam/2022 and PP320370_V196/Vietnam/2022, the aa positions mentioned above were different and not conserved when compared to the remaining Vietnam and Thailand strains. Interestingly, these positions were conserved with Tanzanian and USA strains (Supplementary Table 6).

### Phylogeny of the Vietnamese and Thai CaKoV

**Figure 2:**
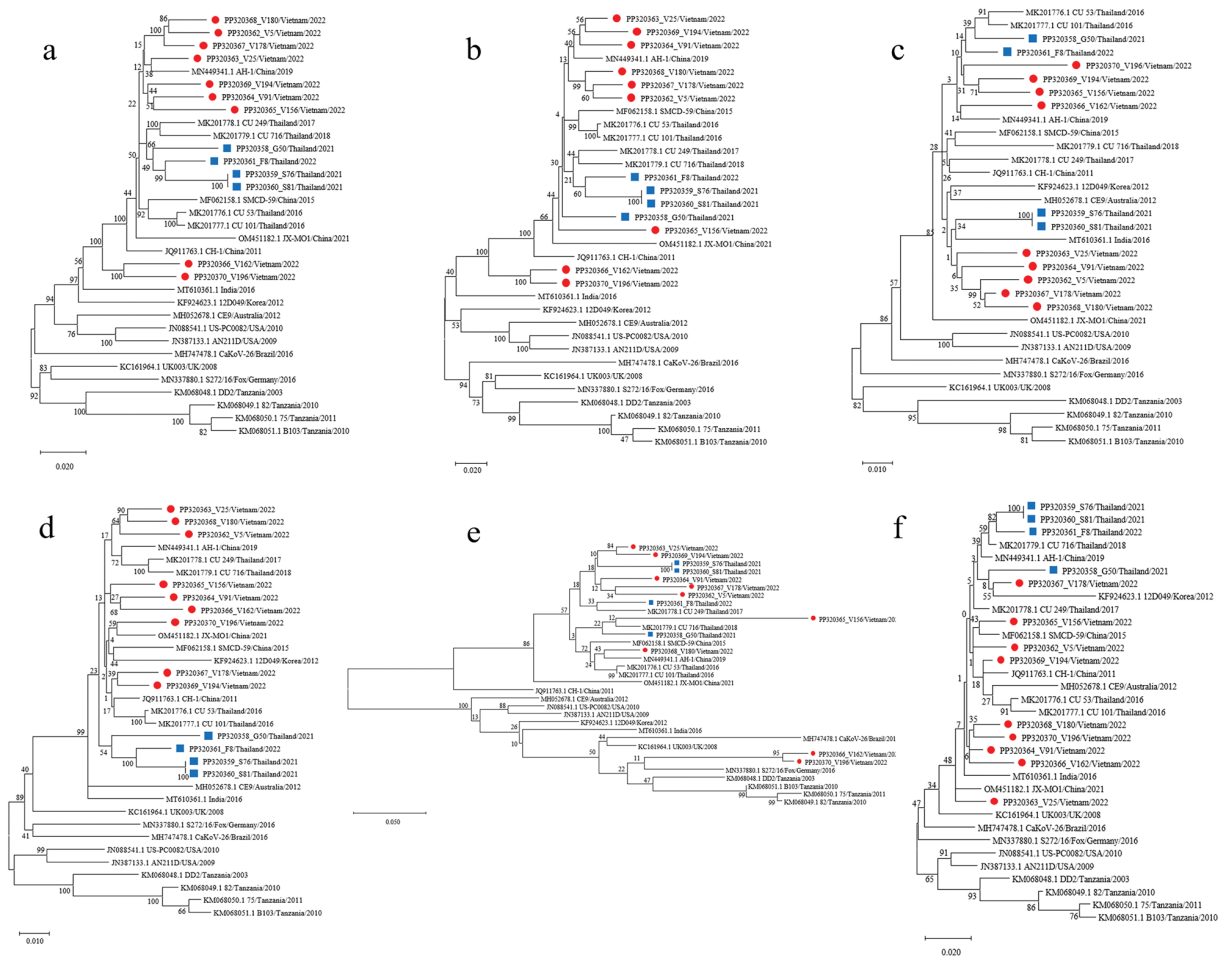
Phylogenetic trees of canine kobuvirus (CaKoV) for various regions: (**a**) the complete coding sequences (CDSs), (**b**) P1 region, (**c**) P2 region, (**d**) P3 region, (**e**) VP1 gene, and (**f**) 3D gene. Trees of CDSs, P1, P2, and P3 region were constructed using MEGA v10.0 with the neighbor-joining algorithm employing the general time-reversible model. For VP1 and 3D genes, the trees were constructed with the Hasegawa–Kishono–Yano method, incorporating a gamma distribution and invariable sites (G + I) for complete coding sequences, P1, P2, P3 region, and the 3D gene. The VP1 gene tree employed a gamma distribution (G). All trees underwent 1,000 replications of bootstrap analysis. Vietnamese CaKoV isolates are marked with red circle, and Thai CaKoV isolates are noted by blue square.

The phylogenetic analysis, based on the nucleotide sequences of the CDS, P1–P2–P3 region, VP, and 3D genes of nine CaKoV Vietnamese strains and four CaKoV Thai strains, is presented in Fig. 2. The tree based on the CDS indicated a close genetic relationship between Vietnam and Thailand strains, showing genetic affinities with Chinese strains. Interestingly, two Vietnamese strains (PP320366_V162/Vietnam/2022 and PP320370_V196/Vietnam/2022) formed a distinct subgroup, sharing genetic characteristics with both Chinese and Thai strains. The phylogenetic tree based on the P1 region exhibited similar characteristics to the CDS tree; however, the PP320365_V156/Vietnam/2022 strain formed a separate clade. Furthermore, the phylogenetic tree based on the VP1 gene, known for its high variability and belonging to the P1 region, suggested that the two CaKoV strains from Vietnam (PP320366_V162/Vietnam/2022 and PP320370_V196/Vietnam/2022) were closely related to strains MN337880 (isolated from foxes in Germany), and KM068049-068051 (isolated from wild carnivores in Tanzania), rather than strains isolated from domestic dogs in Asia and the Americas. Examining the P2 and P3 regions, the phylogenetic tree revealed that the Vietnamese and Thai strains in this study tended to cluster together, sharing genetic characteristics with strains from China, India, Australia, and previous Thai strains. They were separated from strains in Europe, the USA, Brazil, and Tanzania. For the 3D gene of CaKoV, belonging to the P2 region, the phylogenetic tree displayed less genetic variation, with the strains in this study forming a cohesive subgroup.

### B-cell epitope prediction for Vietnamese and Thai CaKoV

**Figure 3:**
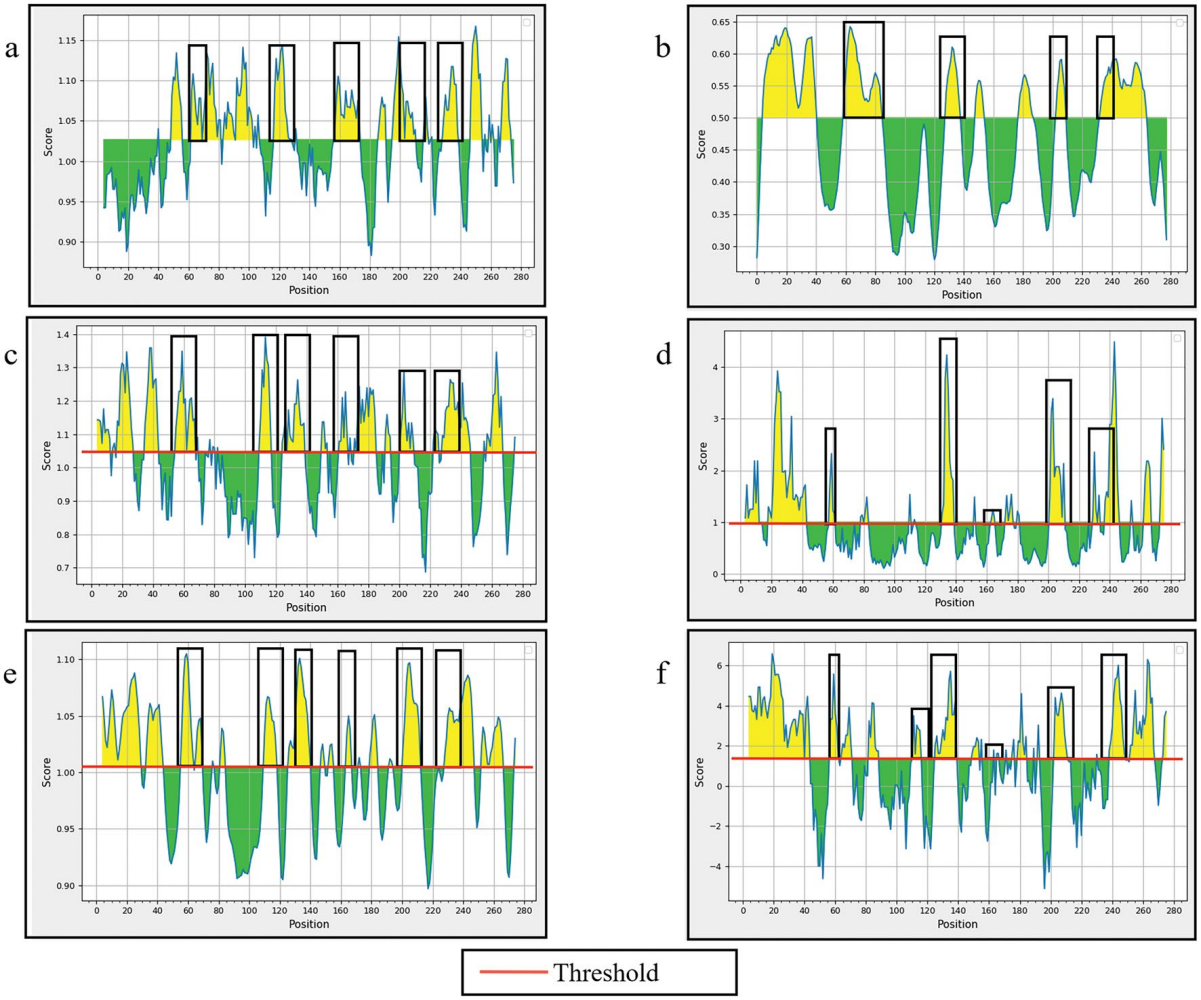
The physicochemical properties of canine kobuvirus (CaKoV) VP1 protein were evaluated using various methods: (**a**) Kolaskar & Tongaonkar antigenicity, (**b**) BepiPred linear epitope 2.0, (**c**) Chou & Fasman Beta-turn prediction, (**d**) Emini surface accessibility, (**e**) Karplus & Schulz flexibility prediction, and (**f**) Parker hydrophilicity. The Y-axis represents the corresponding score for each residue, while the X-axis corresponds to the residue positions in the sequence.

The VP1 capsid gene of CaKoV was employed for the prediction of both continuous and discontinuous B-cell epitopes. Multiple methods were utilized for predicting continuous B-cell epitopes, and the outcomes from each method were visualized through distinct charts and tables (refer to Table 3 and Fig. 3).

**Table 3:** Analysis of B-cell epitopes of the VP1 gene of canine kobuvirus (CaKoV) sequences obtained in this study using the Kolaskar and Tongaonkar antigenicity method.

No.	Start	End	Peptide^a^	Conserved amino acid/ total amino acid
1	49	55	FYRLLPL	7/7
2	62	68	SIPVPDG	4/7
3	70	81	VAQLPLDPLHWQ	11/12
4	83	90	SADVAGLT	8/8
5	92	102	MLSCFTYIAAD	11/11
6	117	128	TSLLIAYAPPGA	10/12
7	156	173	LISFSIPYTSPLSAIPTS	18/18
8	195	210	TLLFLPSYPQQDVQPQ	10/16
9	228	238	PRAPPPLPPLP	11/11
10	247	254	SVAVVKQG	8/8
11	267	273	RVYIVRA	7/7

^a^The diversity of amino acid residues within the predicted epitopes from the deduced amino acid sequences of PP320363_V25/Vietnam/2022 (VP1 gene) was analyzed Vietnamese and Thai CaKoV sequences.

Residues with scores above the threshold (depicted by the red line on the chart) were considered indicative of having a higher likelihood of being potential epitopes. Additionally, the overlapping aa regions from all plots, based on the results of all prediction methods, served as the basis for suggesting potential continuous B-cell epitopes (Fig. 3).

**Figure 4:**
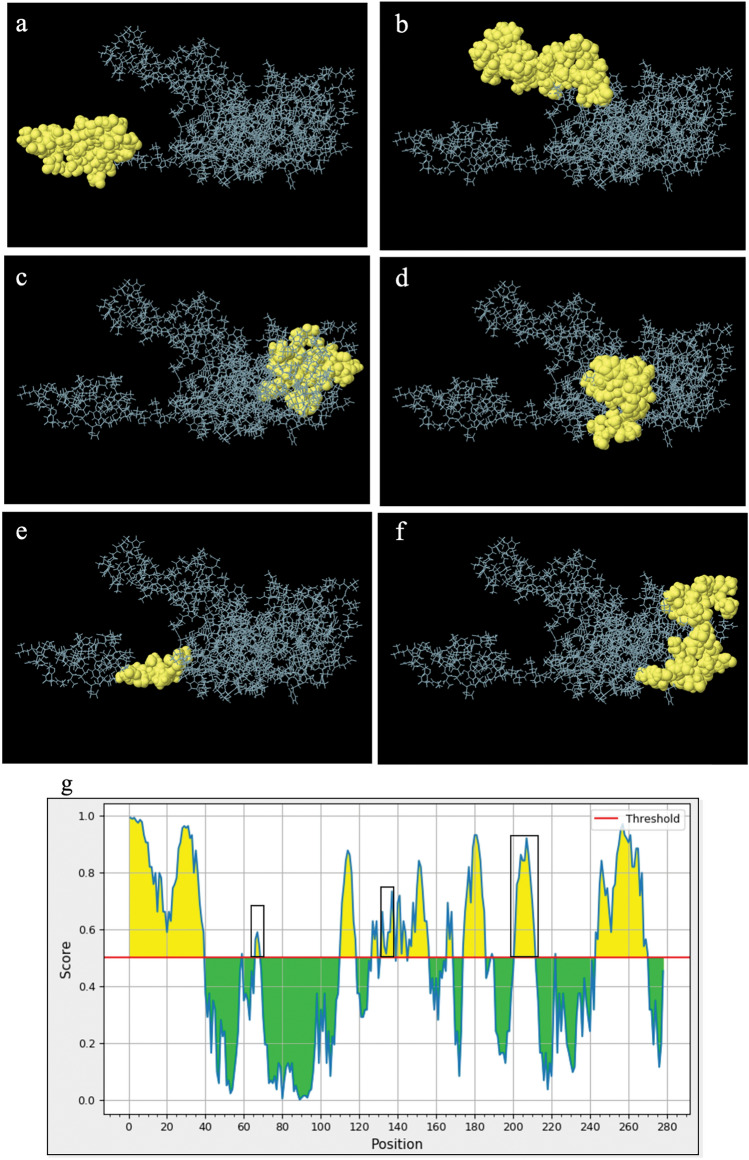
The presentation of B-cell epitopes derived from the VP1 capsid of canine kobuvirus (CaKoV) employing ElliPro server: (**a–f**) Predicted discontinuous B-cell epitopes, and (**g**) Predicted B-cell linear structure.

The ElliPro tool identified six discontinuous epitopes from the CaKoV VP1 gene capsid structure (Fig. 4). These epitopes varied in size from 8 to 36 residues, with scores ranging from 0.608 to 0.848. Information on the residues associated with discontinuous epitopes, including the quantity, positions, and scores, were detailed in Supplementary Table 8. Notably, the ElliPro predictions for discontinuous B-cell epitopes, based on the CaKoV VP1 region, overlapped with the linear B-cell epitopes. Furthermore, the Ellipro chart depicting continuous B-cell epitopes (Fig. 4g) revealed yellow areas, aligning with antigenic characteristics as indicated by the linear epitope predictions from the BepiPred linear epitope 2.0 method (Fig. 3b).

### Selection pressure for Vietnamese and Thai CaKoV

The analysis of the dN and dS ratios derived from the CaKoV VP1 gene alignment was conducted using the MEME and SLAC methods. Both analyses suggested that the VP1 gene of CaKoV from Vietnam and Thailand underwent negative selection pressure (with dN/dS < 1), indicating a tendency towards purifying or stabilizing selection. However, it is noteworthy that potential positive selection sites were also identified, implying regions where positive selection might be occurring (Table 4).

**Table 4:** The selection sites of Vietnamese and Thai canine kobuvirus (CaKoV) based on VP1 region.

CaKoV VP1 gene	Selection pressure	MEME^†^	SLAC	FEL	FUBAR^‡^
Vietnamese strains	Positive selection	2	0	0	1
	Negative selection	–	28	108	107
	Overall dN/dS	0.0549	0.0707	N/A	N/A
Thai strains	Positive selection	4	0	0	0
	Negative selection	–	19	74	80
	Overall dN/dS	0.0488	0.0628	N/A	N/A

^†^MEME: positive selection for Vietnamese strains at sites 204th and 206th; for Thai strains at sites 69th, 82nd, 138th and 206th.

^‡^FUBAR: positive selection for Vietnamese strains at site 206th.

The analysis of selective pressure on the VP1 region of CaKoV revealed specific codon positions that were under positive selection. For Vietnamese CaKoV, the 204th and 206th codon positions were identified as positive selected, with the 206th codon being detected by both MEME and FUBAR methods, while the 204th codon was identified only by the FUBAR method. For Thai CaKoV, four codon positions (69th, 82nd, 138th, and 206th) were found to be positively selected, but they were only detected by the MEME method. The analysis also explored the relationship between the impact of selective pressure and B-cell epitopes. In both Vietnamese and Thai CaKoV, there was a positively selected position at the 206th codon, which corresponds to positions within predicted linear and conformational B-cell epitopes; the Thai CaKoV strain exhibited the additional presence of two positively selected positions, 69th and 138th codons. This suggests a potential interplay between selective pressure and the regions of the virus that are recognized by the immune system.

## Discussion

This study revealed a prevalence of CaKoV in Vietnam and provided an update status of CaKoV in Thailand, showing a lower prevalence compared to a previous study^28^. Our investigation found that the highest detection rate of CaKoV was in dogs under 6 months old, aligning with similar patterns observed in previous reports, where the highest prevalence occurred in dogs under 4 months old in Japan (Soma et al.^27^) and 1 year old in Thailand^28^. Notably, human studies have also reported a higher incidence of Human Aichi Virus (AiV) infection in children^35–37^. In addition to affecting young dogs, CaKoV was found across various age groups in this study, consistent with findings in both humans^38^ and dogs^28^. However, a study in Japan reported that CaKoV was not detected in dogs over 5 months old^27^. These variations suggest that the prevalence of CaKoV may differ between countries, influenced by factors such as sample sizes and the specific age groups sampled.

The detection of CaKoV in both diarrhea and non-diarrhea dogs aligns with similar findings in studies from China^2,23^, Korea^26^, Japan^27^. The CaKoV prevalence in dogs with diarrhea tends to be higher than in dogs without diarrhea, but due to the small sample size in this study, further research is needed to clarify this point. Moreover, in dogs with diarrhea, CaKoV was identified either as a single detection or in co-detection with other common enteric viruses. This pattern is consistent with previous research where CaKoV was found either as a sole detection^2,19,22,26,27^ or in co- detection with viruses such as CPV2, rotavirus, CCoV, CDV, CaAstV, and Canine bocavirus^2,19,20,22,25–27,39^, as well as bacteria or parasites like *Campylobacter upsaliensis*, *Toxocara canis*, *Toxocara leonina*^20^ and giardiasis^32^. Similarly, studies on AiV have shown a high rate of single AiV infection, suggesting its potential role in gastrointestinal tract diseases in humans^38^. However, the true pathogenic role of CaKoV remains uncertain. It is unclear whether CaKoV can independently cause disease or if it collaborates with other enteric pathogens to induce gastroenteritis in dogs^40^. A global study suggests that CaKoV might play a sporadic pathogenic role in specific dog populations or interact with other enteric pathogens^29^. Therefore, additional observational studies or experimental infections are crucial to understanding the pathogenic mechanism of CaKoV in the gastrointestinal tract and its interactions with other pathogens^4^. However, the inability to cultivate CaKoV poses a significant challenge in the pathobiological research of this virus^20,26^.

In the comparison of CaKoV structure between this study and AiV, it was noted that the proline-rich region at aa position 50-63 of the VP0 gene of CaKoV was absent. Only the proline-rich region at aa position 228-240 (P^228^XPPPPXPPXPXP^240^), which belong to the VP1 gene, showed similarity between CaKoV and AiV^41^. This distinctive proline-rich region plays a crucial role in mediating host-virus interactions, precise localization of adhesion, infectivity, and viral pathogenesis^42,43^. In the phylogenetic analysis, the results of this study revealed a significant tendency for strains from the same country to cluster together, consistent with previous reports^19,29,34,39^. The phylogenetic tree of CDS indicated that the sequences from China, Vietnam and Thailand formed an independent group, separated from sequences found in Africa, the United States, and Europe^28,39^. However, two Vietnamese CaKoV strains (PP320366_V162/Vietnam/2022, PP320370_V196/Vietnam/2022) showed a tendency to form a new monophyletic subgroup. Moreover, the VP1 gene of strains PP320366_V162/Vietnam/2022 and PP320370_V196/Vietnam/2022 exhibited genetic heterogeneity compared to other Vietnamese CaKoV strains. Importantly, recombination events were not recorded for the Vietnamese and Thai CaKoV strains in this study. These findings suggest that strains PP320366_V162/Vietnam/2022 and PP320370_V196/Vietnam/2022 may have regional characteristics or may be ancestral strains. However, the availability of complete CaKoV genome sequences was relatively limited, and increasing the number of CaKoV CDSs in various countries would help clarify this issue.

B-cell epitopes, or antigenic determinants, are distinct clusters of amino acids on an antigen that are recognized by secreted antibodies or B-cell receptors, inducing cellular or humoral immune responses^44,45^. The analyis and prediction of B-cell epitopes were conducted using IEDB resources, focusing on the VP1 gene region of kobuvirus. This region is essential for receptor interaction, exhibits high immunogenicity among picornavirus capsid proteins, and experiences significant selective pressure^7,15,46–48^. The impact of selective pressure on the host immune response to CaKoV was explored. The analysis revealed that negative selection predominantly influenced the evolutionary dynamics of the VP1 gene region of CaKoV in Vietnam and Thailand. This suggests that external selection pressure may not be closely linked to the presence of mutations. Negative selection reflects an inclination to maintain the overall antigenic stability of CaKoV, indicating a low evolutionary rate and a delicate balance between virus-host interaction and survival ability^49–53^. However, potential positive selection positions in the VP1 gene were still identified. This suggests that selective pressure tends to alter the B-cell epitope of CaKoV, making it less conserved. Consequently, the host's immune response to CaKoV may undergo changes, potentially leading to immune evasion.

In summary, this study found that CaKoV was prevalent in both diarrheic and non-dirrheric dogs of all ages, with the highest prevalence observed in dogs under 6 months old. CaKoV was not only detected as a single infection but also in co-detection with other common gastrointestinal viral pathogens. The phylogenetic tree and genomic characteristics analyses revealed a close relationship between Vietnamese and Thai CaKoV strains, and they were also closely related to Chinese strains. Notably, Vietnamese strains formed a unique sub-branch. Selective pressure analysis showed that negative selection was the predominant force shaping the evolution of Vietnamese and Thai CaKoVs. However, potential positive selection sites were identified within the VP1 gene, particularly in regions associated with predicted B-cell epitopes. This suggests that while there is overall pressure to maintain stability, certain regions of the virus might be evolving under positive selection, possibly impacting host immune responses. The need for further research to elucidate the pathogenic mechanisms and immune responses associated with CaKoV infections in dogs.

## Materials and methods

### Sample collection and RNA extraction

A total of 327 RS samples were collected from pet dogs visiting veterinary clinics in Vietnam and Thailand between August 2021 and August 2022. This comprised 113 samples from Ho Chi Minh City, Vietnam (46 from healthy dogs and 67 from GID dogs) and 214 samples from Bangkok and Nakhon Si Thammarat Province, Thailand (107 from healthy dogs and 107 from GID dogs). The RS samples were randomly collected from both healthy dogs and those with diarrhea. However, dogs with clinical manifestations of gastrointestinal issues due to food poisoning, hepatobiliary disease, or those vaccinated within the last 4 weeks were excluded from the study. Information about the sampled dogs, including age, breed, sex, and vaccination status, was recorded using a questionnaire. To analyze age-related risk factors associated with CaKoV detection, all dogs were categorized into six age groups, adjusted based on established age classifications for dogs^54^.

The animal sampling and research protocols for this study were reviewed and approved by the Institutional Animal Care and Use Committee (IACUC) (No. 2231006) and the Institutional Biosafety Committee (IBC) (No. 2131019) of Chulalongkorn University (Bangkok, Thailand). Approval was also obtained from the Animal Ethics Committee (AEC) (No. NLU-220217) of Nong Lam University (Ho Chi Minh City, Vietnam). All procedures were performed in accordance with the relevant guidelines and regulations. The authors complied with the ARRIVE guidelines. Written informed consent was obtained from the owners for the participation of their animals in this study.

The RS sampling procedure involved inserting a sterile disposable cotton swab (Puritan, Guilford, USA) into the dog’s rectum, followed by immersion in a 1.5 mL Eppendorf tubes containing 0.7 mL of 1% (v/v) sterile phosphate-buffered salt (PBS) at pH 7.4. The samples were stored at − 80 °C until the extraction step. Viral RNA extraction was carried out using the Nucleic Acid Extraction Kit II (Geneaid, Ltd., Taipei, Taiwan) according to the manufacturer's protocol. Subsequently, the extracted RNA samples were quantified and assessed for quality using a Nanodrop® Lite spectrophotometer (Thermo Fisher Scientific Inc., Waltham, MA, USA) at the absorbance ratio A260/A280 and stored at − 80 °C until further use.

### CaKoV detection

The extracted RNA samples underwent screening for the presence of CaKoV using the reverse transcription-polymerase chain reaction (RT-PCR) method with specific primers. These primers were self-designed to target the 3D gene region of CaKoV, referencing the available CaKoV sequence JQ911763 (Supplementary Table S1). The total volume was adjusted to 25 μl for the QIAGEN® One-Step RT-PCR Kit (Qiagen GmbH, Hilden, Germany), including 5 μl of QIAGEN 5 × buffer, 1 μl of 10 mM dNTP Mix, 2 μl of 0.6 μM forward and reverse primers, 1 µl of enzyme mixture, 3 µl of extracted RNA, and 11 µl of distilled water.

The thermal cycling conditions were conducted on a PCR thermocycler (SensoQuest GmbH, Göttingen, Germany), involving a complementary DNA (cDNA) synthesis step at 50 °C for 30 min, followed by inactivation of reverse transcription (RT) enzyme and initial PCR activation step at 95 °C for 15 min. Subsequently, 40 cycles of denaturation at 95 °C for 30 s, annealing at 57 °C for 30 s, and extension at 72 °C for 1 min were carried out, followed by a final extension step at 72 °C for 10 min. The positive control for CaKoV was synthesized using GeneArt™ Strings™ DNA fragments based on the 3D region of strain JQ911763 (Thermo Fisher Scientific GmbH, Darmstadt, Germany). A no-template control (NTC) was employed as a negative control. PCR products were visualized using the QIAxcel® DNA Screening Kit (Qiagen GmbH, Hilden, Germany) and the Qiaxcel® high-resolution capillary electrophoresis device (Qiagen GmbH, Hilden, Germany). Installation and analysis procedures were followed a previous report protocol^55^. The presence of the 438 bp amplicon product was considered as CaKoV positive. Subsequently, the PCR products were sent for genetic sequencing using a NGS-based method (Celemics, Inc., Seoul, Korea) to double-confirm the presence of CaKoV. The derived nucleotide sequences were analyzed and aligned with previously described CaKoV sequences depositing in the GenBank database using BLASTn analysis. CaKoV-positive samples were also tested for co-detection with other common canine enteric viruses by PCRs, including CPV^56^, CDV^57^, CCoV^58^, and CaAstV^59^.

### CaKoV whole genome characterization

CaKoV-positive samples, initially identified by CaKoV-PCR screening, underwent further investigation for full-length genome analysis using multiple PCR assays. Seven primer pairs, self-designed based on nucleotide alignments from previously described CaKoV sequences available in the GenBank database (Supplementary Table 1), were employed to amplify the full-length CaKoV genome. The initial step involved the construction of cDNA from the extracted RNA samples, using the Omniscript® Reverse Transcription Kit (Qiagen GmbH, Hilden, Germany). For cDNA synthesis, the final volume was 20 μl, comprising 2 μl of 10 × RT buffer, 2 μl of 5 mM dNTP mixture, 1 μl of 10 μM random primer (Promega, Madison, Wisconsin, USA), 1 μl of RNase-free water, 0.75 μl of 1 × buffer, 0.25 μl of RNase inhibitor (10 units/μl), 1 μl of Omniscript reverse transcriptase, and 12 μl of extracted RNA sample.

Subsequently, the PCR reaction was performed with a final volume of 25 μl, including 3 μl of cDNA, 1 μl of 1 μM for each forward and reverse primers, 12.5 μl of GoTaq® Green Master Mix (Promega, Madison, Wisconsin, USA), and 7.5 μl of distilled water. The thermal cycling conditions included an initial denaturation at 95 °C for 5 min, followed by 40 cycles of 95 °C for 1 min, 55–58 °C for 1 min, and 72 °C for 1 min, and then a final extension at 72 °C for 10 min. PCR products were visualized using the QIAxcel® DNA Screening Kit, as mentioned above. Positive amplicons were then subjected to genomic sequencing according to the protocol described above. Subsequently, the obtained gene sequences were aligned and assembled using the BioEdit software package version 7.2 with the ClustalW function.

### Phylogenetic and genetic analyses

Genetic analysis involved comparing the similarity of the CaKoV nucleotide sequence obtained from this study with the CaKoV sequences available in the database. Phylogenetic trees were constructed based on the complete coding sequence (CDS), P1–P2–P3 regions, and two genes, VP1 and 3D of CaKoV. The MEGA software package version 10.0 was used for this purpose. The software utilized the Find Best DNA/Protein Models (ML) option to select the substitution model for the constructed phylogram. The maximum likelihood (ML) method was applied, utilizing the General Time Reversible model (GTR) for CDS, P1, P2, P3 region, and the Hasegawa–Kishono–Yano (HKY) for VP1 and 3D genes. Gamma distribution and invariable sites (G + I) were considered for CDS, P1, P2, P3 region, and 3D gene, while a gamma distribution (G) was used for VP1 gene. The analysis included 1000 bootstrap replicates to assess the robustness of the phylogenetic relationships. To further evaluate the relationship between the CaKoV strains obtained in this study and other strains, deduced amino acid sequences of CaKoV were aligned and compared using the BioEdit software package, version 7.2. Additionally, TBtools was employed to create a heatmap^60^ via https://github.com/CJ-Chen/TBtools/releases.

### Recombination analysis

All CaKoV strains obtained in Vietnam and Thailand underwent screening for potential genetic recombination events using the Recombination Detection Program version 4.0 (RDP4) software package. This comprehensive package incorporates seven different integrated recombination detection algorithms, namely RDP, GeneConv, Chimera, MaxChi, SiScan, 3Seq, and BootScan. To ensure reliability and consistency in results, potential recombinant sequences were considered only when positive results were obtained in at least 4 out of 7 methods with a *p*-value of ≤ 0.01^61,62^. This approach minimizes the risk of detecting false-positive signals. Subsequently, to visualize recombination breakpoints, potentially recombinant sequences were subjected to detailed analysis using SimPlot software version Beta 4.94. This involved employing two methods: similarity plot analysis and Bootscan analysis. The procedures for implementing this analytical method adhered to a previously published procedure^55^.

### B-cell epitope prediction

The nucleotide sequences of VP1 capsid gene of CaKoV capsids were *in-silico* translated into amino acid sequences, and these sequences underwent analysis using the B-Cell Epitope Online Prediction Tool: “Analysis Resources and immune epitope database” (IEDB-AR) version 2.27 available at http://www.iedb.org/^63^. The tool was employed to identify both continuous B-cell epitopes (linear B-cell epitopes) and discontinuous B-cell epitopes (3D structure of the antigen) derived from the primary amino acid sequence.

For the prediction of continuous B-cell epitopes, the Kolaskar & Tongaonkar antigenicity method was used^64^. Additionally, to enhance the accuracy of predicting continuous B-cell epitopes, the correlation of all given single physicochemical properties was evaluated using five other propensity scales: (1) BepiPred 2.0 linear epitopes (for predicting specific regions in the protein that bind to the B-cell receptor)^65^, (2) Parker hydrophilicity (highlighting hydrophilic epitope regions)^66^, (3) Emini surface accessibility (indicating epitope surface accessibility)^67^, (4) Chou & Fasman Beta turn prediction (determining beta-turn regions as epitopes)^68^, and (5) Karplus & Schulz mobility prediction (assessing the mobility of protein segments)^69^. The setting parameters of all above methods, including the threshold, average score, minimum score, and maximum score of each method, are shown in Supplementary Table 7.

For predicting discontinuous B-cell epitopes, the online Ellipro tool of IEDB-AR server v2.27 (http://tools.iedb.org/ellipro/) was used^70^. In this approach, the minimum score and maximum distance (Angstrom) were set at 0.5 and 6, respectively.

### Selection pressure analysis

The assessment of non-neutral nucleotide substitution involved calculating the ratio between synonymous substitutions (dS) and non-synonymous substitutions (dN). This evaluation was conducted through phylogenetic reconstruction using a ML model with general reversible nucleotide substitutions and performed using the Datamonkey web server (http://www.datamonkey. org). The Bayes factor was set at 50 to evaluate the dN and dS rates in each specific codon^56,61^. Positive selection (representing adaptive molecular evolution), neutral mutation, and negative selection (representing purifying selection) were defined as dN/dS > 1, dN/dS = 1, and dN/dS < 1, respectively. Sites under selection pressure were predicted using the HyPhy software package, available on the Datamonkey web server (http://www.datamonkey.org/), which includes four methods: mixed effects (MEME), Single Likelihood Ancestry Counting (SLAC), Fixed Effects Likelihood (FEL), and Fast Unconstrained Bayesian Approximation for Choice Inference (FUBAR). To confirm sites with positive selection pressure for each method, a *p*-value threshold < 0.1 for SLAC, FEL and MEME was established, while for FUBAR the posterior probability exceeded 0, 9 was used. Sites showing positive selection were identified when consistent results were obtained from a minimum of two of these methods. The CaKoV Thai strains from a previous study in Thailand (MK201776-79)^28^ were also included in the analysis of selective pressure, in conjunction with the CaKoV Thai strains from the current study.

### Statistical analysis

Statistical analysis was performed using SAS® Studio software 3.81 (SAS® OnDemand for Academics) (https://www.sas.com/el_gr/software/on-demand-for-academics.html) (© 2012-2020 SAS Institute Inc., Cary, NC, USA). The correlation between the presence of CaKoV and age group was analyzed using Pearson's chi-square test or Fisher’s exact test (depending on the population size for each variable). A *p*-value < 0.05 was considered statistically significant to evaluate the relationship. Additionally, odds ratios (ORs) were calculated to quantify the strength of association between each factor and the presence of CaKoV.

### Supplementary Information

Supplementary Tables.

## Data Availability

The data used to support the findings of this study have been deposited in NCBI GenBank under accession numbers PP320358-PP320370.
