# Supplementary material for: Evolutionary dynamics of canine kobuvirus in Vietnam and Thailand reveal the evidence of viral ability to evade host immunity
Source: Sci Rep. 2024 May 27;14:12037. doi: 10.1038/s41598-024-62833-2 (PMC11130191; doi:10.1038/s41598-024-62833-2)
Supplement: Supplementary file 1 — Supplementary Tables. [file 41598_2024_62833_MOESM1_ESM.pdf]

Supplementary Material

Evolutionary dynamics of canine kobuvirus in Vietnam and Thailand reveal the evidence of viral ability to evade host immunity.

Tin Van Nguyen[1,2,3], Tanit Kasantikul[4], Chutchai Piewbang[3,5,*] and Somporn Techangamsuwan[3,5,*]

[1]The International Graduate Program of Veterinary Science and Technology (VST), Faculty of Veterinary Science, Chulalongkorn University, Bangkok, Thailand.

[2]Faculty of Animal Science and Veterinary Medicine, Nong Lam University, Ho Chi Minh City, Vietnam.

[3]Animal Virome and Diagnostic Development Research Unit, Faculty of Veterinary Science, Chulalongkorn University, Bangkok, Thailand.

[4]Veterinary Diagnostic Laboratory, Department of Pathobiology and Diagnostic Investigation, College of Veterinary Medicine, Michigan State University, East Lansing, MI, USA.

[5]Department of Pathology, Faculty of Veterinary Science, Chulalongkorn University, Bangkok, Thailand.

Supplementary Table 1 List of primers used for detection[a] and amplification[b] the 7 regions of canine kobuvirus (CaKoV) genome.

Direction	Sequence (5'-3')	Genome position	Product size (bp)
**Primers for CaKoV detection**			
Forward	CATCCAYTCCATCCGCCATT	6917 - 7354	438
Reverse	AAATCTCCRTCACGCARCCA		
**Primers for amplification the 7 regions of CaKoV genome**			
Forward	GTGTCCAYTCTAATACCCCGAG	625 - 2030	1406
Reverse	GGGTRTTGTACATGGCAGC		
Forward	CACGGTCACYTGGGARCA	1087 - 3038	1232
Reverse	GGACGTTRAAGGAYTCGCC		
Forward	GTGGACATCTTCAAYCCTGAT	2894 - 4163	1270
Reverse	CCAATGAGAAGCCGGTGTT		
Forward	TGTCTACATCGTYCGTGCAG	3877 - 5289	1413
Reverse	ATGTCGTCGATGAAGTGGAC		
Forward	CATGATGCATTACTCGGATTCC	4894 - 6199	1306
Reverse	GTAGAATCCRGACATGACRGC		
Forward	CTGGAAACCATAACCAACACC	5876 - 7134	1259
Reverse	TAATCCGGATCYTCAAGYGTG		
Forward	CGACACCCAACTCTTYCTCA	6832 - 8210	1379
Reverse	TACRACCATGGCTTAGGGG		

[a] Primers were designed based on the available CaKoV sequence JQ911763.

[b] Primers were designed based on the available CaKoV sequence MK201777.

Supplementary Table 2 Co-detection of canine kobuvirus (CaKoV) with other common enteric viruses.

**Co-detection scenario**[a]	**Vietnam (%)**	**Thailand (%)**
Single CaKoV detection	7/33 (21.2%)	9/17 (52.9%)
Co-detection with CPV	9/33 (27.3%)	0/17 (0%)
Co-detection with CDV	0/33 (0%)	0/17 (0%)
Co-detection with CCoV	2/33 (6.06%)	2/17 (11.8%)
Co-detection with CaAstV	4/33 (12.1%)	6/17 (35.3%)
Co-detection with CPV, CCoV	0/33 (0%)	0/17 (0%)
Co-detection with CPV, CaAstV	6/33 (18.2%)	0/17 (0%)
Co-detection with CDV, CCoV	2/33 (6.1%)	0/17 (0%)
Co-detection with CDV, CaAstV	1/33 (3%)	0/17 (0%)
Co-detection with CPV, CCoV, CaAstV	1/33 (3%)	0/17 (0%)
Co-detection with CPV, CDV, CCoV, CaAstV	1/33 (3%)	0/17 (0%)

[a] canine parvovirus (CPV), canine distemper virus (CDV), canine coronavirus (CCoV), canine astrovirus (CaAstV)

Supplementary Table 3 Information of Thai and Vietnamese canine kobuvirus (CaKoV) strains obtained from this study.

Dog no.	Time of collection	City, Country[a]	Sex[b]	Age (month)	Breed	Clinical signs	Housing	Vaccination history	Deworming	Interact with other pets	GenBank Accession number
G50	Sep 2021	BKK, TH	F	3	Chihuahua	Diarrhea	Indoor	Yes	Yes	No	PP320358
S76	Sep 2021	BKK, TH	M	3	Yorkshire	Healthy	Indoor	Yes	Yes	Dogs	PP320359
S81	Sep 2021	BKK, TH	M	3	Yorkshire	Healthy	Indoor	Yes	Yes	Dogs	PP320360
F8	Feb 2022	NST, TH	M	30	Pomeranian	Healthy	Indoor	Yes	Yes	No	PP320361
V5	Apr 2022	HCM, VN	M	1	Mixed	Diarrhea	Indoor	No	Yes	Dogs	PP320362
V25	Apr 2022	HCM, VN	F	3	Vietnam Local	Diarrhea	Indoor, outdoor	No	Yes	Dogs	PP320363
V91	Apr 2022	HCM, VN	M	2	Vietnam Local	Diarrhea	Indoor, outdoor	No	No	Dogs, Cats	PP320364
V156	Apr 2022	HCM, VN	M	1	Poodle	Diarrhea	Indoor	No	No	Dogs	PP320365
V162	Apr 2022	HCM, VN	F	3	Mixed	Diarrhea	Indoor	No	Yes	Dogs	PP320366
V178	Apr 2022	HCM, VN	M	18	Vietnam Local	Diarrhea	Indoor, outdoor	No	No	No	PP320367
V180	Apr 2022	HCM, VN	F	1	Papillon	Diarrhea	Indoor	Yes	Yes	Dogs	PP320368
V194	Apr 2022	HCM, VN	M	2	Mixed	Healthy	Indoor, outdoor	Yes	Yes	Dogs	PP320369
V196	Apr 2022	HCM, VN	M	2	Mixed	Diarrhea	Indoor, outdoor	No	No	Dogs, Cats	PP320370

[a] BKK, TH (Bangkok, Thailand); NST, TH (Nakhon Si Thamarat, Thailand); HCM, VN (Ho Chi Minh city, Vietnam)

[b] M (Male); F (Female)

Supplementary Table 4 Similarity percentage of nucleotide (nt) and amino acid (aa) of Vietnamese and Thai canine kobuvirus (CaKoV) and other deposited sequences.

CaKoV genome		Within Vietnamese CaKoV sequences	Within Thai CaKoV sequences	Between Vietnamese and Thai CaKoV sequences	Between Vietnamese CaKoV sequences and others	Between Thai CaKoV sequences and others
The CDS	nt	95-97.3%	96.3-99.9%	94.6-96.5%	83.4-89.6%	83.5-95.2%
	aa	92-96.4%	93.6-99.9%	91.9-95%	79.6-94%	79.7-93.6%
P1 region	nt	92.2-96.7%	94.1-99.9%	92.3-95.7%	88.1-96.8%	88.3-95.9%
	aa	85-93.3%	88.2-91.9%	84.8-92.5%	78-94.1%	79.4-92%
VP1 gene	nt	86.2-97.6%	91.7-94.7%	86.8-95.4%	81.6-96.5%	82.1-96%
	aa	89.3-99.6%	95-100%	87.9-97.8%	80-97.8%	81.1-98.5%
P2 region	nt	95.6-98.5%	96.5-100%	95.9-97.8%	93-97.7%	93.5-98.3%
	aa	99.6-100%	100%	99.8-100%	98.5-100%	98.6 -100%
P3 region	nt	96.2-98%	96.1-100%	95.7-97%	94-98.2%	93.7-97.2%
	aa	98.5-99.8%	99.2-100%	98.8-99.7%	97.8-100%	98-99.7%
3D gene	nt	96.7-98.3%	96.7-100%	96.2-97.6%	94-98.7%	93.8-98.3%
	aa	99.2-100%	99.6-100%	99.2-100%	98.1-100%	98.1-100%

Supplementary Table 5 The putative cleavage sites of canine kobuvirus (CaKoV).

Sequences	Accession number	Gene (Amino acid position)									
		**L/VP0 (171/172)**	**VP0/VP3 (553/554)**	**VP3/VP1 (776/777)**	**VP1/2A (1054/1055)**	**2A/2B (1165/1166)**	**2B/2C (1330/1331)**	**2C/3A (1665/1666)**	**3A/3B (1759/1760)**	**3B/3C (1786/1787)**	**3C/3D (2176/2177)**
V25/Vietnam/2022	PP320363 (This study)	Q/G	Q/H	Q/A	Y/V	Q/G	Q/G	Q/G	Q/A	Q/G	Q/G
V178/Vietnam/2022	PP320367 (This study)	Q/G	Q/H	Q/A	Y/V	Q/G	Q/G	Q/G	Q/A	Q/G	Q/G
V180/Vietnam/2022	PP320368 (This study)	Q/G	Q/H	Q/A	Y/V	Q/G	Q/G	Q/G	Q/A	Q/G	Q/G
V5/Vietnam/2022	PP320362 (This study)	Q/G	Q/H	Q/A	Y/V	Q/G	Q/G	Q/G	Q/A	Q/G	Q/G
V194/Vietnam/2022	PP320369 (This study)	Q/G	Q/H	Q/A	Y/V	Q/G	Q/G	Q/G	Q/A	Q/G	Q/G
V91/Vietnam/2022	PP320364 (This study)	Q/G	Q/H	Q/A	Y/V	Q/G	Q/G	Q/G	Q/A	Q/G	Q/G
V156/Vietnam/2022	PP320365 (This study)	Q/G	Q/H	Q/A	Y/V	Q/G	Q/G	Q/G	Q/A	Q/G	Q/G
V162/Vietnam/2022	PP320366 (This study)	Q/G	Q/H	Q/A	Y/V	Q/G	Q/G	Q/G	Q/A	Q/G	Q/G
V196/Vietnam/2022	PP320370 (This study)	Q/G	Q/H	Q/A	Y/V	Q/G	Q/G	Q/G	Q/A	Q/G	Q/G
F8/Thailand/2022	PP320361 (This study)	Q/G	Q/H	Q/A	Y/V	Q/G	Q/G	Q/G	Q/A	Q/G	Q/G
S76/Thailand/2021	PP320359 (This study)	Q/G	Q/H	Q/A	Y/V	Q/G	Q/G	Q/G	Q/A	Q/G	Q/G
S81/Thailand/2021	PP320360 (This study)	Q/G	Q/H	Q/A	Y/V	Q/G	Q/G	Q/G	Q/A	Q/G	Q/G
G50/Thailand/2021	PP320358 (This study)	Q/G	Q/H	Q/A	Y/V	Q/G	Q/G	Q/G	Q/A	Q/G	Q/G
DD2/Tanzania/2003	KM068048.1	Q/G	Q/H	Q/A	Y/V	Q/G	Q/G	Q/G	Q/A	Q/G	Q/G
UK003/UK/2008	KC161964.1	Q/G	Q/H	Q/A	Y/V	Q/G	Q/G	Q/G	Q/A	Q/G	Q/G
82/Tanzania/2010	KM068049.1	Q/G	Q/H	**Q/T**	Y/V	Q/G	Q/G	Q/G	Q/A	Q/G	Q/G
B103/Tanzania/2010	KM068051.1	Q/G	Q/H	**Q/T**	Y/V	Q/G	Q/G	Q/G	Q/A	Q/G	Q/G
US-PC0082/USA/2010	JN088541.1	Q/G	Q/H	Q/A	Y/V	Q/G	Q/G	Q/G	Q/A	Q/G	Q/G
75/Tanzania/2011	KM068050.1	Q/G	Q/H	Q/A	Y/V	Q/G	Q/G	Q/G	Q/A	Q/G	Q/G
12D049/Korea/2012	KF924623.1	Q/G	Q/H	Q/A	Y/V	Q/G	Q/G	Q/G	Q/A	Q/G	Q/G
CE9/Australia/2012	MH052678.1	Q/G	Q/H	Q/A	Y/V	Q/G	Q/G	Q/G	Q/A	Q/G	Q/G
SMCD-59/China/2015	MF062158.1	Q/G	Q/H	Q/A	Y/V	Q/G	Q/G	Q/G	Q/A	Q/G	Q/G
CaKoV-26/Brazil/2016	MH747478.1	Q/G	Q/H	Q/A	Y/V	Q/G	Q/G	Q/G	Q/A	Q/G	Q/G
S272/16/Fox/Germany/2016	MN337880.1	Q/G	Q/H	Q/A	Y/V	Q/G	Q/G	Q/G	Q/A	Q/G	Q/G
CU 53/Thailand/2016	MK201776.1	Q/G	Q/H	Q/A	Y/V	Q/G	Q/G	Q/G	Q/A	Q/G	Q/G
CU 101/Thailand/2016	MK201777.1	Q/G	Q/H	Q/A	Y/V	Q/G	Q/G	Q/G	Q/A	Q/G	Q/G
CU 249/Thailand/2017	MK201778.1	Q/G	Q/H	Q/A	Y/V	Q/G	Q/G	Q/G	Q/A	Q/G	Q/G
CU 716/Thailand/2018	MK201779.1	Q/G	Q/H	Q/A	Y/V	Q/G	Q/G	Q/G	Q/A	Q/G	Q/G
JX-MO1/China/2021	OM451182.1	Q/G	Q/H	Q/A	Y/V	Q/G	Q/G	Q/G	Q/A	Q/G	Q/G

Supplementary Table 6 Genetic analysis VP1 gene of Vietnamese and Thai canine kobuvirus (CaKoV) compared with reference CaKoVs.

Name of sequence	Accession number	Country	Year	Position of amino acids																								
				60	65	67	69	150	151	153	201	204	205	210	213	228	229	230	231	232	233	234	235	236	237	238	239	240
V25/Vietnam/2022	PP320363 (This study)	Vietnam	2022	Q	V	D	T	P	M	D	S	Q	Q	Q	T	P	R	A	P	P	P	L	P	P	L	P	T	P
V178/Vietnam/2022	PP320367 (This study)	Vietnam	2022	Q	V	D	T	P	M	D	S	Q	Q	Q	T	P	R	A	P	P	P	L	P	P	L	P	T	P
V180/Vietnam/2022	PP320368 (This study)	Vietnam	2022	Q	V	D	T	P	M	D	S	Q	Q	Q	T	P	R	A	P	P	P	L	P	P	L	P	T	P
V5/Vietnam/2022	PP320362 (This study)	Vietnam	2022	Q	V	D	T	P	M	D	S	Q	Q	Q	T	P	R	A	P	P	P	L	P	P	L	P	T	P
V194/Vietnam/2022	PP320369 (This study)	Vietnam	2022	Q	V	D	T	P	M	D	S	Q	Q	Q	T	P	R	A	P	P	P	L	P	P	L	P	T	P
V91/Vietnam/2022	PP320364 (This study)	Vietnam	2022	Q	V	D	T	P	M	D	S	Q	Q	Q	T	P	R	A	P	P	P	L	P	P	L	P	T	P
V156/Vietnam/2022	PP320365 (This study)	Vietnam	2022	Q	V	D	T	P	M	D	S	Q	Q	Q	T	P	R	A	P	P	P	L	P	P	L	P	T	P
V162/Vietnam/2022	PP320366 (This study)	Vietnam	2022	N	L	N	A	S	E	N	T	V	E	S	S	P	R	A	P	P	P	L	P	P	L	P	T	P
V196/Vietnam/2022	PP320370 (This study)	Vietnam	2022	N	L	N	A	S	E	N	T	V	E	S	S	P	R	A	P	P	P	L	P	P	L	P	T	P
F8/Thailand/2022	PP320361 (This study)	Thailand	2022	Q	V	D	T	P	M	D	S	Q	Q	Q	T	P	R	A	P	P	P	L	P	P	L	P	T	P
S76/Thailand/2021	PP320359 (This study)	Thailand	2021	Q	V	D	T	P	M	D	S	Q	Q	Q	T	P	R	A	P	P	P	L	P	P	L	P	T	P
S81/Thailand/2021	PP320360 (This study)	Thailand	2021	Q	V	D	T	P	M	D	S	Q	Q	Q	T	P	R	A	P	P	P	L	P	P	L	P	T	P
G50/Thailand/2021	PP320358 (This study)	Thailand	2021	Q	V	D	T	P	M	D	S	Q	Q	Q	T	P	R	A	P	P	P	L	P	P	L	P	T	P
DD2/Tanzania/2003	KM068048.1	Tanzania	2003	N	L	N	A	S	E	N	T	V	E	S	S	P	R	A	P	P	P	L	P	P	L	P	T	P
UK003/UK/2008	KC161964.1	UK	2008	N	L	N	T	S	E	N	T	V	E	S	S	P	R	A	P	P	P	L	P	P	L	P	T	P
AN211D/USA/2009	JN387133.1	USA	2009	N	L	N	A	S	E	N	T	V	E	S	S	P	R	A	P	P	-	L	P	P	L	P	T	P
82/Tanzania/2010	KM068049.1	Tanzania	2010	N	L	N	A	S	E	N	T	A	E	S	S	C	P	V	P	P	P	L	P	P	L	P	T	P
B103/Tanzania/2010	KM068051.1	Tanzania	2010	N	L	N	A	S	E	N	T	A	E	S	S	P	R	A	P	P	P	L	P	P	L	P	T	P
US-PC0082/USA/2010	JN088541.1	USA	2010	N	L	N	T	S	E	N	T	V	E	S	S	C	P	V	P	P	P	L	P	P	L	P	T	P
75/Tanzania/2011	KM068050.1	Tanzania	2011	N	L	N	A	S	E	N	T	A	E	S	S	C	P	V	P	P	P	L	P	P	L	P	T	P
12D049/Korea/2012	KF924623.1	Korea	2012	N	L	N	T	S	E	N	T	V	E	S	S	P	R	A	P	P	P	L	P	P	L	P	T	P
CE9/Australia/2012	MH052678.1	Australia	2012	N	L	N	A	S	E	N	T	V	E	S	S	P	R	A	P	P	P	L	P	P	L	P	T	P
SMCD-59/China/2015	MF062158.1	China	2015	Q	V	D	A	P	M	D	S	Q	Q	Q	T	P	R	A	P	P	P	L	P	P	L	P	T	P
CaKoV-26/Brazil/2016	MH747478.1	Brazil	2016	N	L	N	A	S	E	N	T	V	E	S	S	H	G	A	P	P	P	L	P	P	L	P	T	P
S272/16/Fox/Germany/2016	MN337880.1	Germany	2016	N	L	N	T	S	E	N	T	V	E	S	S	P	R	A	P	P	P	L	P	P	L	P	T	P
CU 53/Thailand/2016	MK201776.1	Thailand	2016	Q	V	D	T	P	M	D	S	Q	Q	Q	T	P	R	A	P	P	P	L	P	P	L	P	T	P
CU 101/Thailand/2016	MK201777.1	Thailand	2016	Q	V	D	T	P	M	D	S	Q	Q	Q	T	P	R	A	P	P	P	L	P	P	L	P	T	P
CU 249/Thailand/2017	MK201778.1	Thailand	2017	Q	V	D	A	P	M	D	S	Q	Q	Q	T	P	R	A	P	P	P	L	P	P	L	P	T	P
CU 716/Thailand/2018	MK201779.1	Thailand	2018	Q	V	D	T	P	M	D	S	Q	Q	Q	T	P	R	A	P	P	P	L	P	P	L	P	T	P
CaKoV AH-1/China/2019	MN449341.1	China	2019	Q	V	D	T	P	M	D	S	Q	Q	Q	T	P	R	A	P	P	P	L	P	P	L	P	T	P
JX-MO1/China/2021	OM451182.1	China	2021	Q	V	N	T	P	M	D	S	Q	Q	Q	T	P	R	A	P	P	P	L	P	P	L	P	T	P

Supplementary Table 7 Setting parameters of methods to predict continuous B-cell epitope of VP1 capsid gene of canine kobuvirus (CaKoV) from IEDB database.

Method	Average score	Maximum score	Minimum score	Threshold
Kolaskar and Tongaonkar antigenicity	1.026	1.167	0.883	1.026
Bepipred linear epitope prediction	0.474	0.642	0.279	0.5
Parker hydrophilicity	1.353	6.586	-5.129	1.353
Emini surface accessibility	1.000	4.488	0.107	1.000
Chou and Fasman beta turn	1.047	1.391	0.648	1.047
Karplus and Schulz flexibility prediction	1.005	1.105	0.897	1.005

Supplementary Table 8 Information analysis of discontinuous B-cell epitope predicted from the VP1 capsid of Vietnamese and Thai canine kobuvirus (CaKoV) in this study.

No.	Residues	Number of residues	Score
1	A1, N2, S3, E4, D5, L6, N7, A8, P9, Q10, D11, T12, A13, N14, I15, E16, N17, G18, A19, S20, D21, N22, T23, P24, Q25, P26, R27, T28, T29, F30, E31, Y32	32	0.848
2	L234, P235, L237, E241, T242, N243, P244, E245, K246, S247, V248, A249, V250, V251, K252, Q253, G254, A255, H256, T257, S258, L259, G260, D261, V262, D263, P264, D265, D266, R267, V268, Y269, I270, V271, R272, D274	36	0.685
3	I109, S110, N111, P112, N113, G114, L115, P116, T117, S118, E146, T147, P148, I149, P150, M151, S152, D153, T154, T155, L156, S201, Y202, P203, Q204, T213	26	0.677
4	S165, P166, L167, S168, A169, S173, Y174, G176, W177, E178, D179, W180, S181, G182, T183, N184, F185, G186, V187, L188, Q189, A190	22	0.64
5	E34, N35, S36, L37, P38, P39, D40, S41	8	0.632
6	P64, V65, P66, D67, G68, T69, P125, P126, G127, A128, T129, I130, P131, Q132, S133, P134, D135, Q137, T138, S140, N141, F142, M144, Q205, D206, V207, Q208, P209, Q210, P211, M212	31	0.608

Supplementary Table 9 Nucleotide and deduced amino acid similarities of CaKoV

Supplementary Table 9a Nucleotide sequence similarities of the CaKoV complete coding sequences (CDSs)

	Sequences	1	2	3	4	5	6	7	8	9	10	11	12	13	14	15	16	17	18	19	20	21	22	23	24	25	26	27	28	29	30	31	32	33
**1**	**PP320363**	ID	0.969	0.968	0.966	0.968	0.965	0.96	0.954	0.955	0.965	0.96	0.96	0.961	0.869	0.896	0.945	0.95	0.953	0.952	0.876	0.848	0.85	0.888	0.859	0.882	0.891	0.843	0.839	0.843	0.854	0.867	0.868	0.846
**2**	**PP320367**	0.969	ID	0.973	0.973	0.965	0.964	0.959	0.954	0.953	0.965	0.96	0.96	0.961	0.867	0.891	0.945	0.948	0.95	0.952	0.877	0.847	0.848	0.886	0.86	0.881	0.889	0.841	0.836	0.84	0.855	0.867	0.866	0.841
**3**	**PP320368**	0.968	0.973	ID	0.972	0.96	0.96	0.954	0.951	0.951	0.962	0.955	0.955	0.957	0.865	0.89	0.944	0.946	0.949	0.951	0.874	0.844	0.845	0.886	0.857	0.879	0.887	0.838	0.834	0.837	0.851	0.863	0.864	0.841
**4**	**PP320362**	0.966	0.973	0.972	ID	0.961	0.962	0.954	0.949	0.95	0.962	0.957	0.957	0.959	0.864	0.887	0.941	0.945	0.947	0.948	0.873	0.843	0.845	0.884	0.856	0.878	0.885	0.839	0.836	0.839	0.851	0.863	0.861	0.841
**5**	**PP320369**	0.968	0.965	0.96	0.961	ID	0.963	0.959	0.951	0.953	0.964	0.958	0.958	0.958	0.865	0.89	0.944	0.947	0.95	0.95	0.873	0.845	0.844	0.885	0.857	0.882	0.89	0.841	0.837	0.841	0.854	0.867	0.862	0.843
**6**	**PP320364**	0.965	0.964	0.96	0.962	0.963	ID	0.959	0.952	0.952	0.962	0.955	0.955	0.958	0.863	0.89	0.943	0.945	0.947	0.947	0.875	0.843	0.844	0.884	0.856	0.88	0.887	0.839	0.835	0.839	0.852	0.863	0.863	0.842
**7**	**PP320365**	0.96	0.959	0.954	0.954	0.959	0.959	ID	0.95	0.951	0.956	0.95	0.95	0.955	0.861	0.884	0.936	0.941	0.943	0.943	0.871	0.842	0.845	0.881	0.855	0.878	0.882	0.838	0.834	0.838	0.853	0.863	0.86	0.843
**8**	**PP320366**	0.954	0.954	0.951	0.949	0.951	0.952	0.95	ID	0.963	0.95	0.946	0.946	0.949	0.857	0.879	0.932	0.939	0.94	0.939	0.876	0.849	0.848	0.873	0.859	0.882	0.881	0.84	0.837	0.841	0.854	0.865	0.865	0.845
**9**	**PP320370**	0.955	0.953	0.951	0.95	0.953	0.952	0.951	0.963	ID	0.951	0.947	0.947	0.95	0.857	0.878	0.931	0.937	0.938	0.939	0.878	0.848	0.849	0.875	0.858	0.884	0.88	0.842	0.837	0.842	0.853	0.867	0.865	0.847
**10**	**PP320361**	0.965	0.965	0.962	0.962	0.964	0.962	0.956	0.95	0.951	ID	0.965	0.965	0.963	0.864	0.889	0.946	0.949	0.951	0.952	0.875	0.845	0.846	0.886	0.859	0.881	0.887	0.84	0.837	0.841	0.854	0.865	0.863	0.841
**11**	**PP320359**	0.96	0.96	0.955	0.957	0.958	0.955	0.95	0.946	0.947	0.965	ID	0.999	0.956	0.861	0.884	0.939	0.945	0.943	0.942	0.871	0.842	0.844	0.88	0.856	0.879	0.881	0.839	0.835	0.839	0.85	0.862	0.861	0.84
**12**	**PP320360**	0.96	0.96	0.955	0.957	0.958	0.955	0.95	0.946	0.947	0.965	0.999	ID	0.956	0.861	0.884	0.939	0.945	0.943	0.942	0.871	0.842	0.844	0.88	0.856	0.879	0.881	0.839	0.835	0.839	0.85	0.862	0.861	0.84
**13**	**PP320358**	0.961	0.961	0.957	0.959	0.958	0.958	0.955	0.949	0.95	0.963	0.956	0.956	ID	0.862	0.887	0.943	0.946	0.949	0.95	0.873	0.845	0.845	0.881	0.854	0.879	0.883	0.838	0.835	0.839	0.852	0.865	0.86	0.84
**14**	**OM451182.1**	0.869	0.867	0.865	0.864	0.865	0.863	0.861	0.857	0.857	0.864	0.861	0.861	0.862	ID	0.934	0.878	0.881	0.884	0.884	0.899	0.921	0.92	0.93	0.932	0.903	0.935	0.913	0.909	0.914	0.928	0.906	0.914	0.914
**15**	**MN449341.1**	0.896	0.891	0.89	0.887	0.89	0.89	0.884	0.879	0.878	0.889	0.884	0.884	0.887	0.934	ID	0.903	0.907	0.91	0.91	0.917	0.916	0.916	0.965	0.93	0.921	0.97	0.909	0.905	0.908	0.919	0.927	0.935	0.912
**16**	**MK201779.1**	0.945	0.945	0.944	0.941	0.944	0.943	0.936	0.932	0.931	0.946	0.939	0.939	0.943	0.878	0.903	ID	0.97	0.965	0.964	0.872	0.857	0.86	0.898	0.868	0.893	0.901	0.851	0.846	0.85	0.864	0.879	0.877	0.853
**17**	**MK201778.1**	0.95	0.948	0.946	0.945	0.947	0.945	0.941	0.939	0.937	0.949	0.945	0.945	0.946	0.881	0.907	0.97	ID	0.968	0.967	0.878	0.863	0.862	0.902	0.874	0.898	0.907	0.854	0.849	0.854	0.87	0.883	0.881	0.857
**18**	**MK201776.1**	0.953	0.95	0.949	0.947	0.95	0.947	0.943	0.94	0.938	0.951	0.943	0.943	0.949	0.884	0.91	0.965	0.968	ID	0.992	0.88	0.861	0.862	0.908	0.874	0.896	0.908	0.854	0.851	0.855	0.869	0.882	0.879	0.857
**19**	**MK201777.1**	0.952	0.952	0.951	0.948	0.95	0.947	0.943	0.939	0.939	0.952	0.942	0.942	0.95	0.884	0.91	0.964	0.967	0.992	ID	0.88	0.861	0.862	0.909	0.873	0.896	0.908	0.854	0.851	0.854	0.869	0.881	0.879	0.857
**20**	**MT610361.1**	0.876	0.877	0.874	0.873	0.873	0.875	0.871	0.876	0.878	0.875	0.871	0.871	0.873	0.899	0.917	0.872	0.878	0.88	0.88	ID	0.896	0.892	0.913	0.906	0.906	0.924	0.885	0.882	0.886	0.9	0.9	0.911	0.89
**21**	**MH747478.1**	0.848	0.847	0.844	0.843	0.845	0.843	0.842	0.849	0.848	0.845	0.842	0.842	0.845	0.921	0.916	0.857	0.863	0.861	0.861	0.896	ID	0.934	0.912	0.933	0.902	0.922	0.93	0.925	0.929	0.931	0.914	0.927	0.93
**22**	**MN337880.1**	0.85	0.848	0.845	0.845	0.844	0.844	0.845	0.848	0.849	0.846	0.844	0.844	0.845	0.92	0.916	0.86	0.862	0.862	0.862	0.892	0.934	ID	0.911	0.928	0.901	0.918	0.926	0.924	0.926	0.929	0.912	0.936	0.936
**23**	**MF062158.1**	0.888	0.886	0.886	0.884	0.885	0.884	0.881	0.873	0.875	0.886	0.88	0.88	0.881	0.93	0.965	0.898	0.902	0.908	0.909	0.913	0.912	0.911	ID	0.928	0.918	0.964	0.904	0.901	0.904	0.914	0.923	0.928	0.906
**24**	**KF924623.1**	0.859	0.86	0.857	0.856	0.857	0.856	0.855	0.859	0.858	0.859	0.856	0.856	0.854	0.932	0.93	0.868	0.874	0.874	0.873	0.906	0.933	0.928	0.928	ID	0.913	0.935	0.92	0.917	0.922	0.933	0.917	0.926	0.926
**25**	**MH052678.1**	0.882	0.881	0.879	0.878	0.882	0.88	0.878	0.882	0.884	0.881	0.879	0.879	0.879	0.903	0.921	0.893	0.898	0.896	0.896	0.906	0.902	0.901	0.918	0.913	ID	0.926	0.896	0.891	0.897	0.919	0.929	0.92	0.898
**26**	**JQ911763.1**	0.891	0.889	0.887	0.885	0.89	0.887	0.882	0.881	0.88	0.887	0.881	0.881	0.883	0.935	0.97	0.901	0.907	0.908	0.908	0.924	0.922	0.918	0.964	0.935	0.926	ID	0.915	0.912	0.914	0.924	0.932	0.938	0.917
**27**	**KM068050.1**	0.843	0.841	0.838	0.839	0.841	0.839	0.838	0.84	0.842	0.84	0.839	0.839	0.838	0.913	0.909	0.851	0.854	0.854	0.854	0.885	0.93	0.926	0.904	0.92	0.896	0.915	ID	0.977	0.981	0.921	0.907	0.923	0.937
**28**	**KM068049.1**	0.839	0.836	0.834	0.836	0.837	0.835	0.834	0.837	0.837	0.837	0.835	0.835	0.835	0.909	0.905	0.846	0.849	0.851	0.851	0.882	0.925	0.924	0.901	0.917	0.891	0.912	0.977	ID	0.974	0.918	0.904	0.919	0.946
**29**	**KM068051.1**	0.843	0.84	0.837	0.839	0.841	0.839	0.838	0.841	0.842	0.841	0.839	0.839	0.839	0.914	0.908	0.85	0.854	0.855	0.854	0.886	0.929	0.926	0.904	0.922	0.897	0.914	0.981	0.974	ID	0.922	0.907	0.924	0.939
**30**	**JN088541.1**	0.854	0.855	0.851	0.851	0.854	0.852	0.853	0.854	0.853	0.854	0.85	0.85	0.852	0.928	0.919	0.864	0.87	0.869	0.869	0.9	0.931	0.929	0.914	0.933	0.919	0.924	0.921	0.918	0.922	ID	0.941	0.926	0.926
**31**	**JN387133.1**	0.867	0.867	0.863	0.863	0.867	0.863	0.863	0.865	0.867	0.865	0.862	0.862	0.865	0.906	0.927	0.879	0.883	0.882	0.881	0.9	0.914	0.912	0.923	0.917	0.929	0.932	0.907	0.904	0.907	0.941	ID	0.932	0.911
**32**	**KC161964.1**	0.868	0.866	0.864	0.861	0.862	0.863	0.86	0.865	0.865	0.863	0.861	0.861	0.86	0.914	0.935	0.877	0.881	0.879	0.879	0.911	0.927	0.936	0.928	0.926	0.92	0.938	0.923	0.919	0.924	0.926	0.932	ID	0.928
**33**	**KM068048.1**	0.846	0.841	0.841	0.841	0.843	0.842	0.843	0.845	0.847	0.841	0.84	0.84	0.84	0.914	0.912	0.853	0.857	0.857	0.857	0.89	0.93	0.936	0.906	0.926	0.898	0.917	0.937	0.946	0.939	0.926	0.911	0.928	ID

Supplementary Table 9b Deduced amino acid sequence similarities of the CaKoV complete coding sequences (CDSs)

	Sequences	1	2	3	4	5	6	7	8	9	10	11	12	13	14	15	16	17	18	19	20	21	22	23	24	25	26	27	28	29	30	31	32	33
**1**	**PP320363**	ID	0.95	0.946	0.949	0.956	0.955	0.941	0.926	0.929	0.95	0.942	0.941	0.943	0.85	0.891	0.929	0.939	0.94	0.939	0.845	0.817	0.822	0.874	0.826	0.839	0.88	0.811	0.807	0.812	0.823	0.835	0.843	0.819
**2**	**PP320367**	0.95	ID	0.957	0.963	0.944	0.943	0.934	0.924	0.923	0.947	0.938	0.937	0.941	0.842	0.876	0.927	0.928	0.933	0.933	0.842	0.813	0.815	0.868	0.822	0.839	0.873	0.807	0.802	0.81	0.824	0.834	0.84	0.809
**3**	**PP320368**	0.946	0.957	ID	0.964	0.937	0.936	0.926	0.921	0.921	0.943	0.935	0.934	0.939	0.843	0.879	0.924	0.929	0.936	0.937	0.839	0.807	0.809	0.874	0.82	0.832	0.871	0.8	0.796	0.802	0.818	0.826	0.835	0.809
**4**	**PP320362**	0.949	0.963	0.964	ID	0.944	0.944	0.932	0.924	0.923	0.947	0.938	0.937	0.942	0.845	0.876	0.924	0.928	0.933	0.934	0.842	0.812	0.814	0.869	0.822	0.842	0.873	0.807	0.805	0.811	0.823	0.833	0.837	0.811
**5**	**PP320369**	0.956	0.944	0.937	0.944	ID	0.951	0.931	0.923	0.925	0.943	0.936	0.936	0.937	0.841	0.878	0.927	0.933	0.931	0.932	0.838	0.811	0.81	0.87	0.82	0.838	0.875	0.804	0.803	0.806	0.818	0.83	0.83	0.812
**6**	**PP320364**	0.955	0.943	0.936	0.944	0.951	ID	0.939	0.922	0.925	0.942	0.933	0.933	0.937	0.841	0.881	0.924	0.926	0.927	0.926	0.84	0.807	0.81	0.869	0.82	0.836	0.872	0.807	0.801	0.81	0.819	0.828	0.836	0.814
**7**	**PP320365**	0.941	0.934	0.926	0.932	0.931	0.939	ID	0.92	0.922	0.929	0.921	0.921	0.935	0.837	0.865	0.913	0.918	0.919	0.918	0.837	0.808	0.814	0.86	0.822	0.836	0.862	0.807	0.802	0.81	0.823	0.831	0.836	0.817
**8**	**PP320366**	0.926	0.924	0.921	0.924	0.923	0.922	0.92	ID	0.955	0.919	0.921	0.92	0.922	0.828	0.856	0.906	0.913	0.912	0.912	0.848	0.82	0.824	0.847	0.828	0.844	0.863	0.81	0.805	0.812	0.824	0.833	0.843	0.824
**9**	**PP320370**	0.929	0.923	0.921	0.923	0.925	0.925	0.922	0.955	ID	0.921	0.92	0.919	0.923	0.827	0.859	0.905	0.914	0.912	0.913	0.851	0.82	0.827	0.848	0.828	0.848	0.862	0.814	0.808	0.815	0.825	0.835	0.841	0.828
**10**	**PP320361**	0.95	0.947	0.943	0.947	0.943	0.942	0.929	0.919	0.921	ID	0.943	0.942	0.945	0.843	0.876	0.93	0.936	0.936	0.935	0.843	0.811	0.813	0.871	0.824	0.838	0.87	0.806	0.803	0.807	0.823	0.832	0.836	0.81
**11**	**PP320359**	0.942	0.938	0.935	0.938	0.936	0.933	0.921	0.921	0.92	0.943	ID	0.999	0.936	0.841	0.869	0.92	0.935	0.925	0.922	0.841	0.808	0.81	0.862	0.825	0.839	0.864	0.802	0.798	0.805	0.816	0.827	0.831	0.808
**12**	**PP320360**	0.941	0.937	0.934	0.937	0.936	0.933	0.921	0.92	0.919	0.942	0.999	ID	0.936	0.84	0.869	0.92	0.935	0.924	0.922	0.84	0.808	0.809	0.862	0.825	0.838	0.863	0.802	0.797	0.804	0.816	0.826	0.831	0.807
**13**	**PP320358**	0.943	0.941	0.939	0.942	0.937	0.937	0.935	0.922	0.923	0.945	0.936	0.936	ID	0.841	0.875	0.928	0.933	0.932	0.935	0.843	0.811	0.813	0.868	0.821	0.84	0.866	0.806	0.801	0.81	0.823	0.834	0.836	0.813
**14**	**OM451182.1**	0.85	0.842	0.843	0.845	0.841	0.841	0.837	0.828	0.827	0.843	0.841	0.84	0.841	ID	0.908	0.86	0.861	0.864	0.862	0.862	0.875	0.875	0.902	0.889	0.855	0.907	0.868	0.864	0.871	0.885	0.861	0.873	0.873
**15**	**MN449341.1**	0.891	0.876	0.879	0.876	0.878	0.881	0.865	0.856	0.859	0.876	0.869	0.869	0.875	0.908	ID	0.89	0.895	0.901	0.901	0.885	0.879	0.879	0.948	0.888	0.875	0.955	0.869	0.865	0.871	0.883	0.886	0.903	0.879
**16**	**MK201779.1**	0.929	0.927	0.924	0.924	0.927	0.924	0.913	0.906	0.905	0.93	0.92	0.92	0.928	0.86	0.89	ID	0.955	0.952	0.948	0.839	0.825	0.83	0.883	0.832	0.852	0.889	0.815	0.812	0.816	0.833	0.843	0.847	0.825
**17**	**MK201778.1**	0.939	0.928	0.929	0.928	0.933	0.926	0.918	0.913	0.914	0.936	0.935	0.935	0.933	0.861	0.895	0.955	ID	0.954	0.952	0.849	0.832	0.83	0.89	0.843	0.862	0.896	0.821	0.816	0.823	0.84	0.852	0.855	0.828
**18**	**MK201776.1**	0.94	0.933	0.936	0.933	0.931	0.927	0.919	0.912	0.912	0.936	0.925	0.924	0.932	0.864	0.901	0.952	0.954	ID	0.994	0.849	0.825	0.833	0.902	0.839	0.854	0.896	0.821	0.818	0.824	0.839	0.847	0.851	0.829
**19**	**MK201777.1**	0.939	0.933	0.937	0.934	0.932	0.926	0.918	0.912	0.913	0.935	0.922	0.922	0.935	0.862	0.901	0.948	0.952	0.994	ID	0.849	0.824	0.832	0.902	0.837	0.854	0.896	0.823	0.819	0.826	0.839	0.847	0.851	0.83
**20**	**MT610361.1**	0.845	0.842	0.839	0.842	0.838	0.84	0.837	0.848	0.851	0.843	0.841	0.84	0.843	0.862	0.885	0.839	0.849	0.849	0.849	ID	0.863	0.859	0.878	0.876	0.868	0.894	0.849	0.845	0.853	0.87	0.866	0.886	0.86
**21**	**MH747478.1**	0.817	0.813	0.807	0.812	0.811	0.807	0.808	0.82	0.82	0.811	0.808	0.808	0.811	0.875	0.879	0.825	0.832	0.825	0.824	0.863	ID	0.898	0.865	0.895	0.867	0.884	0.897	0.892	0.896	0.892	0.877	0.892	0.897
**22**	**MN337880.1**	0.822	0.815	0.809	0.814	0.81	0.81	0.814	0.824	0.827	0.813	0.81	0.809	0.813	0.875	0.879	0.83	0.83	0.833	0.832	0.859	0.898	ID	0.869	0.89	0.865	0.881	0.888	0.887	0.89	0.89	0.874	0.915	0.909
**23**	**MF062158.1**	0.874	0.868	0.874	0.869	0.87	0.869	0.86	0.847	0.848	0.871	0.862	0.862	0.868	0.902	0.948	0.883	0.89	0.902	0.902	0.878	0.865	0.869	ID	0.885	0.873	0.947	0.86	0.857	0.864	0.874	0.878	0.89	0.868
**24**	**KF924623.1**	0.826	0.822	0.82	0.822	0.82	0.82	0.822	0.828	0.828	0.824	0.825	0.825	0.821	0.889	0.888	0.832	0.843	0.839	0.837	0.876	0.895	0.89	0.885	ID	0.876	0.896	0.879	0.874	0.883	0.902	0.886	0.898	0.891
**25**	**MH052678.1**	0.839	0.839	0.832	0.842	0.838	0.836	0.836	0.844	0.848	0.838	0.839	0.838	0.84	0.855	0.875	0.852	0.862	0.854	0.854	0.868	0.867	0.865	0.873	0.876	ID	0.883	0.855	0.849	0.858	0.898	0.904	0.886	0.864
**26**	**JQ911763.1**	0.88	0.873	0.871	0.873	0.875	0.872	0.862	0.863	0.862	0.87	0.864	0.863	0.866	0.907	0.955	0.889	0.896	0.896	0.896	0.894	0.884	0.881	0.947	0.896	0.883	ID	0.877	0.874	0.877	0.888	0.893	0.905	0.884
**27**	**KM068050.1**	0.811	0.807	0.8	0.807	0.804	0.807	0.807	0.81	0.814	0.806	0.802	0.802	0.806	0.868	0.869	0.815	0.821	0.821	0.823	0.849	0.897	0.888	0.86	0.879	0.855	0.877	ID	0.966	0.97	0.883	0.867	0.89	0.908
**28**	**KM068049.1**	0.807	0.802	0.796	0.805	0.803	0.801	0.802	0.805	0.808	0.803	0.798	0.797	0.801	0.864	0.865	0.812	0.816	0.818	0.819	0.845	0.892	0.887	0.857	0.874	0.849	0.874	0.966	ID	0.963	0.876	0.862	0.886	0.92
**29**	**KM068051.1**	0.812	0.81	0.802	0.811	0.806	0.81	0.81	0.812	0.815	0.807	0.805	0.804	0.81	0.871	0.871	0.816	0.823	0.824	0.826	0.853	0.896	0.89	0.864	0.883	0.858	0.877	0.97	0.963	ID	0.884	0.871	0.896	0.912
**30**	**JN088541.1**	0.823	0.824	0.818	0.823	0.818	0.819	0.823	0.824	0.825	0.823	0.816	0.816	0.823	0.885	0.883	0.833	0.84	0.839	0.839	0.87	0.892	0.89	0.874	0.902	0.898	0.888	0.883	0.876	0.884	ID	0.924	0.894	0.889
**31**	**JN387133.1**	0.835	0.834	0.826	0.833	0.83	0.828	0.831	0.833	0.835	0.832	0.827	0.826	0.834	0.861	0.886	0.843	0.852	0.847	0.847	0.866	0.877	0.874	0.878	0.886	0.904	0.893	0.867	0.862	0.871	0.924	ID	0.899	0.874
**32**	**KC161964.1**	0.843	0.84	0.835	0.837	0.83	0.836	0.836	0.843	0.841	0.836	0.831	0.831	0.836	0.873	0.903	0.847	0.855	0.851	0.851	0.886	0.892	0.915	0.89	0.898	0.886	0.905	0.89	0.886	0.896	0.894	0.899	ID	0.905
**33**	**KM068048.1**	0.819	0.809	0.809	0.811	0.812	0.814	0.817	0.824	0.828	0.81	0.808	0.807	0.813	0.873	0.879	0.825	0.828	0.829	0.83	0.86	0.897	0.909	0.868	0.891	0.864	0.884	0.908	0.92	0.912	0.889	0.874	0.905	ID

Supplementary Table 9c Nucleotide sequence similarities of the CaKoV P1 region

	Sequences	1	2	3	4	5	6	7	8	9	10	11	12	13	14	15	16	17	18	19	20	21	22	23	24	25	26	27	28	29	30	31	32	33
**1**	**PP320363**	ID	0.96	0.959	0.956	0.964	0.961	0.94	0.932	0.931	0.957	0.952	0.952	0.951	0.934	0.968	0.954	0.959	0.961	0.961	0.913	0.894	0.904	0.953	0.909	0.901	0.951	0.895	0.892	0.895	0.9	0.902	0.915	0.899
**2**	**PP320367**	0.96	ID	0.966	0.967	0.951	0.953	0.937	0.929	0.929	0.952	0.946	0.946	0.949	0.928	0.955	0.95	0.948	0.954	0.955	0.909	0.89	0.901	0.946	0.909	0.898	0.941	0.89	0.889	0.89	0.904	0.906	0.912	0.891
**3**	**PP320368**	0.959	0.966	ID	0.967	0.947	0.948	0.935	0.929	0.928	0.952	0.946	0.945	0.95	0.931	0.963	0.951	0.95	0.961	0.963	0.906	0.888	0.896	0.954	0.906	0.899	0.943	0.886	0.884	0.886	0.901	0.898	0.91	0.892
**4**	**PP320362**	0.956	0.967	0.967	ID	0.948	0.952	0.933	0.926	0.927	0.952	0.946	0.946	0.951	0.929	0.956	0.948	0.948	0.955	0.956	0.906	0.89	0.899	0.947	0.904	0.901	0.938	0.887	0.887	0.888	0.9	0.9	0.907	0.89
**5**	**PP320369**	0.964	0.951	0.947	0.948	ID	0.956	0.932	0.924	0.929	0.949	0.945	0.945	0.946	0.925	0.956	0.948	0.953	0.95	0.952	0.906	0.886	0.894	0.945	0.905	0.902	0.941	0.887	0.888	0.885	0.898	0.901	0.903	0.893
**6**	**PP320364**	0.961	0.953	0.948	0.952	0.956	ID	0.94	0.929	0.932	0.95	0.943	0.943	0.948	0.924	0.96	0.951	0.947	0.951	0.952	0.907	0.884	0.895	0.947	0.906	0.899	0.942	0.89	0.887	0.891	0.899	0.899	0.909	0.895
**7**	**PP320365**	0.94	0.937	0.935	0.933	0.932	0.94	ID	0.924	0.922	0.932	0.925	0.924	0.938	0.918	0.938	0.933	0.934	0.937	0.938	0.902	0.881	0.899	0.932	0.901	0.894	0.925	0.887	0.884	0.886	0.899	0.897	0.904	0.897
**8**	**PP320366**	0.932	0.929	0.929	0.926	0.924	0.929	0.924	ID	0.97	0.923	0.925	0.924	0.925	0.91	0.931	0.926	0.93	0.932	0.932	0.92	0.906	0.917	0.921	0.921	0.914	0.929	0.901	0.898	0.901	0.907	0.909	0.921	0.912
**9**	**PP320370**	0.931	0.929	0.928	0.927	0.929	0.932	0.922	0.97	ID	0.924	0.923	0.923	0.928	0.906	0.932	0.924	0.931	0.931	0.932	0.922	0.903	0.918	0.922	0.916	0.914	0.929	0.901	0.896	0.9	0.906	0.907	0.923	0.913
**10**	**PP320361**	0.957	0.952	0.952	0.952	0.949	0.95	0.932	0.923	0.924	ID	0.951	0.951	0.951	0.927	0.955	0.951	0.954	0.959	0.958	0.907	0.883	0.896	0.95	0.907	0.901	0.937	0.889	0.888	0.889	0.899	0.9	0.906	0.889
**11**	**PP320359**	0.952	0.946	0.946	0.946	0.945	0.943	0.925	0.925	0.923	0.951	ID	0.999	0.941	0.924	0.949	0.943	0.952	0.948	0.947	0.906	0.884	0.895	0.942	0.907	0.899	0.931	0.888	0.887	0.887	0.894	0.896	0.904	0.89
**12**	**PP320360**	0.952	0.946	0.945	0.946	0.945	0.943	0.924	0.924	0.923	0.951	0.999	ID	0.941	0.924	0.949	0.943	0.952	0.948	0.946	0.906	0.884	0.895	0.942	0.906	0.898	0.931	0.888	0.886	0.887	0.894	0.895	0.903	0.89
**13**	**PP320358**	0.951	0.949	0.95	0.951	0.946	0.948	0.938	0.925	0.928	0.951	0.941	0.941	ID	0.926	0.956	0.953	0.953	0.956	0.959	0.909	0.886	0.901	0.949	0.905	0.901	0.935	0.889	0.887	0.89	0.901	0.903	0.907	0.895
**14**	**OM451182.1**	0.934	0.928	0.931	0.929	0.925	0.924	0.918	0.91	0.906	0.927	0.924	0.924	0.926	ID	0.93	0.929	0.929	0.935	0.933	0.903	0.882	0.893	0.927	0.899	0.886	0.926	0.879	0.88	0.882	0.892	0.887	0.896	0.887
**15**	**MN449341.1**		0.955	0.963	0.956	0.956	0.96	0.938	0.931	0.932	0.955	0.949	0.949	0.956	0.93	ID	0.954	0.955	0.966	0.966	0.914	0.89	0.899	0.959	0.906	0.902	0.954	0.891	0.891	0.892	0.901	0.899	0.913	0.896
**16**	**MK201779.1**	0.954	0.95	0.951	0.948	0.948	0.951	0.933	0.926	0.924	0.951	0.943	0.943	0.953	0.929	0.954	ID	0.957	0.956	0.955	0.904	0.886	0.901	0.949	0.903	0.899	0.943	0.885	0.884	0.884	0.898	0.899	0.905	0.891
**17**	**MK201778.1**	0.959	0.948	0.95	0.948	0.953	0.947	0.934	0.93	0.931	0.954	0.952	0.952	0.953	0.929	0.955	0.957	ID	0.958	0.958	0.91	0.894	0.9	0.952	0.912	0.907	0.948	0.888	0.886	0.886	0.903	0.904	0.91	0.893
**18**	**MK201776.1**	0.961	0.954	0.961	0.955	0.95	0.951	0.937	0.932	0.931	0.959	0.948	0.948	0.956	0.935	0.966	0.956	0.958	ID	0.995	0.915	0.887	0.902	0.969	0.909	0.901	0.951	0.889	0.89	0.891	0.903	0.9	0.909	0.896
**19**	**MK201777.1**	0.961	0.955	0.963	0.956	0.952	0.952	0.938	0.932	0.932	0.958	0.947	0.946	0.959	0.933	0.966	0.955	0.958	0.995	ID	0.914	0.886	0.902	0.969	0.907	0.902	0.951	0.89	0.891	0.891	0.903	0.9	0.91	0.896
**20**	**MT610361.1**	0.913	0.909	0.906	0.906	0.906	0.907	0.902	0.92	0.922	0.907	0.906	0.906	0.909	0.903	0.914	0.904	0.91	0.915	0.914	ID	0.907	0.912	0.906	0.927	0.912	0.92	0.899	0.898	0.902	0.917	0.91	0.923	0.908
**21**	**MH747478.1**	0.894	0.89	0.888	0.89	0.886	0.884	0.881	0.906	0.903	0.883	0.884	0.884	0.886	0.882	0.89	0.886	0.894	0.887	0.886	0.907	ID	0.909	0.882	0.905	0.899	0.896	0.896	0.895	0.898	0.901	0.9	0.915	0.901
**22**	**MN337880.1**	0.904	0.901	0.896	0.899	0.894	0.895	0.899	0.917	0.918	0.896	0.895	0.895	0.901	0.893	0.899	0.901	0.9	0.902	0.902	0.912	0.909	ID	0.893	0.908	0.901	0.903	0.904	0.909	0.907	0.904	0.903	0.939	0.924
**23**	**MF062158.1**	0.953	0.946	0.954	0.947	0.945	0.947	0.932	0.921	0.922	0.95	0.942	0.942	0.949	0.927	0.959	0.949	0.952	0.969	0.969	0.906	0.882	0.893	ID	0.902	0.899	0.947	0.885	0.885	0.887	0.894	0.894	0.902	0.888
**24**	**KF924623.1**	0.909	0.909	0.906	0.904	0.905	0.906	0.901	0.921	0.916	0.907	0.907	0.906	0.905	0.899	0.906	0.903	0.912	0.909	0.907	0.927	0.905	0.908	0.902	ID	0.919	0.911	0.897	0.898	0.901	0.921	0.92	0.926	0.912
**25**	**MH052678.1**	0.901	0.898	0.899	0.901	0.902	0.899	0.894	0.914	0.914	0.901	0.899	0.898	0.901	0.886	0.902	0.899	0.907	0.901	0.902	0.912	0.899	0.901	0.899	0.919	ID	0.905	0.895	0.893	0.896	0.937	0.933	0.916	0.903
**26**	**JQ911763.1**	0.951	0.941	0.943	0.938	0.941	0.942	0.925	0.929	0.929	0.937	0.931	0.931	0.935	0.926	0.954	0.943	0.948	0.951	0.951	0.92	0.896	0.903	0.947	0.911	0.905	ID	0.899	0.899	0.895	0.908	0.904	0.912	0.902
**27**	**KM068050.1**	0.895	0.89	0.886	0.887	0.887	0.89	0.887	0.901	0.901	0.889	0.888	0.888	0.889	0.879	0.891	0.885	0.888	0.889	0.89	0.899	0.896	0.904	0.885	0.897	0.895	0.899	ID	0.972	0.975	0.89	0.895	0.914	0.916
**28**	**KM068049.1**	0.892	0.889	0.884	0.887	0.888	0.887	0.884	0.898	0.896	0.888	0.887	0.886	0.887	0.88	0.891	0.884	0.886	0.89	0.891	0.898	0.895	0.909	0.885	0.898	0.893	0.899	0.972	ID	0.973	0.889	0.896	0.915	0.93
**29**	**KM068051.1**	0.895	0.89	0.886	0.888	0.885	0.891	0.886	0.901	0.9	0.889	0.887	0.887	0.89	0.882	0.892	0.884	0.886	0.891	0.891	0.902	0.898	0.907	0.887	0.901	0.896	0.895	0.975	0.973	ID	0.89	0.896	0.919	0.924
**30**	**JN088541.1**	0.9	0.904	0.901	0.9	0.898	0.899	0.899	0.907	0.906	0.899	0.894	0.894	0.901	0.892	0.901	0.898	0.903	0.903	0.903	0.917	0.901	0.904	0.894	0.921	0.937	0.908	0.89	0.889	0.89	ID	0.953	0.917	0.902
**31**	**JN387133.1**	0.902	0.906	0.898	0.9	0.901	0.899	0.897	0.909	0.907	0.9	0.896	0.895	0.903	0.887	0.899	0.899	0.904	0.9	0.9	0.91	0.9	0.903	0.894	0.92	0.933	0.904	0.895	0.896	0.896	0.953	ID	0.918	0.902
**32**	**KC161964.1**	0.915	0.912	0.91	0.907	0.903	0.909	0.904	0.921	0.923	0.906	0.904	0.903	0.907	0.896	0.913	0.905	0.91	0.909	0.91	0.923	0.915	0.939	0.902	0.926	0.916	0.912	0.914	0.915	0.919	0.917	0.918	ID	0.927
**33**	**KM068048.1**	0.899	0.891	0.892	0.89	0.893	0.895	0.897	0.912	0.913	0.889	0.89	0.89	0.895	0.887	0.896	0.891	0.893	0.896	0.896	0.908	0.901	0.924	0.888	0.912	0.903	0.902	0.916	0.93	0.924	0.902	0.902	0.927	ID

Supplementary Table 9d Deduced amino acid sequence similarities of the CaKoV P1 region

	Sequences	1	2	3	4	5	6	7	8	9	10	11	12	13	14	15	16	17	18	19	20	21	22	23	24	25	26	27	28	29	30	31	32	33
**1**	**PP320363**	ID	0.924	0.926	0.921	0.934	0.927	0.879	0.869	0.866	0.925	0.909	0.909	0.908	0.866	0.941	0.913	0.918	0.927	0.929	0.825	0.813	0.838	0.912	0.822	0.817	0.909	0.815	0.806	0.809	0.822	0.819	0.851	0.826
**2**	**PP320367**	0.924	ID	0.927	0.933	0.903	0.918	0.873	0.861	0.856	0.905	0.891	0.891	0.903	0.859	0.918	0.9	0.891	0.907	0.909	0.817	0.803	0.831	0.891	0.818	0.807	0.891	0.806	0.802	0.797	0.827	0.822	0.84	0.804
**3**	**PP320368**	0.926	0.927	ID	0.927	0.895	0.911	0.873	0.865	0.859	0.907	0.888	0.888	0.908	0.862	0.939	0.908	0.899	0.931	0.938	0.813	0.802	0.824	0.921	0.817	0.81	0.901	0.799	0.792	0.791	0.822	0.805	0.84	0.811
**4**	**PP320362**	0.921	0.933	0.927	ID	0.902	0.92	0.866	0.86	0.861	0.907	0.897	0.897	0.908	0.854	0.918	0.896	0.888	0.911	0.916	0.815	0.803	0.829	0.899	0.815	0.818	0.886	0.803	0.8	0.796	0.819	0.815	0.834	0.803
**5**	**PP320369**	0.934	0.903	0.895	0.902	ID	0.911	0.865	0.853	0.85	0.904	0.894	0.894	0.887	0.848	0.907	0.899	0.902	0.901	0.905	0.809	0.795	0.817	0.885	0.814	0.818	0.887	0.799	0.798	0.788	0.819	0.821	0.828	0.812
**6**	**PP320364**	0.927	0.918	0.911	0.92	0.911	ID	0.887	0.865	0.868	0.905	0.894	0.894	0.9	0.848	0.917	0.909	0.895	0.905	0.91	0.816	0.79	0.82	0.894	0.821	0.818	0.893	0.804	0.797	0.802	0.824	0.815	0.839	0.815
**7**	**PP320365**	0.879	0.873	0.873	0.866	0.865	0.887	ID	0.859	0.852	0.864	0.852	0.852	0.876	0.838	0.875	0.88	0.875	0.871	0.871	0.807	0.78	0.823	0.858	0.806	0.798	0.853	0.791	0.783	0.781	0.817	0.803	0.822	0.814
**8**	**PP320366**	0.869	0.861	0.865	0.86	0.853	0.865	0.859	ID	0.963	0.854	0.851	0.851	0.861	0.818	0.871	0.86	0.86	0.87	0.871	0.832	0.823	0.848	0.848	0.834	0.834	0.859	0.812	0.802	0.807	0.827	0.822	0.853	0.839
**9**	**PP320370**	0.866	0.856	0.859	0.861	0.85	0.868	0.852	0.963	ID	0.853	0.848	0.848	0.865	0.819	0.867	0.855	0.859	0.867	0.87	0.841	0.824	0.856	0.844	0.832	0.841	0.858	0.819	0.804	0.807	0.829	0.825	0.857	0.841
**10**	**PP320361**	0.925	0.905	0.907	0.907	0.904	0.905	0.864	0.854	0.853	ID	0.919	0.919	0.897	0.861	0.913	0.905	0.913	0.913	0.91	0.816	0.794	0.816	0.896	0.818	0.814	0.882	0.805	0.797	0.796	0.819	0.813	0.827	0.799
**11**	**PP320359**	0.909	0.891	0.888	0.897	0.894	0.894	0.852	0.851	0.848	0.919	ID	1	0.882	0.847	0.905	0.891	0.896	0.9	0.896	0.812	0.798	0.821	0.883	0.818	0.81	0.865	0.806	0.799	0.797	0.806	0.804	0.825	0.801
**12**	**PP320360**	0.909	0.891	0.888	0.897	0.894	0.894	0.852	0.851	0.848	0.919	1	ID	0.882	0.847	0.905	0.891	0.896	0.9	0.896	0.812	0.798	0.821	0.883	0.818	0.81	0.865	0.806	0.799	0.797	0.806	0.804	0.825	0.801
**13**	**PP320358**	0.908	0.903	0.908	0.908	0.887	0.9	0.876	0.861	0.865	0.897	0.882	0.882	ID	0.859	0.917	0.92	0.904	0.906	0.913	0.82	0.795	0.841	0.893	0.805	0.807	0.884	0.808	0.8	0.802	0.814	0.813	0.833	0.813
**14**	**OM451182.1**	0.866	0.859	0.862	0.854	0.848	0.848	0.838	0.818	0.819	0.861	0.847	0.847	0.859	ID	0.856	0.86	0.859	0.876	0.871	0.799	0.788	0.82	0.86	0.799	0.774	0.856	0.776	0.781	0.778	0.799	0.785	0.815	0.797
**15**	**MN449341.1**	0.941	0.918	0.939	0.918	0.907	0.917	0.875	0.871	0.867	0.913	0.905	0.905	0.917	0.856	ID	0.913	0.908	0.94	0.942	0.831	0.806	0.83	0.926	0.816	0.815	0.917	0.808	0.804	0.803	0.826	0.81	0.849	0.818
**16**	**MK201779.1**	0.913	0.9	0.908	0.896	0.899	0.909	0.88	0.86	0.855	0.905	0.891	0.891	0.92	0.86	0.913	ID	0.919	0.919	0.917	0.811	0.8	0.833	0.909	0.814	0.813	0.894	0.79	0.787	0.781	0.819	0.814	0.835	0.809
**17**	**MK201778.1**	0.918	0.891	0.899	0.888	0.902	0.895	0.875	0.86	0.859	0.913	0.896	0.896	0.904	0.859	0.908	0.919	ID	0.916	0.916	0.815	0.811	0.827	0.902	0.822	0.815	0.899	0.796	0.788	0.782	0.82	0.815	0.835	0.804
**18**	**MK201776.1**	0.927	0.907	0.931	0.911	0.901	0.905	0.871	0.87	0.867	0.913	0.9	0.9	0.906	0.876	0.94	0.919	0.916	ID	0.988	0.829	0.802	0.836	0.938	0.818	0.81	0.913	0.8	0.799	0.796	0.825	0.812	0.838	0.812
**19**	**MK201777.1**	0.929	0.909	0.938	0.916	0.905	0.91	0.871	0.871	0.87	0.91	0.896	0.896	0.913	0.871	0.942	0.917	0.916	0.988	ID	0.828	0.799	0.836	0.938	0.814	0.812	0.914	0.803	0.799	0.796	0.824	0.809	0.84	0.812
**20**	**MT610361.1**	0.825	0.817	0.813	0.815	0.809	0.816	0.807	0.832	0.841	0.816	0.812	0.812	0.82	0.799	0.831	0.811	0.815	0.829	0.828	ID	0.823	0.842	0.808	0.852	0.828	0.833	0.798	0.798	0.802	0.848	0.826	0.854	0.822
**21**	**MH747478.1**	0.813	0.803	0.802	0.803	0.795	0.79	0.78	0.823	0.824	0.794	0.798	0.798	0.795	0.788	0.806	0.8	0.811	0.802	0.799	0.823	ID	0.839	0.789	0.821	0.799	0.809	0.806	0.8	0.809	0.805	0.798	0.854	0.821
**22**	**MN337880.1**	0.838	0.831	0.824	0.829	0.817	0.82	0.823	0.848	0.856	0.816	0.821	0.821	0.841	0.82	0.83	0.833	0.827	0.836	0.836	0.842	0.839	ID	0.822	0.834	0.821	0.833	0.836	0.845	0.84	0.831	0.821	0.895	0.868
**23**	**MF062158.1**	0.912	0.891	0.921	0.899	0.885	0.894	0.858	0.848	0.844	0.896	0.883	0.883	0.893	0.86	0.926	0.909	0.902	0.938	0.938	0.808	0.789	0.822	ID	0.808	0.809	0.907	0.788	0.787	0.783	0.803	0.792	0.821	0.796
**24**	**KF924623.1**	0.822	0.818	0.817	0.815	0.814	0.821	0.806	0.834	0.832	0.818	0.818	0.818	0.805	0.799	0.816	0.814	0.822	0.818	0.814	0.852	0.821	0.834	0.808	ID	0.845	0.825	0.797	0.799	0.803	0.856	0.849	0.865	0.838
**25**	**MH052678.1**	0.817	0.807	0.81	0.818	0.818	0.818	0.798	0.834	0.841	0.814	0.81	0.81	0.807	0.774	0.815	0.813	0.815	0.81	0.812	0.828	0.799	0.821	0.809	0.845	ID	0.817	0.789	0.787	0.789	0.869	0.855	0.835	0.819
**26**	**JQ911763.1**	0.909	0.891	0.901	0.886	0.887	0.893	0.853	0.859	0.858	0.882	0.865	0.865	0.884	0.856	0.917	0.894	0.899	0.913	0.914	0.833	0.809	0.833	0.907	0.825	0.817	ID	0.803	0.799	0.799	0.836	0.822	0.845	0.824
**27**	**KM068050.1**	0.815	0.806	0.799	0.803	0.799	0.804	0.791	0.812	0.819	0.805	0.806	0.806	0.808	0.776	0.808	0.79	0.796	0.8	0.803	0.798	0.806	0.836	0.788	0.797	0.789	0.803	ID	0.944	0.95	0.788	0.789	0.839	0.84
**28**	**KM068049.1**	0.806	0.802	0.792	0.8	0.798	0.797	0.783	0.802	0.804	0.797	0.799	0.799	0.8	0.781	0.804	0.787	0.788	0.799	0.799	0.798	0.8	0.845	0.787	0.799	0.787	0.799	0.944	ID	0.948	0.784	0.791	0.84	0.863
**29**	**KM068051.1**	0.809	0.797	0.791	0.796	0.788	0.802	0.781	0.807	0.807	0.796	0.797	0.797	0.802	0.778	0.803	0.781	0.782	0.796	0.796	0.802	0.809	0.84	0.783	0.803	0.789	0.799	0.95	0.948	ID	0.781	0.783	0.842	0.856
**30**	**JN088541.1**	0.822	0.827	0.822	0.819	0.819	0.824	0.817	0.827	0.829	0.819	0.806	0.806	0.814	0.799	0.826	0.819	0.82	0.825	0.824	0.848	0.805	0.831	0.803	0.856	0.869	0.836	0.788	0.784	0.781	ID	0.904	0.844	0.822
**31**	**JN387133.1**	0.819	0.822	0.805	0.815	0.821	0.815	0.803	0.822	0.825	0.813	0.804	0.804	0.813	0.785	0.81	0.814	0.815	0.812	0.809	0.826	0.798	0.821	0.792	0.849	0.855	0.822	0.789	0.791	0.783	0.904	ID	0.837	0.816
**32**	**KC161964.1**	0.851	0.84	0.84	0.834	0.828	0.839	0.822	0.853	0.857	0.827	0.825	0.825	0.833	0.815	0.849	0.835	0.835	0.838	0.84	0.854	0.854	0.895	0.821	0.865	0.835	0.845	0.839	0.84	0.842	0.844	0.837	ID	0.867
**33**	**KM068048.1**	0.826	0.804	0.811	0.803	0.812	0.815	0.814	0.839	0.841	0.799	0.801	0.801	0.813	0.797	0.818	0.809	0.804	0.812	0.812	0.822	0.821	0.868	0.796	0.838	0.819	0.824	0.84	0.863	0.856	0.822	0.816	0.867	ID

Supplementary Table 9e Nucleotide sequence similarities of the CaKoV P2 region

	Sequences	1	2	3	4	5	6	7	8	9	10	11	12	13	14	15	16	17	18	19	20	21	22	23	24	25	26	27	28	29	30	31	32	33
**1**	**PP320363**	ID	0.977	0.973	0.973	0.967	0.967	0.967	0.968	0.962	0.973	0.968	0.968	0.971	0.967	0.977	0.959	0.967	0.973	0.974	0.961	0.955	0.949	0.973	0.965	0.965	0.973	0.944	0.938	0.943	0.956	0.953	0.955	0.938
**2**	**PP320367**	0.977	ID	0.985	0.985	0.968	0.973	0.968	0.971	0.965	0.978	0.971	0.971	0.969	0.967	0.974	0.961	0.974	0.971	0.977	0.963	0.956	0.945	0.977	0.97	0.967	0.975	0.944	0.936	0.941	0.957	0.954	0.951	0.937
**3**	**PP320368**	0.973	0.985	ID	0.978	0.963	0.968	0.962	0.961	0.959	0.972	0.965	0.965	0.966	0.959	0.967	0.959	0.965	0.968	0.971	0.957	0.95	0.945	0.969	0.966	0.96	0.971	0.943	0.932	0.938	0.95	0.951	0.949	0.934
**4**	**PP320362**	0.973	0.985	0.978	ID	0.964	0.969	0.962	0.964	0.961	0.972	0.967	0.967	0.969	0.961	0.967	0.958	0.966	0.967	0.971	0.958	0.952	0.944	0.97	0.963	0.959	0.969	0.939	0.932	0.937	0.953	0.951	0.948	0.934
**5**	**PP320369**	0.967	0.968	0.963	0.964	ID	0.964	0.974	0.963	0.965	0.975	0.966	0.966	0.973	0.957	0.971	0.956	0.965	0.971	0.972	0.954	0.951	0.939	0.965	0.959	0.962	0.973	0.943	0.935	0.942	0.957	0.955	0.947	0.936
**6**	**PP320364**	0.967	0.973	0.968	0.969	0.964	ID	0.962	0.963	0.961	0.972	0.963	0.963	0.966	0.959	0.967	0.955	0.963	0.967	0.97	0.958	0.95	0.944	0.967	0.963	0.961	0.97	0.943	0.939	0.942	0.956	0.952	0.951	0.938
**7**	**PP320365**	0.967	0.968	0.962	0.962	0.974	0.962	ID	0.966	0.963	0.972	0.962	0.962	0.968	0.957	0.973	0.956	0.966	0.969	0.97	0.955	0.947	0.938	0.969	0.958	0.958	0.969	0.938	0.931	0.937	0.955	0.953	0.945	0.935
**8**	**PP320366**	0.968	0.971	0.961	0.964	0.963	0.963	0.966	ID	0.956	0.969	0.962	0.962	0.967	0.961	0.973	0.953	0.966	0.971	0.972	0.955	0.949	0.938	0.964	0.963	0.962	0.966	0.939	0.933	0.939	0.954	0.95	0.945	0.932
**9**	**PP320370**	0.962	0.965	0.959	0.961	0.965	0.961	0.963	0.956	ID	0.969	0.959	0.959	0.963	0.954	0.962	0.952	0.959	0.959	0.962	0.951	0.948	0.937	0.963	0.958	0.956	0.962	0.936	0.93	0.934	0.95	0.951	0.944	0.931
**10**	**PP320361**	0.973	0.978	0.972	0.972	0.975	0.972	0.972	0.969	0.969	ID	0.971	0.971	0.976	0.965	0.978	0.965	0.974	0.976	0.983	0.962	0.957	0.947	0.977	0.971	0.965	0.977	0.942	0.937	0.942	0.959	0.956	0.95	0.937
**11**	**PP320359**	0.968	0.971	0.965	0.967	0.966	0.963	0.962	0.962	0.959	0.971	ID	1	0.965	0.958	0.966	0.957	0.963	0.965	0.965	0.959	0.95	0.944	0.963	0.96	0.959	0.966	0.942	0.936	0.942	0.953	0.951	0.952	0.935
**12**	**PP320360**	0.968	0.971	0.965	0.967	0.966	0.963	0.962	0.962	0.959	0.971	1	ID	0.965	0.958	0.966	0.957	0.963	0.965	0.965	0.959	0.95	0.944	0.963	0.96	0.959	0.966	0.942	0.936	0.942	0.953	0.951	0.952	0.935
**13**	**PP320358**	0.971	0.969	0.966	0.969	0.973	0.966	0.968	0.967	0.963	0.976	0.965	0.965	ID	0.96	0.971	0.963	0.965	0.975	0.978	0.955	0.953	0.945	0.966	0.96	0.959	0.971	0.941	0.938	0.941	0.956	0.956	0.948	0.935
**14**	**OM451182.1**	0.967	0.967	0.959	0.961	0.957	0.959	0.957	0.961	0.954	0.965	0.958	0.958	0.96	ID	0.968	0.954	0.96	0.962	0.967	0.953	0.955	0.941	0.963	0.957	0.955	0.967	0.942	0.932	0.939	0.951	0.953	0.945	0.932
**15**	**MN449341.1**	0.977	0.974	0.967	0.967	0.971	0.967	0.973	0.973	0.962	0.978	0.966	0.966	0.971	0.968	ID	0.961	0.975	0.977	0.98	0.96	0.955	0.946	0.976	0.969	0.965	0.977	0.944	0.937	0.941	0.957	0.957	0.953	0.942
**16**	**MK201779.1**	0.959	0.961	0.959	0.958	0.956	0.955	0.956	0.953	0.952	0.965	0.957	0.957	0.963	0.954	0.961	ID	0.96	0.962	0.962	0.948	0.946	0.937	0.965	0.954	0.953	0.962	0.937	0.929	0.934	0.945	0.948	0.947	0.931
**17**	**MK201778.1**	0.967	0.974	0.965	0.966	0.965	0.963	0.966	0.966	0.959	0.974	0.963	0.963	0.965	0.96	0.975	0.96	ID	0.968	0.971	0.96	0.958	0.948	0.971	0.961	0.959	0.974	0.942	0.934	0.941	0.96	0.955	0.953	0.94
**18**	**MK201776.1**	0.973	0.971	0.968	0.967	0.971	0.967	0.969	0.971	0.959	0.976	0.965	0.965	0.975	0.962	0.977	0.962	0.968	ID	0.985	0.957	0.955	0.943	0.971	0.963	0.958	0.972	0.941	0.937	0.942	0.956	0.956	0.951	0.937
**19**	**MK201777.1**	0.974	0.977	0.971	0.971	0.972	0.97	0.97	0.972	0.962	0.983	0.965	0.965	0.978	0.967	0.98	0.962	0.971	0.985	ID	0.96	0.957	0.943	0.975	0.967	0.961	0.973	0.942	0.936	0.941	0.957	0.956	0.95	0.936
**20**	**MT610361.1**	0.961	0.963	0.957	0.958	0.954	0.958	0.955	0.955	0.951	0.962	0.959	0.959	0.955	0.953	0.96	0.948	0.96	0.957	0.96	ID	0.947	0.934	0.959	0.956	0.955	0.961	0.932	0.929	0.931	0.945	0.944	0.94	0.931
**21**	**MH747478.1**	0.955	0.956	0.95	0.952	0.951	0.95	0.947	0.949	0.948	0.957	0.95	0.95	0.953	0.955	0.955	0.946	0.958	0.955	0.957	0.947	ID	0.945	0.956	0.952	0.947	0.959	0.941	0.933	0.938	0.953	0.953	0.947	0.938
**22**	**MN337880.1**	0.949	0.945	0.945	0.944	0.939	0.944	0.938	0.938	0.937	0.947	0.944	0.944	0.945	0.941	0.946	0.937	0.948	0.943	0.943	0.934	0.945	ID	0.941	0.941	0.939	0.947	0.937	0.929	0.935	0.942	0.938	0.948	0.935
**23**	**MF062158.1**	0.973	0.977	0.969	0.97	0.965	0.967	0.969	0.964	0.963	0.977	0.963	0.963	0.966	0.963	0.976	0.965	0.971	0.971	0.975	0.959	0.956	0.941	ID	0.966	0.959	0.974	0.938	0.932	0.937	0.952	0.951	0.945	0.935
**24**	**KF924623.1**	0.965	0.97	0.966	0.963	0.959	0.963	0.958	0.963	0.958	0.971	0.96	0.96	0.96	0.957	0.969	0.954	0.961	0.963	0.967	0.956	0.952	0.941	0.966	ID	0.962	0.968	0.936	0.93	0.936	0.95	0.948	0.943	0.932
**25**	**MH052678.1**	0.965	0.967	0.96	0.959	0.962	0.961	0.958	0.962	0.956	0.965	0.959	0.959	0.959	0.955	0.965	0.953	0.959	0.958	0.961	0.955	0.947	0.939	0.959	0.962	ID	0.963	0.941	0.935	0.942	0.955	0.95	0.948	0.929
**26**	**JQ911763.1**	0.973	0.975	0.971	0.969	0.973	0.97	0.969	0.966	0.962	0.977	0.966	0.966	0.971	0.967	0.977	0.962	0.974	0.972	0.973	0.961	0.959	0.947	0.974	0.968	0.963	ID	0.943	0.941	0.944	0.957	0.958	0.954	0.942
**27**	**KM068050.1**	0.944	0.944	0.943	0.939	0.943	0.943	0.938	0.939	0.936	0.942	0.942	0.942	0.941	0.942	0.944	0.937	0.942	0.941	0.942	0.932	0.941	0.937	0.938	0.936	0.941	0.943	ID	0.974	0.984	0.935	0.936	0.951	0.942
**28**	**KM068049.1**	0.938	0.936	0.932	0.932	0.935	0.939	0.931	0.933	0.93	0.937	0.936	0.936	0.938	0.932	0.937	0.929	0.934	0.937	0.936	0.929	0.933	0.929	0.932	0.93	0.935	0.941	0.974	ID	0.973	0.93	0.93	0.944	0.953
**29**	**KM068051.1**	0.943	0.941	0.938	0.937	0.942	0.942	0.937	0.939	0.934	0.942	0.942	0.942	0.941	0.939	0.941	0.934	0.941	0.942	0.941	0.931	0.938	0.935	0.937	0.936	0.942	0.944	0.984	0.973	ID	0.937	0.933	0.95	0.936
**30**	**JN088541.1**	0.956	0.957	0.95	0.953	0.957	0.956	0.955	0.954	0.95	0.959	0.953	0.953	0.956	0.951	0.957	0.945	0.96	0.956	0.957	0.945	0.953	0.942	0.952	0.95	0.955	0.957	0.935	0.93	0.937	ID	0.969	0.953	0.935
**31**	**JN387133.1**	0.953	0.954	0.951	0.951	0.955	0.952	0.953	0.95	0.951	0.956	0.951	0.951	0.956	0.953	0.957	0.948	0.955	0.956	0.956	0.944	0.953	0.938	0.951	0.948	0.95	0.958	0.936	0.93	0.933	0.969	ID	0.948	0.935
**32**	**KC161964.1**	0.955	0.951	0.949	0.948	0.947	0.951	0.945	0.945	0.944	0.95	0.952	0.952	0.948	0.945	0.953	0.947	0.953	0.951	0.95	0.94	0.947	0.948	0.945	0.943	0.948	0.954	0.951	0.944	0.95	0.953	0.948	ID	0.942
**33**	**KM068048.1**	0.938	0.937	0.934	0.934	0.936	0.938	0.935	0.932	0.931	0.937	0.935	0.935	0.935	0.932	0.942	0.931	0.94	0.937	0.936	0.931	0.938	0.935	0.935	0.932	0.929	0.942	0.942	0.953	0.936	0.935	0.935	0.942	ID

Supplementary Table 9f Deduced amino acid sequence similarities of the CaKoV P2 region

	Sequences	1	2	3	4	5	6	7	8	9	10	11	12	13	14	15	16	17	18	19	20	21	22	23	24	25	26	27	28	29	30	31	32	33
**1**	**PP320363**	ID	1	0.998	1	1	0.998	1	0.998	1	1	1	1	1	0.993	1	1	1	1	1	0.995	1	0.986	1	0.995	0.995	0.998	0.99	0.986	0.993	1	0.995	0.995	0.99
**2**	**PP320367**	1	ID	0.998	1	1	0.998	1	0.998	1	1	1	1	1	0.993	1	1	1	1	1	0.995	1	0.986	1	0.995	0.995	0.998	0.99	0.986	0.993	1	0.995	0.995	0.99
**3**	**PP320368**	0.998	0.998	ID	0.998	0.998	0.996	0.998	0.996	0.998	0.998	0.998	0.998	0.998	0.991	0.998	0.998	0.998	0.998	0.998	0.993	0.998	0.985	0.998	0.993	0.993	0.996	0.988	0.985	0.991	0.998	0.993	0.993	0.988
**4**	**PP320362**	1	1	0.998	ID	1	0.998	1	0.998	1	1	1	1	1	0.993	1	1	1	1	1	0.995	1	0.986	1	0.995	0.995	0.998	0.99	0.986	0.993	1	0.995	0.995	0.99
**5**	**PP320369**	1	1	0.998	1	ID	0.998	1	0.998	1	1	1	1	1	0.993	1	1	1	1	1	0.995	1	0.986	1	0.995	0.995	0.998	0.99	0.986	0.993	1	0.995	0.995	0.99
**6**	**PP320364**	0.998	0.998	0.996	0.998	0.998	ID	0.998	0.996	0.998	0.998	0.998	0.998	0.998	0.991	0.998	0.998	0.998	0.998	0.998	0.993	0.998	0.988	0.998	0.993	0.993	0.996	0.991	0.988	0.995	0.998	0.993	0.996	0.991
**7**	**PP320365**	1	1	0.998	1	1	0.998	ID	0.998	1	1	1	1	1	0.993	1	1	1	1	1	0.995	1	0.986	1	0.995	0.995	0.998	0.99	0.986	0.993	1	0.995	0.995	0.99
**8**	**PP320366**	0.998	0.998	0.996	0.998	0.998	0.996	0.998	ID	0.998	0.998	0.998	0.998	0.998	0.991	0.998	0.998	0.998	0.998	0.998	0.993	0.998	0.985	0.998	0.993	0.993	0.996	0.988	0.986	0.991	0.998	0.993	0.993	0.99
**9**	**PP320370**	1	1	0.998	1	1	0.998	1	0.998	ID	1	1	1	1	0.993	1	1	1	1	1	0.995	1	0.986	1	0.995	0.995	0.998	0.99	0.986	0.993	1	0.995	0.995	0.99
**10**	**PP320361**	1	1	0.998	1	1	0.998	1	0.998	1	ID	1	1	1	0.993	1	1	1	1	1	0.995	1	0.986	1	0.995	0.995	0.998	0.99	0.986	0.993	1	0.995	0.995	0.99
**11**	**PP320359**	1	1	0.998	1	1	0.998	1	0.998	1	1	ID	1	1	0.993	1	1	1	1	1	0.995	1	0.986	1	0.995	0.995	0.998	0.99	0.986	0.993	1	0.995	0.995	0.99
**12**	**PP320360**	1	1	0.998	1	1	0.998	1	0.998	1	1	1	ID	1	0.993	1	1	1	1	1	0.995	1	0.986	1	0.995	0.995	0.998	0.99	0.986	0.993	1	0.995	0.995	0.99
**13**	**PP320358**	1	1	0.998	1	1	0.998	1	0.998	1	1	1	1	ID	0.993	1	1	1	1	1	0.995	1	0.986	1	0.995	0.995	0.998	0.99	0.986	0.993	1	0.995	0.995	0.99
**14**	**OM451182.1**	0.993	0.993	0.991	0.993	0.993	0.991	0.993	0.991	0.993	0.993	0.993	0.993	0.993	ID	0.993	0.993	0.993	0.993	0.993	0.988	0.993	0.985	0.993	0.988	0.988	0.991	0.985	0.981	0.988	0.993	0.988	0.99	0.983
**15**	**MN449341.1**	1	1	0.998	1	1	0.998	1	0.998	1	1	1	1	1	0.993	ID	1	1	1	1	0.995	1	0.986	1	0.995	0.995	0.998	0.99	0.986	0.993	1	0.995	0.995	0.99
**16**	**MK201779.1**	1	1	0.998	1	1	0.998	1	0.998	1	1	1	1	1	0.993	1	ID	1	1	1	0.995	1	0.986	1	0.995	0.995	0.998	0.99	0.986	0.993	1	0.995	0.995	0.99
**17**	**MK201778.1**	1	1	0.998	1	1	0.998	1	0.998	1	1	1	1	1	0.993	1	1	ID	1	1	0.995	1	0.986	1	0.995	0.995	0.998	0.99	0.986	0.993	1	0.995	0.995	0.99
**18**	**MK201776.1**	1	1	0.998	1	1	0.998	1	0.998	1	1	1	1	1	0.993	1	1	1	ID	1	0.995	1	0.986	1	0.995	0.995	0.998	0.99	0.986	0.993	1	0.995	0.995	0.99
**19**	**MK201777.1**	1	1	0.998	1	1	0.998	1	0.998	1	1	1	1	1	0.993	1	1	1	1	ID	0.995	1	0.986	1	0.995	0.995	0.998	0.99	0.986	0.993	1	0.995	0.995	0.99
**20**	**MT610361.1**	0.995	0.995	0.993	0.995	0.995	0.993	0.995	0.993	0.995	0.995	0.995	0.995	0.995	0.988	0.995	0.995	0.995	0.995	0.995	ID	0.995	0.981	0.995	0.99	0.99	0.993	0.985	0.981	0.988	0.995	0.99	0.99	0.985
**21**	**MH747478.1**	1	1	0.998	1	1	0.998	1	0.998	1	1	1	1	1	0.993	1	1	1	1	1	0.995	ID	0.986	1	0.995	0.995	0.998	0.99	0.986	0.993	1	0.995	0.995	0.99
**22**	**MN337880.1**	0.986	0.986	0.985	0.986	0.986	0.988	0.986	0.985	0.986	0.986	0.986	0.986	0.986	0.985	0.986	0.986	0.986	0.986	0.986	0.981	0.986	ID	0.986	0.981	0.985	0.985	0.983	0.98	0.986	0.986	0.981	0.988	0.98
**23**	**MF062158.1**	1	1	0.998	1	1	0.998	1	0.998	1	1	1	1	1	0.993	1	1	1	1	1	0.995	1	0.986	ID	0.995	0.995	0.998	0.99	0.986	0.993	1	0.995	0.995	0.99
**24**	**KF924623.1**	0.995	0.995	0.993	0.995	0.995	0.993	0.995	0.993	0.995	0.995	0.995	0.995	0.995	0.988	0.995	0.995	0.995	0.995	0.995	0.99	0.995	0.981	0.995	ID	0.99	0.993	0.985	0.981	0.988	0.995	0.99	0.99	0.985
**25**	**MH052678.1**	0.995	0.995	0.993	0.995	0.995	0.993	0.995	0.993	0.995	0.995	0.995	0.995	0.995	0.988	0.995	0.995	0.995	0.995	0.995	0.99	0.995	0.985	0.995	0.99	ID	0.993	0.985	0.981	0.988	0.995	0.99	0.99	0.985
**26**	**JQ911763.1**	0.998	0.998	0.996	0.998	0.998	0.996	0.998	0.996	0.998	0.998	0.998	0.998	0.998	0.991	0.998	0.998	0.998	0.998	0.998	0.993	0.998	0.985	0.998	0.993	0.993	ID	0.988	0.985	0.991	0.998	0.993	0.993	0.988
**27**	**KM068050.1**	0.99	0.99	0.988	0.99	0.99	0.991	0.99	0.988	0.99	0.99	0.99	0.99	0.99	0.985	0.99	0.99	0.99	0.99	0.99	0.985	0.99	0.983	0.99	0.985	0.985	0.988	ID	0.993	0.993	0.99	0.985	0.995	0.99
**28**	**KM068049.1**	0.986	0.986	0.985	0.986	0.986	0.988	0.986	0.986	0.986	0.986	0.986	0.986	0.986	0.981	0.986	0.986	0.986	0.986	0.986	0.981	0.986	0.98	0.986	0.981	0.981	0.985	0.993	ID	0.99	0.986	0.981	0.991	0.993
**29**	**KM068051.1**	0.993	0.993	0.991	0.993	0.993	0.995	0.993	0.991	0.993	0.993	0.993	0.993	0.993	0.988	0.993	0.993	0.993	0.993	0.993	0.988	0.993	0.986	0.993	0.988	0.988	0.991	0.993	0.99	ID	0.993	0.988	0.998	0.99
**30**	**JN088541.1**	1	1	0.998	1	1	0.998	1	0.998	1	1	1	1	1	0.993	1	1	1	1	1	0.995	1	0.986	1	0.995	0.995	0.998	0.99	0.986	0.993	ID	0.995	0.995	0.99
**31**	**JN387133.1**	0.995	0.995	0.993	0.995	0.995	0.993	0.995	0.993	0.995	0.995	0.995	0.995	0.995	0.988	0.995	0.995	0.995	0.995	0.995	0.99	0.995	0.981	0.995	0.99	0.99	0.993	0.985	0.981	0.988	0.995	ID	0.99	0.985
**32**	**KC161964.1**	0.995	0.995	0.993	0.995	0.995	0.996	0.995	0.993	0.995	0.995	0.995	0.995	0.995	0.99	0.995	0.995	0.995	0.995	0.995	0.99	0.995	0.988	0.995	0.99	0.99	0.993	0.995	0.991	0.998	0.995	0.99	ID	0.991
**33**	**KM068048.1**	0.99	0.99	0.988	0.99	0.99	0.991	0.99	0.99	0.99	0.99	0.99	0.99	0.99	0.983	0.99	0.99	0.99	0.99	0.99	0.985	0.99	0.98	0.99	0.985	0.985	0.988	0.99	0.993	0.99	0.99	0.985	0.991	ID

Supplementary Table 9g Nucleotide sequence similarities of the CaKoV P3 region

	Sequences	1	2	3	4	5	6	7	8	9	10	11	12	13	14	15	16	17	18	19	20	21	22	23	24	25	26	27	28	29	30	31	32	33
**1**	**PP320363**	ID	0.977	0.979	0.973	0.974	0.969	0.974	0.968	0.971	0.967	0.965	0.965	0.963	0.974	0.974	0.971	0.973	0.975	0.973	0.964	0.951	0.951	0.969	0.96	0.96	0.977	0.945	0.943	0.947	0.952	0.953	0.958	0.948
**2**	**PP320367**	0.977	ID	0.975	0.97	0.98	0.973	0.976	0.969	0.972	0.97	0.967	0.967	0.967	0.974	0.976	0.97	0.973	0.977	0.976	0.965	0.951	0.95	0.97	0.963	0.958	0.979	0.942	0.94	0.944	0.954	0.95	0.957	0.944
**3**	**PP320368**	0.979	0.975	ID	0.971	0.974	0.968	0.97	0.967	0.969	0.964	0.961	0.961	0.959	0.97	0.974	0.972	0.973	0.971	0.971	0.964	0.95	0.948	0.966	0.957	0.959	0.973	0.941	0.941	0.943	0.951	0.948	0.954	0.944
**4**	**PP320362**	0.973	0.97	0.971	ID	0.972	0.966	0.97	0.962	0.965	0.965	0.961	0.961	0.958	0.97	0.972	0.964	0.969	0.97	0.969	0.961	0.944	0.946	0.966	0.957	0.951	0.974	0.942	0.941	0.945	0.949	0.946	0.949	0.945
**5**	**PP320369**	0.974	0.98	0.974	0.972	ID	0.971	0.976	0.967	0.972	0.97	0.966	0.966	0.961	0.979	0.976	0.972	0.972	0.979	0.977	0.966	0.95	0.951	0.972	0.962	0.964	0.982	0.95	0.946	0.951	0.956	0.955	0.959	0.949
**6**	**PP320364**	0.969	0.973	0.968	0.966	0.971	ID	0.974	0.972	0.969	0.967	0.963	0.963	0.965	0.97	0.974	0.971	0.973	0.973	0.972	0.966	0.949	0.948	0.967	0.956	0.96	0.976	0.939	0.938	0.941	0.95	0.945	0.952	0.942
**7**	**PP320365**	0.974	0.976	0.97	0.97	0.976	0.974	ID	0.969	0.97	0.97	0.965	0.965	0.962	0.971	0.973	0.967	0.97	0.976	0.973	0.964	0.951	0.949	0.971	0.961	0.959	0.979	0.943	0.943	0.944	0.951	0.948	0.952	0.946
**8**	**PP320366**	0.968	0.969	0.967	0.962	0.967	0.972	0.969	ID	0.966	0.961	0.957	0.957	0.96	0.968	0.97	0.965	0.97	0.97	0.967	0.961	0.948	0.946	0.964	0.954	0.954	0.97	0.938	0.938	0.939	0.949	0.945	0.951	0.941
**9**	**PP320370**	0.971	0.972	0.969	0.965	0.972	0.969	0.97	0.966	ID	0.964	0.962	0.962	0.964	0.976	0.968	0.968	0.97	0.974	0.973	0.965	0.948	0.945	0.97	0.959	0.959	0.974	0.941	0.938	0.943	0.949	0.948	0.951	0.941
**10**	**PP320361**	0.967	0.97	0.964	0.965	0.97	0.967	0.97	0.961	0.964	ID	0.977	0.977	0.964	0.968	0.968	0.967	0.966	0.969	0.967	0.961	0.948	0.95	0.964	0.957	0.959	0.972	0.944	0.942	0.947	0.953	0.948	0.955	0.947
**11**	**PP320359**	0.965	0.967	0.961	0.961	0.966	0.963	0.965	0.957	0.962	0.977	ID	1	0.961	0.963	0.969	0.965	0.964	0.967	0.964	0.956	0.943	0.945	0.961	0.956	0.955	0.969	0.942	0.939	0.944	0.95	0.945	0.949	0.943
**12**	**PP320360**	0.965	0.967	0.961	0.961	0.966	0.963	0.965	0.957	0.962	0.977	1	ID	0.961	0.963	0.969	0.965	0.964	0.967	0.964	0.956	0.943	0.945	0.961	0.956	0.955	0.969	0.942	0.939	0.944	0.95	0.945	0.949	0.943
**13**	**PP320358**	0.963	0.967	0.959	0.958	0.961	0.965	0.962	0.96	0.964	0.964	0.961	0.961	ID	0.964	0.965	0.963	0.964	0.966	0.963	0.957	0.949	0.945	0.957	0.95	0.953	0.967	0.938	0.937	0.941	0.945	0.944	0.945	0.939
**14**	**OM451182.1**	0.974	0.974	0.97	0.97	0.979	0.97	0.971	0.968	0.976	0.968	0.963	0.963	0.964	ID	0.976	0.969	0.971	0.978	0.976	0.966	0.95	0.948	0.97	0.961	0.962	0.979	0.946	0.944	0.948	0.955	0.952	0.958	0.946
**15**	**MN449341.1**	0.974	0.976	0.974	0.972	0.976	0.974	0.973	0.97	0.968	0.968	0.969	0.969	0.965	0.976	ID	0.978	0.978	0.976	0.974	0.967	0.948	0.951	0.969	0.961	0.96	0.981	0.942	0.939	0.944	0.951	0.95	0.955	0.942
**16**	**MK201779.1**	0.971	0.97	0.972	0.964	0.972	0.971	0.967	0.965	0.968	0.967	0.965	0.965	0.963	0.969	0.978	ID	0.988	0.973	0.971	0.963	0.946	0.949	0.964	0.957	0.96	0.974	0.939	0.936	0.942	0.949	0.95	0.955	0.94
**17**	**MK201778.1**	0.973	0.973	0.973	0.969	0.972	0.973	0.97	0.97	0.97	0.966	0.964	0.964	0.964	0.971	0.978	0.988	ID	0.976	0.974	0.966	0.948	0.949	0.966	0.96	0.96	0.976	0.942	0.94	0.944	0.95	0.949	0.955	0.942
**18**	**MK201776.1**	0.975	0.977	0.971	0.97	0.979	0.973	0.976	0.97	0.974	0.969	0.967	0.967	0.966	0.978	0.976	0.973	0.976	ID	0.995	0.97	0.953	0.948	0.973	0.963	0.962	0.983	0.942	0.941	0.944	0.952	0.952	0.957	0.946
**19**	**MK201777.1**	0.973	0.976	0.971	0.969	0.977	0.972	0.973	0.967	0.973	0.967	0.964	0.964	0.963	0.976	0.974	0.971	0.974	0.995	ID	0.969	0.952	0.947	0.971	0.96	0.96	0.98	0.941	0.94	0.943	0.951	0.95	0.957	0.944
**20**	**MT610361.1**	0.964	0.965	0.964	0.961	0.966	0.966	0.964	0.961	0.965	0.961	0.956	0.956	0.957	0.966	0.967	0.963	0.966	0.97	0.969	ID	0.948	0.948	0.961	0.955	0.957	0.971	0.94	0.939	0.942	0.948	0.945	0.952	0.943
**21**	**MH747478.1**	0.951	0.951	0.95	0.944	0.95	0.949	0.951	0.948	0.948	0.948	0.943	0.943	0.949	0.95	0.948	0.946	0.948	0.953	0.952	0.948	ID	0.949	0.948	0.941	0.942	0.951	0.939	0.939	0.94	0.951	0.946	0.95	0.943
**22**	**MN337880.1**	0.951	0.95	0.948	0.946	0.951	0.948	0.949	0.946	0.945	0.95	0.945	0.945	0.945	0.948	0.951	0.949	0.949	0.948	0.947	0.948	0.949	ID	0.946	0.935	0.941	0.948	0.939	0.937	0.939	0.951	0.948	0.953	0.943
**23**	**MF062158.1**	0.969	0.97	0.966	0.966	0.972	0.967	0.971	0.964	0.97	0.964	0.961	0.961	0.957	0.97	0.969	0.964	0.966	0.973	0.971	0.961	0.948	0.946	ID	0.961	0.956	0.975	0.941	0.938	0.941	0.95	0.949	0.954	0.941
**24**	**KF924623.1**	0.96	0.963	0.957	0.957	0.962	0.956	0.961	0.954	0.959	0.957	0.956	0.956	0.95	0.961	0.961	0.957	0.96	0.963	0.96	0.955	0.941	0.935	0.961	ID	0.952	0.967	0.932	0.931	0.935	0.94	0.936	0.942	0.933
**25**	**MH052678.1**	0.96	0.958	0.959	0.951	0.964	0.96	0.959	0.954	0.959	0.959	0.955	0.955	0.953	0.962	0.96	0.96	0.96	0.962	0.96	0.957	0.942	0.941	0.956	0.952	ID	0.968	0.937	0.936	0.941	0.947	0.944	0.946	0.94
**26**	**JQ911763.1**	0.977	0.979	0.973	0.974	0.982	0.976	0.979	0.97	0.974	0.972	0.969	0.969	0.967	0.979	0.981	0.974	0.976	0.983	0.98	0.971	0.951	0.948	0.975	0.967	0.968	ID	0.946	0.943	0.947	0.955	0.953	0.957	0.946
**27**	**KM068050.1**	0.945	0.942	0.941	0.942	0.95	0.939	0.943	0.938	0.941	0.944	0.942	0.942	0.938	0.946	0.942	0.939	0.942	0.942	0.941	0.94	0.939	0.939	0.941	0.932	0.937	0.946	ID	0.985	0.99	0.946	0.947	0.943	0.953
**28**	**KM068049.1**	0.943	0.94	0.941	0.941	0.946	0.938	0.943	0.938	0.938	0.942	0.939	0.939	0.937	0.944	0.939	0.936	0.94	0.941	0.94	0.939	0.939	0.937	0.938	0.931	0.936	0.943	0.985	ID	0.982	0.945	0.945	0.938	0.962
**29**	**KM068051.1**	0.947	0.944	0.943	0.945	0.951	0.941	0.944	0.939	0.943	0.947	0.944	0.944	0.941	0.948	0.944	0.942	0.944	0.944	0.943	0.942	0.94	0.939	0.941	0.935	0.941	0.947	0.99	0.982	ID	0.948	0.948	0.944	0.956
**30**	**JN088541.1**	0.952	0.954	0.951	0.949	0.956	0.95	0.951	0.949	0.949	0.953	0.95	0.95	0.945	0.955	0.951	0.949	0.95	0.952	0.951	0.948	0.951	0.951	0.95	0.94	0.947	0.955	0.946	0.945	0.947	ID	0.967	0.953	0.951
**31**	**JN387133.1**	0.953	0.95	0.948	0.946	0.955	0.945	0.948	0.945	0.948	0.948	0.945	0.945	0.944	0.952	0.95	0.95	0.949	0.952	0.95	0.945	0.946	0.948	0.949	0.936	0.944	0.953	0.947	0.945	0.948	0.967	ID	0.953	0.951
**32**	**KC161964.1**	0.958	0.957	0.954	0.949	0.959	0.952	0.952	0.951	0.951	0.955	0.949	0.949	0.945	0.958	0.955	0.955	0.955	0.957	0.957	0.952	0.95	0.953	0.954	0.942	0.946	0.957	0.943	0.938	0.944	0.953	0.953	ID	0.944
**33**	**KM068048.1**	0.948	0.944	0.944	0.945	0.949	0.942	0.946	0.941	0.941	0.947	0.943	0.943	0.939	0.946	0.942	0.94	0.942	0.946	0.944	0.943	0.943	0.943	0.941	0.933	0.94	0.946	0.953	0.962	0.956	0.951	0.951	0.944	ID

Supplementary Table 9h Deduced amino acid sequence similarities of the CaKoV P3 region

	Sequences	1	2	3	4	5	6	7	8	9	10	11	12	13	14	15	16	17	18	19	20	21	22	23	24	25	26	27	28	29	30	31	32	33
**1**	**PP320363**	ID	0.998	0.993	0.997	0.997	0.997	0.998	0.992	0.994	0.997	0.993	0.993	0.996	0.994	0.994	0.994	0.997	1	1	0.991	0.987	0.985	0.996	0.985	0.992	0.998	0.988	0.985	0.991	0.991	0.984	0.993	0.992
**2**	**PP320367**	0.998	ID	0.992	0.996	0.996	0.996	0.997	0.991	0.993	0.996	0.992	0.992	0.994	0.993	0.993	0.993	0.996	0.998	0.998	0.989	0.985	0.984	0.994	0.984	0.991	0.997	0.987	0.984	0.989	0.989	0.983	0.992	0.991
**3**	**PP320368**	0.993	0.992	ID	0.993	0.992	0.991	0.992	0.985	0.991	0.991	0.991	0.991	0.989	0.988	0.991	0.992	0.992	0.993	0.993	0.985	0.98	0.979	0.989	0.983	0.988	0.992	0.982	0.979	0.984	0.984	0.978	0.988	0.985
**4**	**PP320362**	0.997	0.996	0.993	ID	0.994	0.994	0.996	0.989	0.992	0.994	0.992	0.992	0.993	0.992	0.994	0.993	0.996	0.997	0.997	0.989	0.984	0.983	0.993	0.984	0.989	0.996	0.985	0.983	0.988	0.988	0.982	0.992	0.989
**5**	**PP320369**	0.997	0.996	0.992	0.994	ID	0.994	0.996	0.989	0.992	0.994	0.991	0.991	0.993	0.992	0.992	0.992	0.994	0.997	0.997	0.988	0.984	0.985	0.993	0.983	0.989	0.996	0.985	0.983	0.988	0.988	0.982	0.991	0.989
**6**	**PP320364**	0.997	0.996	0.991	0.994	0.994	ID	0.996	0.994	0.994	0.997	0.993	0.993	0.996	0.994	0.997	0.994	0.997	0.997	0.997	0.993	0.987	0.988	0.993	0.985	0.992	0.996	0.988	0.985	0.991	0.991	0.984	0.994	0.992
**7**	**PP320365**	0.998	0.997	0.992	0.996	0.996	0.996	ID	0.991	0.993	0.996	0.992	0.992	0.994	0.993	0.993	0.993	0.996	0.998	0.998	0.989	0.985	0.984	0.994	0.984	0.991	0.997	0.987	0.984	0.989	0.989	0.983	0.992	0.991
**8**	**PP320366**	0.992	0.991	0.985	0.989	0.989	0.994	0.991	ID	0.989	0.992	0.988	0.988	0.991	0.989	0.992	0.989	0.992	0.992	0.992	0.988	0.982	0.985	0.988	0.98	0.988	0.991	0.983	0.98	0.985	0.985	0.979	0.989	0.987
**9**	**PP320370**	0.994	0.993	0.991	0.992	0.992	0.994	0.993	0.989	ID	0.994	0.993	0.993	0.993	0.992	0.992	0.994	0.994	0.994	0.994	0.988	0.984	0.983	0.991	0.985	0.994	0.993	0.985	0.983	0.988	0.988	0.982	0.989	0.989
**10**	**PP320361**	0.997	0.996	0.991	0.994	0.994	0.997	0.996	0.992	0.994	ID	0.993	0.993	0.996	0.994	0.994	0.994	0.997	0.997	0.997	0.991	0.989	0.988	0.993	0.985	0.992	0.996	0.991	0.988	0.992	0.993	0.987	0.992	0.994
**11**	**PP320359**	0.993	0.992	0.991	0.992	0.991	0.993	0.992	0.988	0.993	0.993	ID	1	0.992	0.991	0.992	0.994	0.994	0.993	0.993	0.989	0.983	0.982	0.989	0.987	0.991	0.992	0.984	0.982	0.987	0.987	0.98	0.989	0.988
**12**	**PP320360**	0.993	0.992	0.991	0.992	0.991	0.993	0.992	0.988	0.993	0.993	1	ID	0.992	0.991	0.992	0.994	0.994	0.993	0.993	0.989	0.983	0.982	0.989	0.987	0.991	0.992	0.984	0.982	0.987	0.987	0.98	0.989	0.988
**13**	**PP320358**	0.996	0.994	0.989	0.993	0.993	0.996	0.994	0.991	0.993	0.996	0.992	0.992	ID	0.993	0.993	0.993	0.996	0.996	0.996	0.989	0.985	0.984	0.992	0.984	0.991	0.994	0.987	0.984	0.989	0.989	0.983	0.991	0.991
**14**	**OM451182.1**	0.994	0.993	0.988	0.992	0.992	0.994	0.993	0.989	0.992	0.994	0.991	0.991	0.993	ID	0.992	0.992	0.994	0.994	0.994	0.988	0.984	0.983	0.991	0.983	0.989	0.993	0.985	0.983	0.988	0.988	0.982	0.989	0.989
**15**	**MN449341.1**	0.994	0.993	0.991	0.994	0.992	0.997	0.993	0.992	0.992	0.994	0.992	0.992	0.993	0.992	ID	0.993	0.996	0.994	0.994	0.992	0.984	0.985	0.991	0.984	0.989	0.993	0.985	0.983	0.988	0.988	0.982	0.993	0.989
**16**	**MK201779.1**	0.994	0.993	0.992	0.993	0.992	0.994	0.993	0.989	0.994	0.994	0.994	0.994	0.993	0.992	0.993	ID	0.997	0.994	0.994	0.989	0.984	0.983	0.991	0.987	0.992	0.993	0.985	0.983	0.988	0.988	0.982	0.992	0.989
**17**	**MK201778.1**	0.997	0.996	0.992	0.996	0.994	0.997	0.996	0.992	0.994	0.997	0.994	0.994	0.996	0.994	0.996	0.997	ID	0.997	0.997	0.992	0.987	0.985	0.993	0.987	0.992	0.996	0.988	0.985	0.991	0.991	0.984	0.994	0.992
**18**	**MK201776.1**	1	0.998	0.993	0.997	0.997	0.997	0.998	0.992	0.994	0.997	0.993	0.993	0.996	0.994	0.994	0.994	0.997	ID	1	0.991	0.987	0.985	0.996	0.985	0.992	0.998	0.988	0.985	0.991	0.991	0.984	0.993	0.992
**19**	**MK201777.1**	1	0.998	0.993	0.997	0.997	0.997	0.998	0.992	0.994	0.997	0.993	0.993	0.996	0.994	0.994	0.994	0.997	1	ID	0.991	0.987	0.985	0.996	0.985	0.992	0.998	0.988	0.985	0.991	0.991	0.984	0.993	0.992
**20**	**MT610361.1**	0.991	0.989	0.985	0.989	0.988	0.993	0.989	0.988	0.988	0.991	0.989	0.989	0.989	0.988	0.992	0.989	0.992	0.991	0.991	ID	0.984	0.983	0.987	0.984	0.988	0.992	0.983	0.98	0.985	0.988	0.982	0.993	0.989
**21**	**MH747478.1**	0.987	0.985	0.98	0.984	0.984	0.987	0.985	0.982	0.984	0.989	0.983	0.983	0.985	0.984	0.984	0.984	0.987	0.987	0.987	0.984	ID	0.98	0.983	0.978	0.984	0.988	0.983	0.98	0.984	0.991	0.984	0.987	0.989
**22**	**MN337880.1**	0.985	0.984	0.979	0.983	0.985	0.988	0.984	0.985	0.983	0.988	0.982	0.982	0.984	0.983	0.985	0.983	0.985	0.985	0.985	0.983	0.98	ID	0.982	0.974	0.98	0.984	0.982	0.979	0.983	0.984	0.978	0.985	0.985
**23**	**MF062158.1**	0.996	0.994	0.989	0.993	0.993	0.993	0.994	0.988	0.991	0.993	0.989	0.989	0.992	0.991	0.991	0.991	0.993	0.996	0.996	0.987	0.983	0.982	ID	0.982	0.988	0.994	0.984	0.982	0.987	0.987	0.98	0.989	0.988
**24**	**KF924623.1**	0.985	0.984	0.983	0.984	0.983	0.985	0.984	0.98	0.985	0.985	0.987	0.987	0.984	0.983	0.984	0.987	0.987	0.985	0.985	0.984	0.978	0.974	0.982	ID	0.985	0.987	0.976	0.974	0.979	0.982	0.975	0.984	0.983
**25**	**MH052678.1**	0.992	0.991	0.988	0.989	0.989	0.992	0.991	0.988	0.994	0.992	0.991	0.991	0.991	0.989	0.989	0.992	0.992	0.992	0.992	0.988	0.984	0.98	0.988	0.985	ID	0.993	0.983	0.98	0.985	0.988	0.982	0.989	0.989
**26**	**JQ911763.1**	0.998	0.997	0.992	0.996	0.996	0.996	0.997	0.991	0.993	0.996	0.992	0.992	0.994	0.993	0.993	0.993	0.996	0.998	0.998	0.992	0.988	0.984	0.994	0.987	0.993	ID	0.987	0.984	0.989	0.992	0.985	0.994	0.993
**27**	**KM068050.1**	0.988	0.987	0.982	0.985	0.985	0.988	0.987	0.983	0.985	0.991	0.984	0.984	0.987	0.985	0.985	0.985	0.988	0.988	0.988	0.983	0.983	0.982	0.984	0.976	0.983	0.987	ID	0.989	0.996	0.987	0.98	0.985	0.988
**28**	**KM068049.1**	0.985	0.984	0.979	0.983	0.983	0.985	0.984	0.98	0.983	0.988	0.982	0.982	0.984	0.983	0.983	0.983	0.985	0.985	0.985	0.98	0.98	0.979	0.982	0.974	0.98	0.984	0.989	ID	0.991	0.984	0.978	0.983	0.985
**29**	**KM068051.1**	0.991	0.989	0.984	0.988	0.988	0.991	0.989	0.985	0.988	0.992	0.987	0.987	0.989	0.988	0.988	0.988	0.991	0.991	0.991	0.985	0.984	0.983	0.987	0.979	0.985	0.989	0.996	0.991	ID	0.988	0.982	0.988	0.989
**30**	**JN088541.1**	0.991	0.989	0.984	0.988	0.988	0.991	0.989	0.985	0.988	0.993	0.987	0.987	0.989	0.988	0.988	0.988	0.991	0.991	0.991	0.988	0.991	0.984	0.987	0.982	0.988	0.992	0.987	0.984	0.988	ID	0.991	0.991	0.994
**31**	**JN387133.1**	0.984	0.983	0.978	0.982	0.982	0.984	0.983	0.979	0.982	0.987	0.98	0.98	0.983	0.982	0.982	0.982	0.984	0.984	0.984	0.982	0.984	0.978	0.98	0.975	0.982	0.985	0.98	0.978	0.982	0.991	ID	0.984	0.987
**32**	**KC161964.1**	0.993	0.992	0.988	0.992	0.991	0.994	0.992	0.989	0.989	0.992	0.989	0.989	0.991	0.989	0.993	0.992	0.994	0.993	0.993	0.993	0.987	0.985	0.989	0.984	0.989	0.994	0.985	0.983	0.988	0.991	0.984	ID	0.992
**33**	**KM068048.1**	0.992	0.991	0.985	0.989	0.989	0.992	0.991	0.987	0.989	0.994	0.988	0.988	0.991	0.989	0.989	0.989	0.992	0.992	0.992	0.989	0.989	0.985	0.988	0.983	0.989	0.993	0.988	0.985	0.989	0.994	0.987	0.992	ID

Supplementary Table 9i Nucleotide sequence similarities of the CaKoV VP1 gene

	Sequences	1	2	3	4	5	6	7	8	9	10	11	12	13	14	15	16	17	18	19	20	21	22	23	24	25	26	27	28	29	30	31	32	33
**1**	**PP320363**	ID	0.946	0.948	0.944	0.976	0.958	0.904	0.87	0.868	0.954	0.949	0.949	0.94	0.919	0.95	0.943	0.949	0.949	0.954	0.851	0.829	0.852	0.947	0.865	0.871	0.907	0.85	0.846	0.854	0.857	0.864	0.87	0.854
**2**	**PP320367**	0.946	ID	0.932	0.936	0.941	0.94	0.899	0.87	0.865	0.93	0.931	0.931	0.925	0.906	0.929	0.926	0.925	0.929	0.929	0.849	0.829	0.856	0.922	0.862	0.858	0.885	0.839	0.838	0.844	0.861	0.865	0.862	0.839
**3**	**PP320368**	0.948	0.932	ID	0.942	0.936	0.935	0.901	0.869	0.866	0.938	0.935	0.935	0.935	0.913	0.962	0.937	0.936	0.96	0.965	0.84	0.826	0.85	0.959	0.859	0.866	0.9	0.833	0.832	0.841	0.852	0.851	0.869	0.847
**4**	**PP320362**	0.944	0.936	0.942	ID	0.94	0.944	0.89	0.862	0.862	0.94	0.935	0.935	0.936	0.91	0.94	0.931	0.931	0.94	0.944	0.844	0.828	0.851	0.937	0.859	0.859	0.884	0.828	0.827	0.835	0.857	0.854	0.86	0.838
**5**	**PP320369**	0.976	0.941	0.936	0.94	ID	0.953	0.902	0.872	0.872	0.948	0.948	0.948	0.935	0.91	0.941	0.938	0.943	0.937	0.942	0.848	0.817	0.851	0.937	0.866	0.869	0.901	0.838	0.834	0.842	0.855	0.864	0.859	0.847
**6**	**PP320364**	0.958	0.94	0.935	0.944	0.953	ID	0.905	0.869	0.874	0.948	0.941	0.941	0.94	0.904	0.94	0.944	0.935	0.941	0.946	0.854	0.824	0.845	0.937	0.868	0.871	0.89	0.834	0.833	0.844	0.867	0.864	0.869	0.844
**7**	**PP320365**	0.904	0.899	0.901	0.89	0.902	0.905	ID	0.868	0.868	0.892	0.894	0.894	0.907	0.9	0.895	0.908	0.895	0.896	0.899	0.84	0.816	0.857	0.894	0.841	0.854	0.851	0.832	0.822	0.83	0.846	0.85	0.852	0.846
**8**	**PP320366**	0.87	0.87	0.869	0.862	0.872	0.869	0.868	ID	0.979	0.868	0.869	0.869	0.869	0.853	0.868	0.868	0.875	0.87	0.871	0.882	0.869	0.906	0.863	0.898	0.901	0.857	0.875	0.872	0.884	0.88	0.884	0.908	0.9
**9**	**PP320370**	0.868	0.865	0.866		0.872	0.874	0.868	0.979	ID	0.868	0.869	0.869	0.871	0.848	0.864	0.865	0.875	0.863	0.866	0.883	0.872	0.905	0.86	0.9	0.907	0.854	0.878	0.871	0.883	0.881	0.883	0.912	0.898
**10**	**PP320361**	0.954	0.93	0.938	0.94	0.948	0.948	0.892	0.868	0.868	ID	0.947	0.947	0.936	0.91	0.938	0.941	0.953	0.947	0.949	0.843	0.821	0.844	0.938	0.864	0.86	0.889	0.841	0.841	0.85	0.851	0.86	0.856	0.841
**11**	**PP320359**	0.949	0.931	0.935	0.935	0.948	0.941	0.894	0.869	0.869	0.947	ID	1	0.917	0.905	0.935	0.924	0.926	0.936	0.938	0.843	0.823	0.851	0.932	0.857	0.858	0.879	0.834	0.832	0.84	0.845	0.845	0.862	0.841
**12**	**PP320360**	0.949	0.931	0.935	0.935	0.948	0.941	0.894	0.869	0.869	0.947	1	ID	0.917	0.905	0.935	0.924	0.926	0.936	0.938	0.843	0.823	0.851	0.932	0.857	0.858	0.879	0.834	0.832	0.84	0.845	0.845	0.862	0.841
**13**	**PP320358**	0.94	0.925	0.935	0.936	0.935	0.94	0.907	0.869	0.871	0.936	0.917	0.917	ID	0.916	0.944	0.96	0.946	0.941	0.946	0.852	0.827	0.859	0.94	0.856	0.857	0.889	0.844	0.84	0.848	0.852	0.858	0.864	0.848
**14**	**OM451182.1**	0.919	0.906	0.913	0.91	0.91	0.904	0.9	0.853	0.848	0.91	0.905	0.905	0.916	ID	0.902	0.913	0.917	0.922	0.919	0.852	0.84	0.866	0.913	0.863	0.845	0.882	0.841	0.84	0.852	0.855	0.851	0.862	0.859
**15**	**MN449341.1**	0.95	0.929	0.962	0.94	0.941	0.94	0.895	0.868	0.864	0.938	0.935	0.935	0.944	0.902	ID	0.944	0.936	0.964	0.965	0.849	0.823	0.844	0.959	0.854	0.862	0.903	0.832	0.832	0.84	0.849	0.85	0.862	0.839
**16**	**MK201779.1**	0.943	0.926	0.937	0.931	0.938	0.944	0.908	0.868	0.865	0.941	0.924	0.924	0.96	0.913	0.944	ID	0.946	0.943	0.948	0.843	0.821	0.85	0.944	0.85	0.856	0.891	0.829	0.826	0.834	0.849	0.852	0.857	0.84
**17**	**MK201778.1**	0.949	0.925	0.936	0.931	0.943	0.935	0.895	0.875	0.875	0.953	0.926	0.926	0.946	0.917	0.936	0.946	ID	0.937	0.942	0.851	0.835	0.853	0.941	0.865	0.864	0.9	0.834	0.83	0.836	0.855	0.868	0.863	0.844
**18**	**MK201776.1**	0.949	0.929	0.96	0.94	0.937	0.941	0.896	0.87	0.863	0.947	0.936	0.936	0.941	0.922	0.964	0.943	0.937	ID	0.995	0.849	0.827	0.85	0.962	0.858	0.852	0.901	0.834	0.836	0.845	0.852	0.854	0.856	0.841
**19**	**MK201777.1**	0.954	0.929	0.965	0.944	0.942	0.946	0.899	0.871	0.866	0.949	0.938	0.938	0.946	0.919	0.965	0.948	0.942	0.995	ID	0.85	0.828	0.851	0.965	0.854	0.856	0.902	0.838	0.838	0.846	0.854	0.856	0.859	0.842
**20**	**MT610361.1**	0.851	0.849	0.84	0.844	0.848	0.854	0.84	0.882	0.883	0.843	0.843	0.843	0.852	0.852	0.849	0.843	0.851	0.849	0.85	ID	0.872	0.894	0.838	0.919	0.898	0.87	0.87	0.87	0.882	0.917	0.899	0.906	0.891
**21**	**MH747478.1**	0.829	0.829	0.826	0.828	0.817	0.824	0.816	0.869	0.872	0.821	0.823	0.823	0.827	0.84	0.823	0.821	0.835	0.827	0.828	0.872	ID	0.887	0.822	0.884	0.872	0.846	0.874	0.869	0.878	0.875	0.857	0.911	0.889
**22**	**MN337880.1**	0.852	0.856	0.85	0.851	0.851	0.845	0.857	0.906	0.905	0.844	0.851	0.851	0.859	0.866	0.844	0.85	0.853	0.85	0.851	0.894	0.887	ID	0.842	0.901	0.888	0.853	0.905	0.902	0.916	0.885	0.882	0.926	0.924
**23**	**MF062158.1**	0.947	0.922	0.959	0.937	0.937	0.937	0.894	0.863	0.86	0.938	0.932	0.932	0.94	0.913	0.959	0.944	0.941	0.962	0.965	0.838	0.822	0.842	ID	0.854	0.86	0.898	0.828	0.827	0.835	0.842	0.846	0.848	0.834
**24**	**KF924623.1**	0.865	0.862	0.859	0.859	0.866	0.868	0.841	0.898	0.9	0.864	0.857	0.857	0.856	0.863	0.854	0.85	0.865	0.858	0.854	0.919	0.884	0.901	0.854	ID	0.93	0.867	0.88	0.88	0.895	0.922	0.916	0.929	0.91
**25**	**MH052678.1**	0.871	0.858	0.866	0.859	0.869	0.871	0.854	0.901	0.907	0.86	0.858	0.858	0.857	0.845	0.862	0.856	0.864	0.852	0.856	0.898	0.872	0.888	0.86	0.93	ID	0.865	0.871	0.866	0.881	0.929	0.92	0.918	0.899
**26**	**JQ911763.1**	0.907	0.885	0.9	0.884	0.901	0.89	0.851	0.857	0.854	0.889	0.879	0.879	0.889	0.882	0.903	0.891	0.9	0.901	0.902	0.87	0.846	0.853	0.898	0.867	0.865	ID	0.857	0.855	0.85	0.874	0.865	0.869	0.854
**27**	**KM068050.1**	0.85	0.839	0.833	0.828	0.838	0.834	0.832	0.875	0.878	0.841	0.834	0.834	0.844	0.841	0.832	0.829	0.834	0.834	0.838	0.87	0.874	0.905	0.828	0.88	0.871	0.857	ID	0.984	0.968	0.866	0.869	0.908	0.907
**28**	**KM068049.1**	0.846	0.838	0.832	0.827	0.834	0.833	0.822	0.872	0.871	0.841	0.832	0.832	0.84	0.84	0.832	0.826	0.83	0.836	0.838	0.87	0.869	0.902	0.827	0.88	0.866	0.855	0.984	ID	0.979	0.863	0.866	0.906	0.907
**29**	**KM068051.1**	0.854	0.844	0.841	0.835	0.842	0.844	0.83	0.884	0.883	0.85	0.84	0.84	0.848	0.852	0.84	0.834	0.836	0.845	0.846	0.882	0.878	0.916	0.835	0.895	0.881	0.85	0.968	0.979	ID	0.874	0.875	0.923	0.928
**30**	**JN088541.1**	0.857	0.861	0.852	0.857	0.855	0.867	0.846	0.88	0.881	0.851	0.845	0.845	0.852	0.855	0.849	0.849	0.855	0.852	0.854	0.917	0.875	0.885	0.842	0.922	0.929	0.874	0.866	0.863	0.874	ID	0.948	0.909	0.885
**31**	**JN387133.1**	0.864	0.865	0.851	0.854	0.864	0.864	0.85	0.884	0.883	0.86	0.845	0.845	0.858	0.851	0.85	0.852	0.868	0.854	0.856	0.899	0.857	0.882	0.846	0.916	0.92	0.865	0.869	0.866	0.875	0.948	ID	0.904	0.892
**32**	**KC161964.1**	0.87	0.862	0.869	0.86	0.859	0.869	0.852	0.908	0.912	0.856	0.862	0.862	0.864	0.862	0.862	0.857	0.863	0.856	0.859	0.906	0.911	0.926	0.848	0.929	0.918	0.869	0.908	0.906	0.923	0.909	0.904	ID	0.93
**33**	**KM068048.1**	0.854	0.839	0.847	0.838	0.847	0.844	0.846	0.9	0.898	0.841	0.841	0.841	0.848	0.859	0.839	0.84	0.844	0.841	0.842	0.891	0.889	0.924	0.834	0.91	0.899	0.854	0.907	0.907	0.928	0.885	0.892	0.93	ID

Supplementary Table 9k Deduced amino acid sequence similarities of the CaKoV VP1 gene

	Sequences	1	2	3	4	5	6	7	8	9	10	11	12	13	14	15	16	17	18	19	20	21	22	23	24	25	26	27	28	29	30	31	32	33
**1**	**PP320363**	ID	0.96	0.964	0.964	0.996	0.964	0.946	0.896	0.9	0.971	0.96	0.96	0.978	0.921	0.96	0.975	0.964	0.967	0.978	0.854	0.825	0.85	0.96	0.868	0.886	0.882	0.843	0.825	0.836	0.85	0.864	0.871	0.846
**2**	**PP320367**	0.96	ID	0.953	0.946	0.957	0.939	0.943	0.9	0.896	0.946	0.939	0.939	0.953	0.914	0.932	0.95	0.953	0.95	0.953	0.85	0.829	0.857	0.935	0.871	0.882	0.864	0.832	0.822	0.832	0.846	0.861	0.861	0.85
**3**	**PP320368**	0.964	0.953	ID	0.957	0.96	0.943	0.939	0.889	0.893	0.964	0.946	0.946	0.964	0.928	0.95	0.96	0.95	0.953	0.964	0.839	0.822	0.85	0.95	0.864	0.889	0.871	0.829	0.811	0.825	0.846	0.85	0.868	0.836
**4**	**PP320362**	0.964	0.946	0.957	ID	0.964	0.96	0.928	0.889	0.893	0.964	0.943	0.943	0.971	0.925	0.946	0.967	0.964	0.96	0.971	0.839	0.829	0.843	0.95	0.864	0.879	0.861	0.818	0.8	0.814	0.85	0.843	0.857	0.836
**5**	**PP320369**	0.996	0.957	0.96	0.964	ID	0.964	0.943	0.893	0.896	0.971	0.96	0.96	0.978	0.918	0.96	0.975	0.964	0.967	0.978	0.85	0.822	0.846	0.96	0.864	0.882	0.879	0.839	0.822	0.832	0.846	0.861	0.868	0.843
**6**	**PP320364**	0.964	0.939	0.943	0.96	0.964	ID	0.932	0.886	0.896	0.95	0.935	0.935	0.971	0.907	0.939	0.967	0.943	0.953	0.964	0.846	0.825	0.836	0.943	0.861	0.886	0.857	0.822	0.804	0.818	0.857	0.864	0.868	0.836
**7**	**PP320365**	0.946	0.943	0.939	0.928	0.943	0.932	ID	0.886	0.889	0.932	0.921	0.921	0.939	0.911	0.928	0.943	0.932	0.928	0.939	0.843	0.814	0.857	0.921	0.854	0.879	0.846	0.836	0.818	0.829	0.843	0.864	0.857	0.836
**8**	**PP320366**	0.896	0.9	0.889	0.889	0.893	0.886	0.886	ID	0.982	0.886	0.879	0.879	0.889	0.868	0.875	0.896	0.896	0.889	0.893	0.893	0.889	0.921	0.879	0.914	0.932	0.839	0.882	0.878	0.896	0.882	0.893	0.921	0.911
**9**	**PP320370**	0.9	0.896	0.893	0.893	0.896	0.896	0.889	0.982	ID	0.889	0.882	0.882	0.893	0.864	0.875	0.9	0.9	0.886	0.896	0.903	0.896	0.918	0.882	0.911	0.943	0.846	0.896	0.879	0.893	0.893	0.903	0.935	0.914
**10**	**PP320361**	0.971	0.946	0.964	0.964	0.971	0.95	0.932	0.886	0.889	ID	0.957	0.957	0.971	0.907	0.96	0.975	0.957	0.975	0.985	0.839	0.811	0.832	0.95	0.854	0.871	0.868	0.839	0.825	0.836	0.836	0.843	0.857	0.832
**11**	**PP320359**	0.96	0.939	0.946	0.943	0.96	0.935	0.921	0.879	0.882	0.957	ID	1	0.95	0.911	0.939	0.946	0.935	0.946	0.957	0.829	0.814	0.843	0.946	0.857	0.868	0.85	0.836	0.822	0.832	0.825	0.832	0.861	0.846
**12**	**PP320360**	0.96	0.939	0.946	0.943	0.96	0.935	0.921	0.879	0.882	0.957	1	ID	0.95	0.911	0.939	0.946	0.935	0.946	0.957	0.829	0.814	0.843	0.946	0.857	0.868	0.85	0.836	0.822	0.832	0.825	0.832	0.861	0.846
**13**	**PP320358**	0.978	0.953	0.964	0.971	0.978	0.971	0.939	0.889	0.893	0.971	0.95	0.95	ID	0.928	0.96	0.982	0.964	0.975	0.985	0.854	0.825	0.85	0.964	0.875	0.886	0.882	0.836	0.818	0.829	0.857	0.864	0.864	0.846
**14**	**OM451182.1**	0.921	0.914	0.928	0.925	0.918	0.907	0.911	0.868	0.864	0.907	0.911	0.911	0.928	ID	0.9	0.918	0.911	0.925	0.921	0.85	0.854	0.882	0.914	0.893	0.871	0.871	0.843	0.832	0.857	0.861	0.857	0.875	0.871
**15**	**MN449341.1**	0.96	0.932	0.95	0.946	0.96	0.939	0.928	0.875	0.875	0.96	0.939	0.939	0.96	0.9	ID	0.957	0.939	0.96	0.96	0.843	0.804	0.836	0.953	0.854	0.868	0.864	0.825	0.811	0.822	0.825	0.839	0.854	0.825
**16**	**MK201779.1**	0.975	0.95	0.96	0.967	0.975	0.967	0.943	0.896	0.9	0.975	0.946	0.946	0.982	0.918	0.957	ID	0.967	0.978	0.989	0.846	0.818	0.843	0.96	0.861	0.879	0.875	0.829	0.811	0.822	0.85	0.864	0.857	0.832
**17**	**MK201778.1**	0.964	0.953	0.95	0.964	0.964	0.943	0.932	0.896	0.9	0.957	0.935	0.935	0.964	0.911	0.939	0.967	ID	0.953	0.964	0.839	0.832	0.839	0.95	0.861	0.886	0.868	0.829	0.811	0.822	0.85	0.864	0.857	0.839
**18**	**MK201776.1**	0.967	0.95	0.953	0.96	0.967	0.953	0.928	0.889	0.886	0.975	0.946	0.946	0.975	0.925	0.96	0.978	0.953	ID	0.989	0.843	0.814	0.839	0.96	0.871	0.868	0.871	0.822	0.814	0.825	0.839	0.854	0.846	0.829
**19**	**MK201777.1**	0.978	0.953	0.964	0.971	0.978	0.964	0.939	0.893	0.896	0.985	0.957	0.957	0.985	0.921	0.96	0.989	0.964	0.989	ID	0.846	0.818	0.843	0.964	0.861	0.879	0.875	0.832	0.818	0.829	0.843	0.857	0.857	0.832
**20**	**MT610361.1**	0.854	0.85	0.839	0.839	0.85	0.846	0.843	0.893	0.903	0.839	0.829	0.829	0.854	0.85	0.843	0.846	0.839	0.843	0.846	ID	0.882	0.9	0.839	0.925	0.911	0.871	0.882	0.864	0.886	0.928	0.9	0.921	0.9
**21**	**MH747478.1**	0.825	0.829	0.822	0.829	0.822	0.825	0.814	0.889	0.896	0.811	0.814	0.814	0.825	0.854	0.804	0.818	0.832	0.814	0.818	0.882	ID	0.903	0.818	0.911	0.903	0.832	0.886	0.875	0.893	0.896	0.882	0.935	0.921
**22**	**MN337880.1**	0.85	0.857	0.85	0.843	0.846	0.836	0.857	0.921	0.918	0.832	0.843	0.843	0.85	0.882	0.836	0.843	0.839	0.839	0.843	0.9	0.903	ID	0.836	0.925	0.907	0.836	0.911	0.9	0.921	0.889	0.879	0.935	0.946
**23**	**MF062158.1**	0.96	0.935	0.95	0.95	0.96	0.943	0.921	0.879	0.882	0.95	0.946	0.946	0.964	0.914	0.953	0.96	0.95	0.96	0.964	0.839	0.818	0.836	ID	0.868	0.879	0.868	0.829	0.811	0.822	0.829	0.85	0.85	0.832
**24**	**KF924623.1**	0.868	0.871	0.864	0.864	0.864	0.861	0.854	0.914	0.911	0.854	0.857	0.857	0.875	0.893	0.854	0.861	0.861	0.871	0.861	0.925	0.911	0.925	0.868	ID	0.95	0.854	0.886	0.875	0.903	0.935	0.921	0.946	0.939
**25**	**MH052678.1**	0.886	0.882	0.889	0.879	0.882	0.886	0.879	0.932	0.943	0.871	0.868	0.868	0.886	0.871	0.868	0.879	0.886	0.868	0.879	0.911	0.903	0.907	0.879	0.95	ID	0.861	0.889	0.871	0.893	0.935	0.939	0.95	0.928
**26**	**JQ911763.1**	0.882	0.864	0.871	0.861	0.879	0.857	0.846	0.839	0.846	0.868	0.85	0.85	0.882	0.871	0.864	0.875	0.868	0.871	0.875	0.871	0.832	0.836	0.868	0.854	0.861	ID	0.854	0.836	0.822	0.871	0.857	0.857	0.836
**27**	**KM068050.1**	0.843	0.832	0.829	0.818	0.839	0.822	0.836	0.882	0.896	0.839	0.836	0.836	0.836	0.843	0.825	0.829	0.829	0.822	0.832	0.882	0.886	0.911	0.829	0.886	0.889	0.854	ID	0.978	0.95	0.871	0.882	0.907	0.918
**28**	**KM068049.1**	0.825	0.822	0.811	0.8	0.822	0.804	0.818	0.878	0.879	0.825	0.822	0.822	0.818	0.832	0.811	0.811	0.811	0.814	0.818	0.864	0.875	0.9	0.811	0.875	0.871	0.836	0.978	ID	0.957	0.854	0.864	0.889	0.907
**29**	**KM068051.1**	0.836	0.832	0.825	0.814	0.832	0.818	0.829	0.896	0.893	0.836	0.832	0.832	0.829	0.857	0.822	0.822	0.822	0.825	0.829	0.886	0.893	0.921	0.822	0.903	0.893	0.822	0.95	0.957	ID	0.871	0.868	0.918	0.95
**30**	**JN088541.1**	0.85	0.846	0.846	0.85	0.846	0.857	0.843	0.882	0.893	0.836	0.825	0.825	0.857	0.861	0.825	0.85	0.85	0.839	0.843	0.928	0.896	0.889	0.829	0.935	0.935	0.871	0.871	0.854	0.871	ID	0.932	0.928	0.9
**31**	**JN387133.1**	0.864	0.861	0.85	0.843	0.861	0.864	0.864	0.893	0.903	0.843	0.832	0.832	0.864	0.857	0.839	0.864	0.864	0.854	0.857	0.9	0.882	0.879	0.85	0.921	0.939	0.857	0.882	0.864	0.868	0.932	ID	0.921	0.896
**32**	**KC161964.1**	0.871	0.861	0.868	0.857	0.868	0.868	0.857	0.921	0.935	0.857	0.861	0.861	0.864	0.875	0.854	0.857	0.857	0.846	0.857	0.921	0.935	0.935	0.85	0.946	0.95	0.857	0.907	0.889	0.918	0.928	0.921	ID	0.946
**33**	**KM068048.1**	0.846	0.85	0.836	0.836	0.843	0.836	0.836	0.911	0.914	0.832	0.846	0.846	0.846	0.871	0.825	0.832	0.839	0.829	0.832	0.9	0.921	0.946	0.832	0.939	0.928	0.836	0.918	0.907	0.95	0.9	0.896	0.946	ID

Supplementary Table 9l Nucleotide sequence similarities of the CaKoV 3D gene

	Sequences	1	2	3	4	5	6	7	8	9	10	11	12	13	14	15	16	17	18	19	20	21	22	23	24	25	26	27	28	29	30	31	32	33
**1**	**PP320363**	ID	0.976	0.975	0.975	0.977	0.978	0.977	0.972	0.972	0.967	0.97	0.97	0.969	0.978	0.978	0.976	0.976	0.977	0.97	0.97	0.966	0.962	0.975	0.959	0.962	0.976	0.95	0.95	0.955	0.955	0.964	0.971	0.957
**2**	**PP320367**	0.976	ID	0.973	0.973	0.978	0.98	0.976	0.971	0.973	0.973	0.973	0.973	0.973	0.977	0.982	0.98	0.982	0.976	0.971	0.969	0.969	0.961	0.976	0.964	0.964	0.975	0.944	0.941	0.949	0.956	0.957	0.962	0.949
**3**	**PP320368**	0.975	0.973	ID	0.976	0.982	0.983	0.975	0.975	0.98	0.97	0.967	0.967	0.965	0.978	0.978	0.976	0.981	0.975	0.975	0.971	0.967	0.954	0.977	0.954	0.965	0.973	0.947	0.947	0.952	0.957	0.961	0.961	0.952
**4**	**PP320362**	0.975	0.973	0.976	ID	0.981	0.978	0.977	0.967	0.972	0.967	0.97	0.97	0.962	0.977	0.978	0.976	0.978	0.973	0.969	0.971	0.96	0.956	0.977	0.956	0.959	0.975	0.946	0.944	0.951	0.952	0.955	0.964	0.949
**5**	**PP320369**	0.977	0.978	0.982	0.981	ID	0.983	0.982	0.972	0.98	0.975	0.972	0.972	0.965	0.981	0.983	0.983	0.986	0.982	0.977	0.973	0.965	0.959	0.985	0.959	0.972	0.986	0.952	0.947	0.957	0.96	0.964	0.966	0.955
**6**	**PP320364**	0.978	0.98	0.983	0.978	0.983	ID	0.981	0.978	0.981	0.976	0.976	0.976	0.971	0.98	0.985	0.985	0.987	0.978	0.976	0.976	0.969	0.959	0.981	0.957	0.969	0.977	0.946	0.946	0.951	0.956	0.96	0.967	0.954
**7**	**PP320365**	0.977	0.976	0.975	0.977	0.982	0.981	ID	0.97	0.972	0.972	0.97	0.97	0.965	0.973	0.976	0.976	0.981	0.977	0.97	0.97	0.962	0.957	0.982	0.956	0.964	0.978	0.942	0.945	0.947	0.952	0.956	0.959	0.95
**8**	**PP320366**	0.972	0.971	0.975	0.967	0.972	0.978	0.97	ID	0.972	0.966	0.967	0.967	0.967	0.973	0.973	0.973	0.976	0.975	0.967	0.967	0.961	0.957	0.97	0.946	0.96	0.971	0.94	0.942	0.945	0.952	0.956	0.959	0.947
**9**	**PP320370**	0.972	0.973	0.98	0.972	0.98	0.981	0.972	0.972	ID	0.97	0.97	0.97	0.967	0.976	0.976	0.978	0.978	0.975	0.972	0.972	0.965	0.957	0.977	0.951	0.965	0.971	0.947	0.945	0.952	0.955	0.961	0.965	0.95
**10**	**PP320361**	0.967	0.973	0.97	0.967	0.975	0.976	0.972	0.966	0.97	ID	0.981	0.981	0.967	0.969	0.978	0.983	0.976	0.967	0.962	0.967	0.965	0.957	0.971	0.95	0.959	0.973	0.942	0.94	0.947	0.955	0.959	0.962	0.949
**11**	**PP320359**	0.97	0.973	0.967	0.97	0.972	0.976	0.97	0.967	0.97	0.981	ID	1	0.97	0.969	0.978	0.983	0.976	0.97	0.962	0.965	0.957	0.955	0.97	0.951	0.957	0.973	0.945	0.94	0.95	0.952	0.956	0.961	0.947
**12**	**PP320360**	0.97	0.973	0.967	0.97	0.972	0.976	0.97	0.967	0.97	0.981	1	ID	0.97	0.969	0.978	0.983	0.976	0.97	0.962	0.965	0.957	0.955	0.97	0.951	0.957	0.973	0.945	0.94	0.95	0.952	0.956	0.961	0.947
**13**	**PP320358**	0.969	0.973	0.965	0.962	0.965	0.971	0.965	0.967	0.967	0.967	0.97	0.97	ID	0.969	0.973	0.971	0.971	0.967	0.96	0.96	0.969	0.955	0.962	0.95	0.952	0.966	0.94	0.938	0.945	0.955	0.959	0.956	0.947
**14**	**OM451182.1**	0.978	0.977	0.978	0.977	0.981	0.98	0.973	0.973	0.976	0.969	0.969	0.969	0.969	ID	0.98	0.977	0.98	0.978	0.973	0.972	0.967	0.96	0.976	0.955	0.964	0.975	0.949	0.949	0.954	0.959	0.962	0.967	0.954
**15**	**MN449341.1**	0.978	0.982	0.978	0.978	0.983	0.985	0.976	0.973	0.976	0.978	0.978	0.978	0.973	0.98	ID	0.99	0.987	0.978	0.973	0.976	0.969	0.959	0.978	0.96	0.966	0.982	0.949	0.944	0.954	0.959	0.962	0.965	0.951
**16**	**MK201779.1**	0.976	0.98	0.976	0.976	0.983	0.985	0.976	0.973	0.978	0.983	0.983	0.983	0.971	0.977	0.99	ID	0.987	0.978	0.973	0.973	0.966	0.959	0.978	0.96	0.969	0.982	0.946	0.941	0.951	0.956	0.962	0.965	0.951
**17**	**MK201778.1**	0.976	0.982	0.981	0.978	0.986	0.987	0.981	0.976	0.978	0.976	0.976	0.976	0.971	0.98	0.987	0.987	ID	0.978	0.975	0.976	0.969	0.956	0.981	0.964	0.969	0.982	0.949	0.946	0.954	0.956	0.96	0.964	0.951
**18**	**MK201776.1**	0.977	0.976	0.975	0.973	0.982	0.978	0.977	0.975	0.975	0.967	0.97	0.97	0.967	0.978	0.978	0.978	0.978	ID	0.987	0.973	0.967	0.956	0.977	0.954	0.97	0.978	0.947	0.947	0.952	0.957	0.961	0.964	0.957
**19**	**MK201777.1**	0.97	0.971	0.975	0.969	0.977	0.976	0.97	0.967	0.972	0.962	0.962	0.962	0.96	0.973	0.973	0.973	0.975	0.987	ID	0.969	0.965	0.951	0.97	0.946	0.965	0.971	0.945	0.945	0.95	0.952	0.956	0.964	0.95
**20**	**MT610361.1**	0.97	0.969	0.971	0.971	0.973	0.976	0.97	0.967	0.972	0.967	0.965	0.965	0.96	0.972	0.976	0.973	0.976	0.973	0.969	ID	0.959	0.954	0.97	0.949	0.959	0.97	0.945	0.945	0.95	0.952	0.954	0.964	0.952
**21**	**MH747478.1**	0.966	0.969	0.967	0.96	0.965	0.969	0.962	0.961	0.965	0.965	0.957	0.957	0.969	0.967	0.969	0.966	0.969	0.967	0.965	0.959	ID	0.961	0.964	0.944	0.95	0.959	0.95	0.951	0.955	0.956	0.961	0.962	0.956
**22**	**MN337880.1**	0.962	0.961	0.954	0.956	0.959	0.959	0.957	0.957	0.957	0.957	0.955	0.955	0.955	0.96	0.959	0.959	0.956	0.956	0.951	0.954	0.961	ID	0.956	0.935	0.944	0.95	0.941	0.941	0.946	0.96	0.961	0.959	0.949
**23**	**MF062158.1**	0.975	0.976	0.977	0.977	0.985	0.981	0.982	0.97	0.977	0.971	0.97	0.97	0.962	0.976	0.978	0.978	0.981	0.977	0.97	0.97	0.964	0.956	ID	0.959	0.965	0.976	0.944	0.941	0.949	0.954	0.957	0.964	0.949
**24**	**KF924623.1**	0.959	0.964	0.954	0.956	0.959	0.957	0.956	0.946	0.951	0.95	0.951	0.951	0.95	0.955	0.96	0.96	0.964	0.954	0.946	0.949	0.944	0.935	0.959	ID	0.941	0.955	0.925	0.923	0.93	0.93	0.938	0.94	0.928
**25**	**MH052678.1**	0.962	0.964	0.965	0.959	0.972	0.969	0.964	0.96	0.965	0.959	0.957	0.957	0.952	0.964	0.966	0.969	0.969	0.97	0.965	0.959	0.95	0.944	0.965	0.941	ID	0.97	0.94	0.94	0.945	0.947	0.951	0.949	0.95
**26**	**JQ911763.1**	0.976	0.975	0.973	0.975	0.986	0.977	0.978	0.971	0.971	0.973	0.973	0.973	0.966	0.975	0.982	0.982	0.982	0.978	0.971	0.97	0.959	0.95	0.976	0.955	0.97	ID	0.951	0.944	0.954	0.954	0.957	0.96	0.949
**27**	**KM068050.1**	0.95	0.944	0.947	0.946	0.952	0.946	0.942	0.94	0.947	0.942	0.945	0.945	0.94	0.949	0.949	0.946	0.949	0.947	0.945	0.945	0.95	0.941	0.944	0.925	0.94	0.951	ID	0.98	0.99	0.952	0.952	0.949	0.952
**28**	**KM068049.1**	0.95	0.941	0.947	0.944	0.947	0.946	0.945	0.942	0.945	0.94	0.94	0.94	0.938	0.949	0.944	0.941	0.946	0.947	0.945	0.945	0.951	0.941	0.941	0.923	0.94	0.944	0.98	ID	0.977	0.952	0.955	0.946	0.97
**29**	**KM068051.1**	0.955	0.949	0.952	0.951	0.957	0.951	0.947	0.945	0.952	0.947	0.95	0.95	0.945	0.954	0.954	0.951	0.954	0.952	0.95	0.95	0.955	0.946	0.949	0.93	0.945	0.954	0.99	0.977	ID	0.955	0.957	0.954	0.957
**30**	**JN088541.1**	0.955	0.956	0.957	0.952	0.96	0.956	0.952	0.952	0.955	0.955	0.952	0.952	0.955	0.959	0.959	0.956	0.956	0.957	0.952	0.952	0.956	0.96	0.954	0.93	0.947	0.954	0.952	0.952	0.955	ID	0.975	0.956	0.955
**31**	**JN387133.1**	0.964	0.957	0.961	0.955	0.964	0.96	0.956	0.956	0.961	0.959	0.956	0.956	0.959	0.962	0.962	0.962	0.96	0.961	0.956	0.954	0.961	0.961	0.957	0.938	0.951	0.957	0.952	0.955	0.957	0.975	ID	0.962	0.962
**32**	**KC161964.1**	0.971	0.962	0.961	0.964	0.966	0.967	0.959	0.959	0.965	0.962	0.961	0.961	0.956	0.967	0.965	0.965	0.964	0.964	0.964	0.964	0.962	0.959	0.964	0.94	0.949	0.96	0.949	0.946	0.954	0.956	0.962	ID	0.954
**33**	**KM068048.1**	0.957	0.949	0.952	0.949	0.955	0.954	0.95	0.947	0.95	0.949	0.947	0.947	0.947	0.954	0.951	0.951	0.951	0.957	0.95	0.952	0.956	0.949	0.949	0.928	0.95	0.949	0.952	0.97	0.957	0.955	0.962	0.954	ID

Supplementary Table 9m Deduced amino acid sequence similarities of the CaKoV 3D gene

	Sequences	1	2	3	4	5	6	7	8	9	10	11	12	13	14	15	16	17	18	19	20	21	22	23	24	25	26	27	28	29	30	31	32	33
**1**	**PP320363**	ID	1	1	0.996	1	1	1	0.996	1	0.996	1	1	1	0.988	0.996	1	1	1	1	1	0.996	0.992	1	0.985	0.996	1	0.988	0.988	0.992	0.996	0.992	1	0.996
**2**	**PP320367**	1	ID	1	0.996	1	1	1	0.996	1	0.996	1	1	1	0.988	0.996	1	1	1	1	1	0.996	0.992	1	0.985	0.996	1	0.988	0.988	0.992	0.996	0.992	1	0.996
**3**	**PP320368**	1	1	ID	0.996	1	1	1	0.996	1	0.996	1	1	1	0.988	0.996	1	1	1	1	1	0.996	0.992	1	0.985	0.996	1	0.988	0.988	0.992	0.996	0.992	1	0.996
**4**	**PP320362**	0.996	0.996	0.996	ID	0.996	0.996	0.996	0.992	0.996	0.992	0.996	0.996	0.996	0.985	0.992	0.996	0.996	0.996	0.996	0.996	0.992	0.988	0.996	0.981	0.992	0.996	0.985	0.985	0.988	0.992	0.988	0.996	0.992
**5**	**PP320369**	1	1	1	0.996	ID	1	1	0.996	1	0.996	1	1	1	0.988	0.996	1	1	1	1	1	0.996	0.992	1	0.985	0.996	1	0.988	0.988	0.992	0.996	0.992	1	0.996
**6**	**PP320364**	1	1	1	0.996	1	ID	1	0.996	1	0.996	1	1	1	0.988	0.996	1	1	1	1	1	0.996	0.992	1	0.985	0.996	1	0.988	0.988	0.992	0.996	0.992	1	0.996
**7**	**PP320365**	1	1	1	0.996	1	1	ID	0.996	1	0.996	1	1	1	0.988	0.996	1	1	1	1	1	0.996	0.992	1	0.985	0.996	1	0.988	0.988	0.992	0.996	0.992	1	0.996
**8**	**PP320366**	0.996	0.996	0.996	0.992	0.996	0.996	0.996	ID	0.996	0.992	0.996	0.996	0.996	0.985	0.992	0.996	0.996	0.996	0.996	0.996	0.992	0.996	0.996	0.981	0.992	0.996	0.985	0.985	0.988	0.992	0.988	0.996	0.992
**9**	**PP320370**	1	1	1	0.996	1	1	1	0.996	ID	0.996	1	1	1	0.988	0.996	1	1	1	1	1	0.996	0.992	1	0.985	0.996	1	0.988	0.988	0.992	0.996	0.992	1	0.996
**10**	**PP320361**	0.996	0.996	0.996	0.992	0.996	0.996	0.996	0.992	0.996	ID	0.996	0.996	0.996	0.985	0.992	0.996	0.996	0.996	0.996	0.996	1	0.996	0.996	0.981	0.992	0.996	0.992	0.992	0.992	1	0.996	0.996	1
**11**	**PP320359**	1	1	1	0.996	1	1	1	0.996	1	0.996	ID	1	1	0.988	0.996	1	1	1	1	1	0.996	0.992	1	0.985	0.996	1	0.988	0.988	0.992	0.996	0.992	1	0.996
**12**	**PP320360**	1	1	1	0.996	1	1	1	0.996	1	0.996	1	ID	1	0.988	0.996	1	1	1	1	1	0.996	0.992	1	0.985	0.996	1	0.988	0.988	0.992	0.996	0.992	1	0.996
**13**	**PP320358**	1	1	1	0.996	1	1	1	0.996	1	0.996	1	1	ID	0.988	0.996	1	1	1	1	1	0.996	0.992	1	0.985	0.996	1	0.988	0.988	0.992	0.996	0.992	1	0.996
**14**	**OM451182.1**	0.988	0.988	0.988	0.985	0.988	0.988	0.988	0.985	0.988	0.985	0.988	0.988	0.988	ID	0.985	0.988	0.988	0.988	0.988	0.988	0.985	0.981	0.988	0.973	0.985	0.988	0.977	0.977	0.981	0.985	0.981	0.988	0.985
**15**	**MN449341.1**	0.996	0.996	0.996	0.992	0.996	0.996	0.996	0.992	0.996	0.992	0.996	0.996	0.996	0.985	ID	0.996	0.996	0.996	0.996	0.996	0.992	0.988	0.996	0.981	0.992	0.996	0.985	0.985	0.988	0.992	0.988	0.996	0.992
**16**	**MK201779.1**	1	1	1	0.996	1	1	1	0.996	1	0.996	1	1	1	0.988	0.996	ID	1	1	1	1	0.996	0.992	1	0.985	0.996	1	0.988	0.988	0.992	0.996	0.992	1	0.996
**17**	**MK201778.1**	1	1	1	0.996	1	1	1	0.996	1	0.996	1	1	1	0.988	0.996	1	ID	1	1	1	0.996	0.992	1	0.985	0.996	1	0.988	0.988	0.992	0.996	0.992	1	0.996
**18**	**MK201776.1**	1	1	1	0.996	1	1	1	0.996	1	0.996	1	1	1	0.988	0.996	1	1	ID	1	1	0.996	0.992	1	0.985	0.996	1	0.988	0.988	0.992	0.996	0.992	1	0.996
**19**	**MK201777.1**	1	1	1	0.996	1	1	1	0.996	1	0.996	1	1	1	0.988	0.996	1	1	1	ID	1	0.996	0.992	1	0.985	0.996	1	0.988	0.988	0.992	0.996	0.992	1	0.996
**20**	**MT610361.1**	1	1	1	0.996	1	1	1	0.996	1	0.996	1	1	1	0.988	0.996	1	1	1	1	ID	0.996	0.992	1	0.985	0.996	1	0.988	0.988	0.992	0.996	0.992	1	0.996
**21**	**MH747478.1**	0.996	0.996	0.996	0.992	0.996	0.996	0.996	0.992	0.996	1	0.996	0.996	0.996	0.985	0.992	0.996	0.996	0.996	0.996	0.996	ID	0.996	0.996	0.981	0.992	0.996	0.992	0.992	0.992	1	0.996	0.996	1
**22**	**MN337880.1**	0.992	0.992	0.992	0.988	0.992	0.992	0.992	0.996	0.992	0.996	0.992	0.992	0.992	0.981	0.988	0.992	0.992	0.992	0.992	0.992	0.996	ID	0.992	0.977	0.988	0.992	0.988	0.988	0.988	0.996	0.992	0.992	0.996
**23**	**MF062158.1**	1	1	1	0.996	1	1	1	0.996	1	0.996	1	1	1	0.988	0.996	1	1	1	1	1	0.996	0.992	ID	0.985	0.996	1	0.988	0.988	0.992	0.996	0.992	1	0.996
**24**	**KF924623.1**	0.985	0.985	0.985	0.981	0.985	0.985	0.985	0.981	0.985	0.981	0.985	0.985	0.985	0.973	0.981	0.985	0.985	0.985	0.985	0.985	0.981	0.977	0.985	ID	0.981	0.985	0.973	0.973	0.977	0.981	0.977	0.985	0.981
**25**	**MH052678.1**	0.996	0.996	0.996	0.992	0.996	0.996	0.996	0.992	0.996	0.992	0.996	0.996	0.996	0.985	0.992	0.996	0.996	0.996	0.996	0.996	0.992	0.988	0.996	0.981	ID	0.996	0.985	0.985	0.988	0.992	0.988	0.996	0.992
**26**	**JQ911763.1**	1	1	1	0.996	1	1	1	0.996	1	0.996	1	1	1	0.988	0.996	1	1	1	1	1	0.996	0.992	1	0.985	0.996	ID	0.988	0.988	0.992	0.996	0.992	1	0.996
**27**	**KM068050.1**	0.988	0.988	0.988	0.985	0.988	0.988	0.988	0.985	0.988	0.992	0.988	0.988	0.988	0.977	0.985	0.988	0.988	0.988	0.988	0.988	0.992	0.988	0.988	0.973	0.985	0.988	ID	0.992	0.992	0.992	0.988	0.988	0.992
**28**	**KM068049.1**	0.988	0.988	0.988	0.985	0.988	0.988	0.988	0.985	0.988	0.992	0.988	0.988	0.988	0.977	0.985	0.988	0.988	0.988	0.988	0.988	0.992	0.988	0.988	0.973	0.985	0.988	0.992	ID	0.992	0.992	0.988	0.988	0.992
**29**	**KM068051.1**	0.992	0.992	0.992	0.988	0.992	0.992	0.992	0.988	0.992	0.992	0.992	0.992	0.992	0.981	0.988	0.992	0.992	0.992	0.992	0.992	0.992	0.988	0.992	0.977	0.988	0.992	0.992	0.992	ID	0.992	0.988	0.992	0.992
**30**	**JN088541.1**	0.996	0.996	0.996	0.992	0.996	0.996	0.996	0.992	0.996	1	0.996	0.996	0.996	0.985	0.992	0.996	0.996	0.996	0.996	0.996	1	0.996	0.996	0.981	0.992	0.996	0.992	0.992	0.992	ID	0.996	0.996	1
**31**	**JN387133.1**	0.992	0.992	0.992	0.988	0.992	0.992	0.992	0.988	0.992	0.996	0.992	0.992	0.992	0.981	0.988	0.992	0.992	0.992	0.992	0.992	0.996	0.992	0.992	0.977	0.988	0.992	0.988	0.988	0.988	0.996	ID	0.992	0.996
**32**	**KC161964.1**	1	1	1	0.996	1	1	1	0.996	1	0.996	1	1	1	0.988	0.996	1	1	1	1	1	0.996	0.992	1	0.985	0.996	1	0.988	0.988	0.992	0.996	0.992	ID	0.996
**33**	**KM068048.1**	0.996	0.996	0.996	0.992	0.996	0.996	0.996	0.992	0.996	1	0.996	0.996	0.996	0.985	0.992	0.996	0.996	0.996	0.996	0.996	1	0.996	0.996	0.981	0.992	0.996	0.992	0.992	0.992	1	0.996	0.996	ID
